# Flame-Made Surface-Substituted Copper–Ceria as an Excellent Reverse Water–Gas Shift Reaction Catalyst via Three Reaction Pathways

**DOI:** 10.1021/jacs.5c07701

**Published:** 2025-09-01

**Authors:** Bingqiao Xie, Yi Fen Zhu, Mahdi Shakeri, Seongmin Jin, George O’Connell, Sankhadip Saha, Mounir Mensi, Priyank V. Kumar, Jeremy S. Luterbacher, Emma C. Lovell, Rose Amal, Oliver Kröcher

**Affiliations:** † Institute of Chemical Sciences and Engineering (ISIC), École Polytechnique Fédérale de Lausanne (EPFL), Lausanne 1015, Switzerland; ‡ School of Chemical Engineering, UNSW, Kensington, Sydney, NSW 2052, Australia; § Paul Scherrer Institute (PSI), PSI Center for Energy and Environmental Sciences, Villigen CH-5232, Switzerland

## Abstract

The limited mechanistic understanding and ambiguous structure–performance relationships have hindered the optimization of Cu-based catalysts for the reverse water–gas shift (rWGS) reaction. Here, we report a flame spray pyrolysis (FSP)-derived Cu–CeO_2_ catalyst featuring highly dispersed, surface-substituted Cu^+^ species (Cu*_*y*_* Ce_1–_ *_*y*_* O_2–_ *_*x*_*) anchored on a defect-rich ceria matrix. This catalyst demonstrates excellent stability and outstanding rWGS activity at 600 °C, achieving a CO production rate of 8094 mmol/g_cat._/h, surpassing the conventional Cu–CeO_2_ catalyst and other reported rWGS catalysts. In situ spectroscopic analyses, supported by DFT calculations, reveal three parallel reaction pathways in which carboxylate- and formate-mediated routes proceed at distinct active sites. A clear structure–activity correlation is established across Cu^+^, Cu^0^, and ceria defect sites in the FSP-derived catalysts. Notably, a previously underexplored carboxylate-mediated pathway, facilitated on the surface-substituted Cu^+^ structure, is identified as the dominant route, featuring a significantly lower apparent activation energy (20–30 kJ/mol) compared to the classical formate pathway.

## Graphical abstract

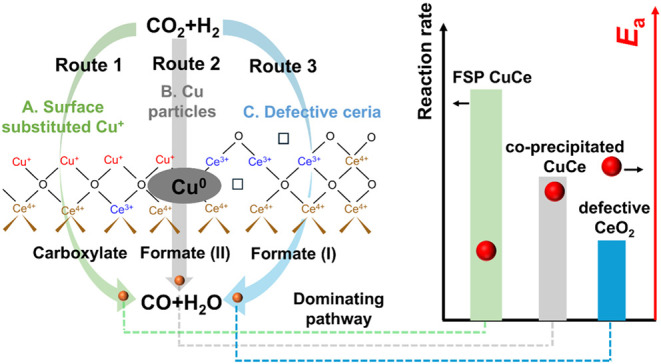

## Introduction

The reverse water–gas shift (rWGS) reaction (CO_2_ + H_2_ → CO + H_2_O) is a key process for CO_2_ utilization, producing CO as a critical intermediate for the Fischer–Tropsch reaction, methanol synthesis, and acetic acid production.^1,2^ As global efforts to mitigate carbon emissions intensify, the rWGS reaction has gained renewed attention for its role in CO_2_ recycling and the green fuel production.^3^ However, achieving high selectivity and activity under industrially relevant conditions remains challenging, particularly due to the high temperatures required to overcome thermodynamic limitations. Recent research has focused on developing advanced catalysts that enhance rWGS efficiency while maintaining stability under harsh reaction conditions.^3–5^

To break the carbon–oxygen bond in the CO_2_ molecule, two distinct mechanistic pathways have been identified for the rWGS reaction. The first class leverages strong CO_2_-catalyst interactions, wherein CO_2_ binds to the catalyst through both its carbon and oxygen atoms. This interaction facilitates C–O bond cleavage through a redox mechanism, with CO desorption identified as the most energy-intensive step. Recent studies on MoO_*x*_-based catalysts have highlighted the effectiveness of this mechanism.^3,4^ The second class follows an associative mechanism, which prevails on oxide-supported copper catalysts.^5,6^ Along this pathway, CO_2_ activation occurs on the oxide support, while H_2_ activation takes place on the metal. The activated hydrogen migrates to react with the adsorbed CO_2_ species, ultimately yielding CO.

Among the copper-based catalysts studied for rWGS, copper–ceria (Cu–CeO_2_) materials have demonstrated outstanding activity due to their unique structural and catalytic properties. Ceria, in particular, provides multiple functionalities critical for rWGS catalysis: (i) exposed defective sites on the CeO_2_ surface that facilitate catalytic CO_2_ activation and (ii) strong metal–support interactions that stabilize small metal clusters on its surface and prevent sintering.^7,8^ Copper is particularly well-suited for the rWGS reaction due to efficient H_2_ activation above 400 °C while maintaining a moderate CO binding affinity compared to noble metals like Pt and Pd. Recent studies have underscored the importance of high Cu dispersion on ceria. For example, Liu et al.^6^ prepared highly dispersed Cu clusters on ceria nanorods via deposition–precipitation, achieving impressive activity in the rWGS reaction at high temperatures. Optimizing Cu dispersion while avoiding bulk Cu–Ce solid solution formation or large Cu particles is therefore critical for maximizing the active sites density at the Cu–CeO_2_ interface.^9^

Despite these advances, key mechanistic aspects of Cu–CeO_2_ catalysts remain poorly understood, particularly the interplay between Cu oxidation states, surface defects, and hydrogen-mediated intermediate transformations. The complexity of heterogeneous catalysts—particularly those involving multiple active sites that perform distinct functions—poses a major challenge in establishing clear structure-performance relationships, thereby limiting our ability to optimize catalytic systems.^10^ While formate-mediated pathways are widely proposed for Cu-catalyzed CO_2_ rWGS,^11^ the potential role of carboxylate intermediates, previously implicated in water–gas shift (WGS) reactions^12^ and methanol synthesis^13^ on Cu–CeO_2_, has been largely overlooked in the context of rWGS reaction. Further mechanistic studies of Cu–CeO_2_ catalysts are required for a detailed understanding of the active sites involved at each step of the rWGS reaction.

Flame spray pyrolysis (FSP) offers a promising single-step, scalable alternative to conventional multistep solution-based methods especially for mixed oxide nanomaterials synthesis^14^ (Scheme 1). It is characterized by its short residence time, high flame temperature, and steep temperature gradients. Compared to conventional methods, these extreme conditions facilitate oxide defect formation/strong metal–support interactions, stabilize catalytically relevant species, and prevent the aggregation of metal into bulk particles.^14,15^ Recently, FSP-derived catalysts have exhibited remarkable performance in the CO_2_ hydrogenation to methanol (Table S1), with distinct structural features such as undercoordinated metal clusters,^15,16^ oxygen vacancy generation,^17^ and enhanced metal-oxide interactions, all contributing to the superior activity and stability compared to catalysts produced via other synthetic methods.

**Scheme 1:**
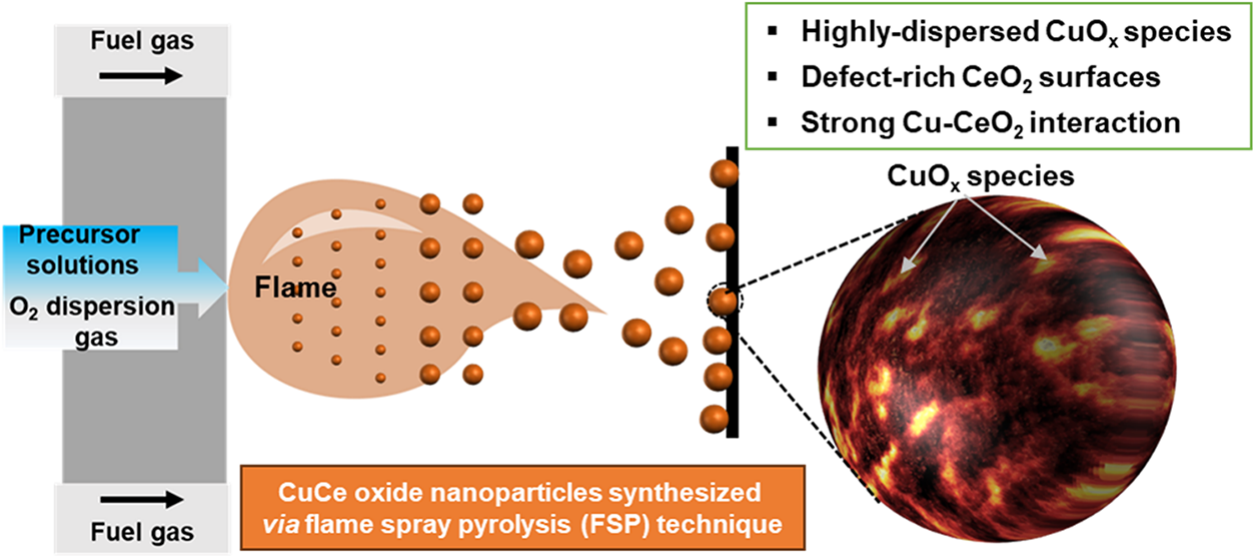
Illustration of the Flame Spray Pyrolysis (FSP) Technique for the Synthesis of Copper–Ceria Nanostructures with Desired Properties^a^

a The black rod represents the filter used to collect the product. The magnified panel on the right depicts the formation of highly dispersed CuO_*x*_ species (indicated by the arrow) on the ceria surface, either as clusters or small particles.

In this study, we exploit the advantages of the FSP technique to prepare highly dispersed Cu on CeO_2_ for the high-temperature rWGS reaction and demonstrate the critical role of multiple active sites in the system. The FSP-prepared Cu–CeO_2_ catalyst, characterized by surface-substituted Cu*_*y*_* Ce_1–_ *_*y*_* O_2–_ *_*x*_* and a defect-rich ceria support, displays superior activity with a notably lower activation barrier compared to conventionally prepared Cu–CeO_2_ and other rWGS catalysts reported in literature. Through in situ Raman and UV–vis spectroscopy, we track the evolution of ceria surface defects and copper species during the reaction, thereby elucidating the nature of key reactant–catalyst interactions. In addition, what distinguishes our work is the integration of advanced in situ spectroscopy and DFT modeling to identify three parallel rWGS pathways, and to quantitatively correlate active site (Cu^+^, Cu^0^, and defective sites) with catalytic performance. We further reveal the dominance of the carboxylate route over Cu^+^-substituted sites, which has not been previously well understood for Cu–CeO_2_ catalysts.

## Results and Discussion

### Activity and Stability of rWGS Catalysts

We investigated FSP-prepared Cu–CeO_2_ catalysts with nominal loadings ranging from 2.5 to 10 wt % and compared them with FSP-prepared CuSi (5CuSi-FSP) and coprecipitated CuCe (5CuCe-COP) catalysts. Table S2 summarizes the catalytic performance of our prepared catalysts compared with those reported in the literature, including Cu-based, Pt-based, and Mo-based catalysts. As shown in Figure S5a, all studied catalysts exhibited virtually 100% CO selectivity under the reaction conditions. However, FSP-prepared CuCe catalysts demonstrated significantly higher CO_2_ conversion (5CuCe-FSP: 58%, 2.5CuCe-FSP: 53.6%, 10CuCe-FSP: 53% at 600 °C and GHSV = 218,400 mL/g_cat._/h) compared to FSP-prepared CuSi (37.5%) and 5CuCe-COP (48.4%). Increasing the H_2_/CO_2_ ratio enhanced the CO_2_ conversion but reduced the CO productivity (CO production rate per gram of catalyst per unit time) (Figure S5b).

Figure 1a displays the Arrhenius plots for the rWGS reaction in the temperature range of 400–500 °C, showing the superior performance of the FSP-prepared CuCe in a different representation. The activation energy for 5CuCe-COP was 50.41 kJ/mol (Figure 1b), which is consistent with values reported for CuCe catalysts synthesized via solution-based method^6,18,19^ and within the range of other rWGS catalysts^20–22^ (50–80 kJ/mol). The kinetically relevant step is often attributed to the hydrogenation step (e.g., formate hydrogenation) for the associative pathway or CO desorption for the redox pathway.^21^ Interestingly, FSP-prepared CuCe catalysts exhibited activation energies between 20–30 kJ/mol, significantly lower than those reported for Cu-, Pt-, and Mo-oxycarbide-based catalysts. This suggests a distinct reaction pathway for FSP-prepared CuCe catalysts.

**Figure 1:**
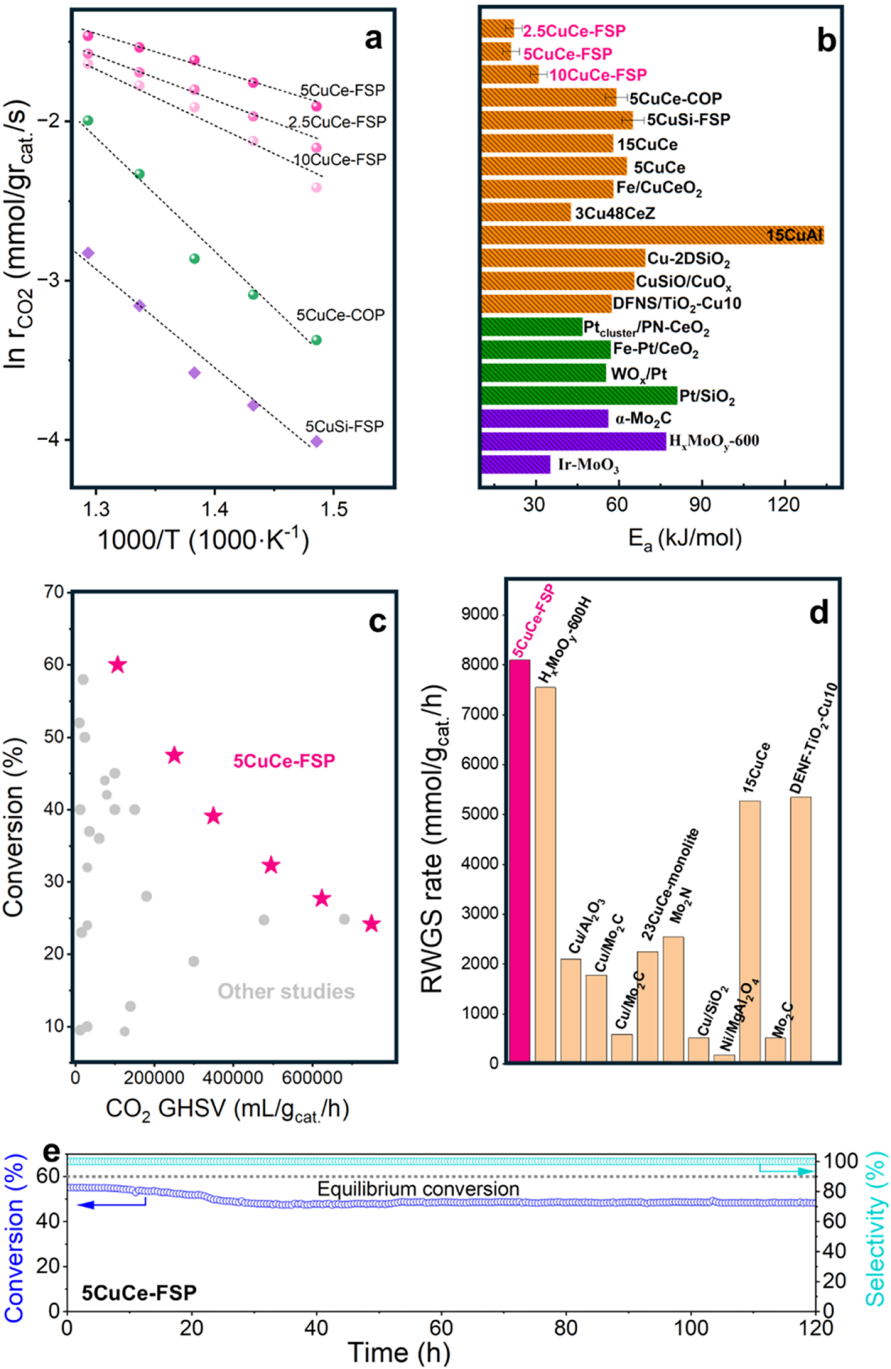
Catalytic rWGS performance of copper–ceria catalysts. (a) Arrhenius plot for rWGS reaction over Cu catalysts synthesized via flame spray pyrolysis (FSP) and coprecipitation (COP) methods. GHSV = 218400 mL/g_cat._/h. Comparison of (b) the apparent activation energy, (c) CO_2_ conversion at different CO_2_ GHSV for CuCe-FSP catalysts compared to Cu-based, Pt-based, and Mo_2_C-based rWGS catalysts reported in the literature (see Table S2 for details). (d) Comparison of catalytic performance of 5CuCe-FSP catalyst in this work (red) with different types of non-noble metal-based catalysts in the literature. (e) Performance stability of 5CuCe-FSP at 600 °C with GHSV = 376,800 mL/g/h.

We further evaluated the catalytic performance of 5CuCe-FSP under varying CO_2_ GHSVs at a fixed H_2_/CO_2_ ratio of 3. High space velocities, which reduce the reactant-catalyst contact time, resulted in lower conversions but increased the CO productivity (Figure S5c). Across a wide range of CO_2_ GHSVs (from 106,500 to 750,000 mL/g_cat._/h)—covering conditions studied in former rWGS studies—5CuCe-FSP showed relatively higher CO_2_ conversion rates compared to those reported in the literature (Figure 1c). Specifically, at a high CO_2_ GHSV of 750,000 mL/g_cat._/h, a CO_2_ conversion of 24.2% was achieved, corresponding to a CO productivity of 8094 mmol/g_cat._/h (Figure 1d and Table S2). This represents the highest productivity reported for non-noble metal-based rWGS catalysts to date. To assess the durability, 5CuCe-FSP was tested at 600 °C at a GHSV of 376,800 mL/g_cat._/h. Few percent activity drop in activity were observed during the first 40 h of operation, which is typical for Cu-based rWGS catalysts (e.g., 25.2% loss for DFNS/TiO_2_–Cu_10_ catalyst,^5^ 15% loss for 15CuCe catalyst^6^), whereafter the performance stabilized. Similarly, 5CuCe-FSP retained 91% of its initial activity after 120 h of continuous operation, in contrast to the significantly poor stability exhibited by CuCe-COP (Figure S6). Additional discussion on postreaction characterization of 5CuCe-FSP is included in the Supporting Discussion S4 in Supporting Information.

### Geometric Structure of Copper on Ceria

We now turn to understanding the structure of the FSP-prepared Cu–CeO_2_ catalysts by combining multiple characterization techniques. Figure S7 presents the X-ray diffraction (XRD) patterns of the FSP-prepared Cu–CeO_2_ catalysts, which shows that ceria nanoparticles are crystalline with an average size of 9.5 ± 1 nm. All Ce-containing samples exhibit distinct diffraction peaks corresponding to the typical face-centered cubic fluorite structure of CeO_2_ (PDF No. 00–004–0593). Notably, no additional peaks attributed to the Cu phase were observed in the patterns of the calcined CuCe-FSP sample, suggesting that CuO is well-dispersed and that large crystal particles are not present. The peak shape and position of the CeO_2_ diffraction peaks remain consistent across the FSP-prepared CuCe samples, ruling out the formation of a solid solution within the CeO_2_ lattice.^23^ The Cu content, as determined by ICP-OES and other physical properties of the catalysts, is summarized in Table S3. Scanning transmission electron microscopy (STEM) images in Figures 2 and S10–S12 reveal that the morphology of ceria changes as the Cu loading increases, indicating the formation of Cu–Ce interactions during the FSP synthesis (Figure 2a,f,k). Specifically, the ceria structure shifts from its original rhombohedral shape to polyhedral or spherical forms with higher Cu loading. Energy-dispersive X-ray spectroscopy (EDS) elemental mapping shows a uniform distribution of Cu across the CeO_2_. The size of Cu entities increases with the loading and Cu particles with a diameter of 5–10 nm can be detected in 10CuCe-FSP in Figure S8c-e. It was reported that, for copper–ceria with low copper loadings (i.e., <5 wt %), the CuO_*x*_ domains are highly dispersed and often anchored at the defect sites or step edges along ceria rods.^24,25^ The high-resolution HAADF-STEM images in Figure S8a,b further support this, showing subnanometric copper clusters on the ceria surface. These are characterized by misalignment and darker atoms compared to the brighter ceria region (indicated by orange arrows). In contrast, 5CuCe-COP exhibits poorly crystalline CeO_2_, as evidenced by the rounded particle morphology (Figure S9) and the continuous rings in the corresponding selected area diffraction (SAD) pattern. This suggests that the CuO_*x*_ particles may be encapsulated by poorly crystalline CeO_2_, a hypothesis supported by EDX mapping and XPS, which show a lower Cu/Ce ratio on the surface compared to the ICP results (0.14 vs 0.21) (Table S3).

**Figure 2:**
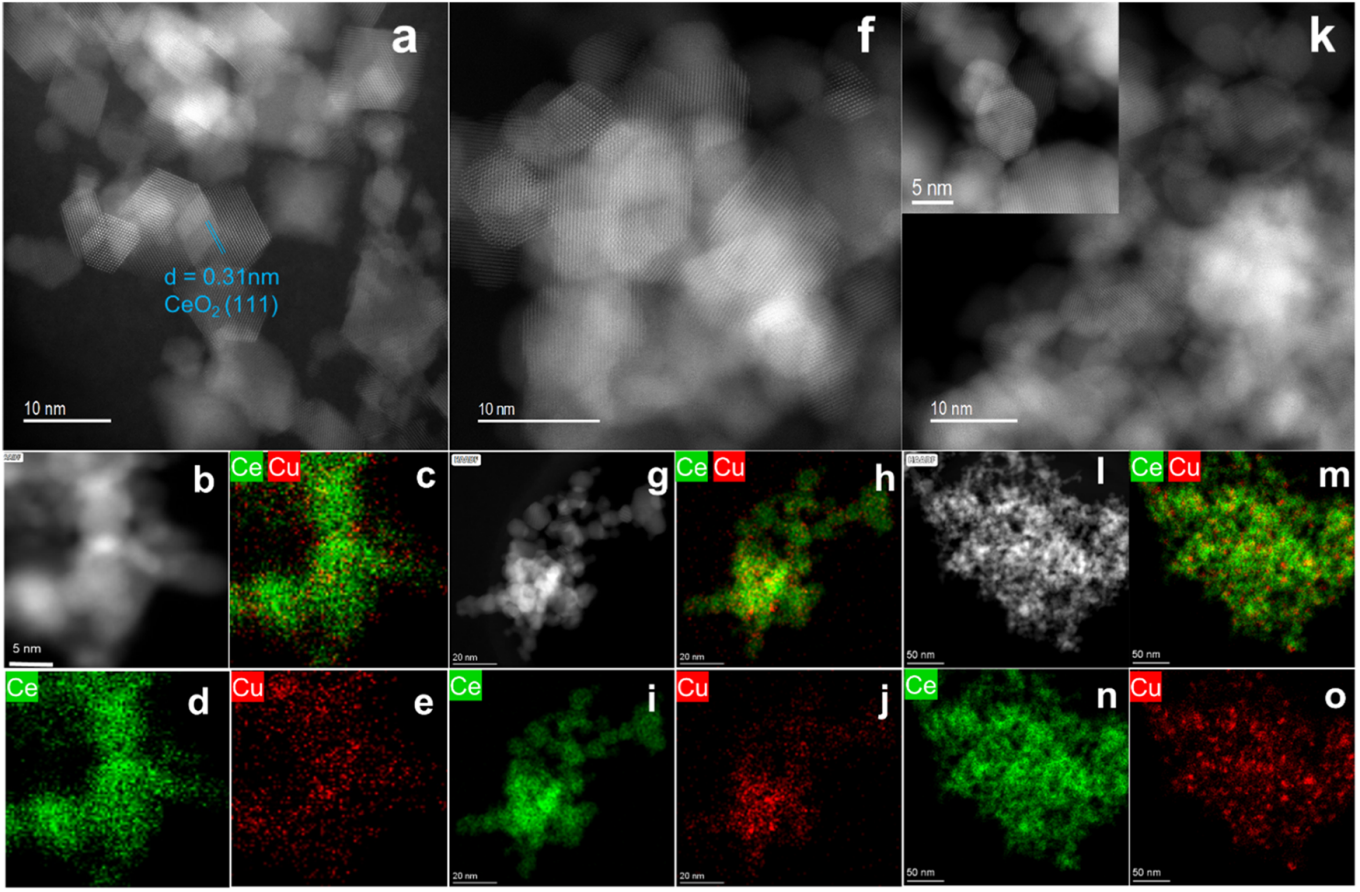
The crystal structure and morphology of CuCe-FSP samples. Scanning transmission electron microscopy (STEM) images and element mapping results for (a–e) 2.5CuCe-FSP, (f–j) 5CuCe-FSP, and (k–o) 10CuCe-FSP.

### Cu Binding Environment in Prepared and Reduced Samples

As reported by Biesinger et al.,^26^ XPS Cu 2p spectra can be deconvoluted to determine the Cu oxidation state in the samples when the information from shakeup satellite peaks and Cu Auger spectra is taken into account. The Cu 2p signal in Figure 3a shows that, different to the solution-derived 5CuCe-COP sample that is dominated by Cu^2+^ species (see Figure S13), calcined (unreduced) CuCe-FSP samples have a broad Cu 2p main peak that can be deconvoluted into Cu^2+^ at higher binding energy (BE) and Cu^0^/Cu^+^ at lower BE. Combined with Cu LMM spectra in Figure S14, these results show the presence of a significant fraction of Cu^+^ in CuCe-FSP samples in the order of 2.5CuCe-FSP > 5CuCe-FSP > 10CuCe-FSP. Additionally, Figure S15 shows that the CuCe-FSP samples contain a higher fraction of Ce^3+^ (16–26%) species compared to 5CuCe-COP (6%). The increased abundance of Cu^+^ and Ce^3+^ in the CuCe-FSP samples is likely associated with the formation of strongly bonded Cu-[O_*x*_]-Ce species at the surface and subsurface regions.^23^

**Figure 3:**
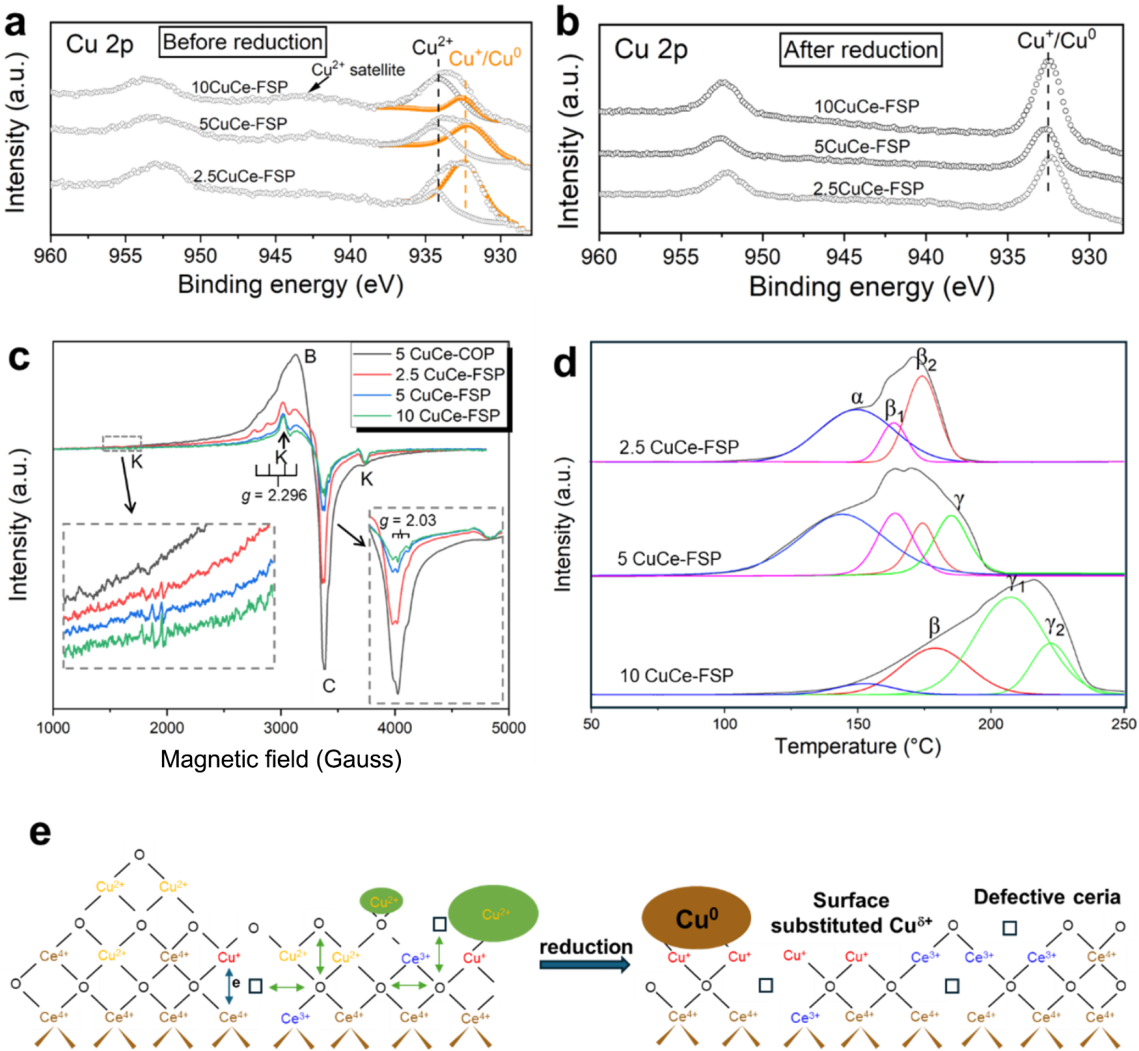
Electronic structure and redox behavior of Cu species in CuCe-FSP catalysts. XPS spectra of Cu 2p for (a) calcined and (b) reduced CuCe-FSP samples at 350 °C. (c) EPR spectra of as-prepared CuCe samples. (d) Temperature-programmed H_2_ reduction (H_2_-TPR) profiles of CuCe-FSP samples, three different regions (α, β, γ) were associated with three different copper species. (e) Schematic illustration of copper microstructure on the CeO_2_ surface before and after reduction. During reduction, the isolated Cu^2+^ ions/Cu^2+^ dimers (orange) and Cu^2+^ cluster/particles (green oval) were reduced to the surface-substituted Cu^δ+^ species (red) and metallic Cu^0^ (brown oval). Green and blue arrows indicate oxygen and electron exchange, respectively. Rectangle indicates oxygen vacancy.

Previous studies by Qiu et al.^23^ and Wang et al.^27^ have shown that high temperatures (700–800 °C) can drive Cu migration to the surface and subsurface of bulk Cu*_*y*_* Ce_1–_ *_*y*_* O_2–_ *_*x*_* solid solutions, leading to the formation of surface-substituted Cu*_*y*_* Ce_1–_ *_*y*_* O_2–_ *_*x*_* and highly dispersed CuO*_*x*_* species. Similarly, the high-temperature FSP synthesis is likely promoting the substitution of Ce ions with Cu ions on the surface ceria layer, which accounts for the observed high levels of Cu^+^ and Ce^3+^ species in the calcined CuCe-FSP catalysts. This defective surface Cu-doped ceria has been reported to produce abundant Cu^+^/Cu^2+^ and Ce^4+^/Ce^3+^ couples via electron exchange between copper and ceria cations and oxygen vacancy (O_v_)-mediated lattice oxygen mobility.^28^

EPR spectroscopy was used to further investigate the Cu speciation and its interaction with ceria. Figure 3c presents the X-band continuous wave (cw) EPR spectrum of the calcined CuCe sample acquired at room temperature over a broad magnetic field range. The characteristic oxygen vacancy signal (typically observed at *g* ≈ 2.0) is not evident, likely due to its occupation by hydroxyl and carbonate species based on the findings of Soria et al.^29^ Signal C, observed at a *g*-value of approximately 2.03, indicates the presence of isolated Cu^2+^ ions in ceria with a tetragonal distortion.^30,31^ Signal K, characterized by a well-resolved hyperfine splitting and a *g*-value of 2.296, corresponds to a Cu^2+^ dimers/pairs, in which two Cu^2+^ ions substitute two neighboring Ce^4+^ ions in the lattice with the smallest separation distance.^30,32^ Another broad and poorly resolved signal B centered at around *g* = 2.11 suggest that the corresponding ions are located in a Cu^2+^-containing aggregated phase (e.g., CuO clusters/particles).^33^ With higher intensity for signal B and C, calcined CuCe-COP exhibits a greater abundance of isolated and aggregated Cu^2+^ phase. In contrast, the FSP-derived samples show significantly lower intensities for these signals, consistent with their higher concentration of EPR-inactive Cu^+^ species. Additionally, signal K is notably more pronounced in the CuCe-FSP samples, suggesting a higher abundance of Cu^2+^ dimers. This observation aligns with the findings reported by Kydd et al.^34^

The deconvoluted H_2_-TPR peaks in Figure 3d were analyzed to assess the reducibility of the Cu–O and Cu–O–Ce species formed, which provides important insights into the binding environment of copper. Highly dispersed CuO_*x*_ clusters, which are weakly bound, typically reduce in the low-temperature range of 150–160 °C^35^ (denoted as α). This is followed by the reduction of surface-substituted Cu^δ+^–O–Ce species at 170–180 °C^24^ (denoted as β_1_ and β_2_), and finally, the reduction of CuO nanoparticles at higher *T* > 190 °C^27,36^ (denoted as γ_1_ and γ_2_). The splitting of the β and γ peaks is attributed to differences in coordination environment, oxidation state, and particle size.^37^ The results clearly show that 2.5CuCe-FSP is primarily composed of CuO_*x*_ clusters and Cu-substituted ceria, with a small portion of CuO nanoparticles. In contrast, 5CuCe-FSP exhibits a larger proportion of CuO nanoparticles, which becomes more significant for the 10CuCe-FSP sample.

In the XPS results of the reduced catalysts (Figure 3b), no satellite feature of Cu^2+^ was observed in the Cu 2p spectra. Further deconvolution of the XPS spectra (Figure S16) reveals that 5CuCe-COP contains 33.2% Cu^+^, attributed to the copper–ceria interface (Cu^+^–O_v_–Ce^3+^), as confirmed by Chen et al.^24^ Notably, a significantly higher fraction of Cu^+^ was found on the surface/subsurface of the CuCe-FSP samples, in the order of 2.5CuCe-FSP (80.5%) > 5CuCe-FSP (60.3%) > 10CuCe-FSP (41.5%), which correlate well with the amount of Cu^+^ sites calculated from chemical titration experiments in Table S3: 2.5CuCe-FSP (859 μmol/g) > 5CuCe-FSP (649 μmol/g) > 10CuCe-FSP (449 μmol/g) > 5CuCe-COP (272 μmol/g). In addition, both XPS and titration experiments confirm that the Cu^+^ content in 5CuCe-FSP is significantly higher than that in 5CuCe-COP. The significant fraction of Ce^3+^ (16–26%) observed in the calcined CuCe–FSP samples (Figure S15) confirms the formation of a defective structure during the FSP synthesis process. As expected, the XPS Ce 3d quantification (Figures S15 and S16) shows an increase in Ce^3+^ fractional concentration for all samples upon exposure to the H_2_ indicating further reduction of ceria.

### Proposed Atomic Structure of the CuCe-FSP Catalyst

Based on the results discussed above, we can conclude that the FSP technique produced a defect-rich ceria support that facilitates surface enrichment of copper through strong copper–ceria interactions. The Cu enrichment on the surface is supported by the higher Cu/Ce ratio observed in XPS compared to ICP results (Table S3). FSP offers extremely high flame temperatures and rapid quenching rates, which create highly nonequilibrium conditions. Under such conditions, the formation of well-dispersed ionic Cu species and surface-substituted Cu*_*y*_* Ce_1‑_ *_*y*_* O_2‑_ *_*x*_* domains are facilitated^34^ (Figure 3e (left)). These surface-bound ionic Cu species are present either as Cu^2+^ or Cu^+^, as a result of electronic exchange with the ceria lattice, as indicated by the arrows in Figure 3e. At the same time, the fast quenching kinetically traps oxygen vacancies within the ceria matrix, enhancing its defect density compared to slower synthesis routes.^15^ When the system reaches the limit of uniform surface dispersion of Cu (i.e., 10CuCe-FSP), discrete Cu oxide particle formation becomes favorable. There is no evidence for Cu incorporation into the bulk of ceria, as confirmed by XRD (Figure S7). Upon reduction, as shown in Figure 3e, copper atoms migrate and redisperse onto the ceria support, forming surface-substituted Cu-[O_*x*_]-Ce species with positively charged Cu exposed and Cu clusters/particles with Cu^0^ exposed. The exposed surface also includes surface oxygen vacancies and associated Ce^3+^ sites (see also Figure S36), as well as hydroxyl groups. This proposed structure will be re-examined in the following sections to establish a structure-performance relationship for the system. Next, we proceed with the mechanistic study of active sites and reactive intermediates.

### Surface Oxygen Vacancies as Active Sites

In situ Raman spectroscopy was used to study the evolution of defects under different reaction conditions. As shown in Figure 4a, the F_2g_ vibration mode of the CeO_2_ fluorite-type structure, observed at ∼454 cm^–1^, is accompanied by a broad D band. Upon reduction, the F_2g_ mode undergoes a red shift (red arrow), signifying the generation of additional defects in the ceria lattice. The D_1_ peak located at ∼543 cm^–1^ resulted from surface oxygen vacancies, where Ce^4+^ was reduced to Ce^3+^. The D_2_ peak at ∼603 cm^–1^ indicates defects within the ceria lattice.^38^ The D/F_2g_ ratio (D = D_1_ + D_2_) was used to characterize the concentration of defects in the sample (see Table S4). The intensity of D_2_ is relatively constant, and the change of D follows the trend of D_1_. The D_1_ peak was found to reversibly increase and decrease upon switching between H_2_ and CO_2_ + H_2_ atmospheres, confirming the role of surface oxygen vacancies as active sites and the dynamic nature of surface oxygen vacancies during the reaction. Furthermore, when switching to CO_2_ alone, a greater consumption of oxygen vacancies was observed, evidenced by a decrease in the D/F_2g_ ratio from 0.84 to 0.51 for 5CuCe-FSP. This suggests an enhanced CO_2_ activation on the catalyst surface.

**Figure 4:**
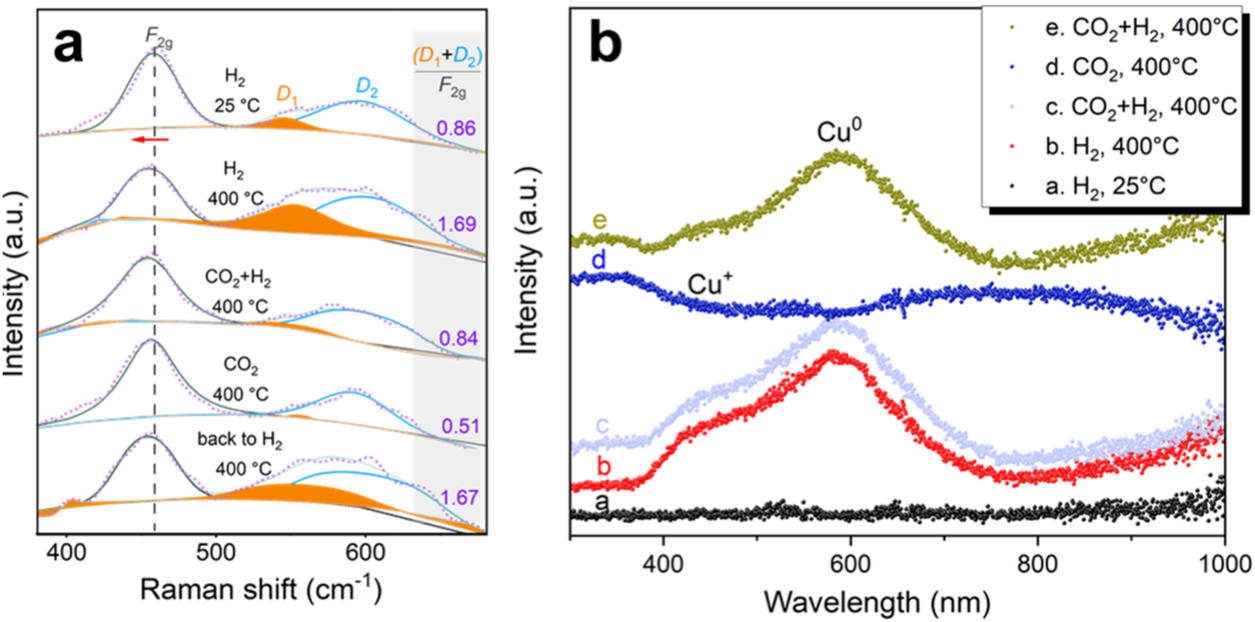
Dynamic evolution of surface chemical states under reaction conditions. (a) In situ Raman spectra of 5CuCe-FSP collected showing the evolution of surface oxygen defects (D1) under different conditions. (b) In situ UV–vis diffuse reflectance spectra showing the transitions between Cu^+^ and Cu^0^.

### Two Competing Surface Processes during rWGS Reaction

In situ UV–Vis spectroscopy coupled with mass spectrometry was employed to monitor the oxidation states of the Cu catalysts and the gaseous products during the rWGS reaction. From the UV–vis spectrum in Figure 4b, the Cu oxidation states can be derived for the 5CuCe-FSP catalyst under different reaction conditions, with the fresh catalyst under H_2_ flow at 25 °C used as the background (spectrum a). According to Bu et al.,^39^ metallic Cu is characterized by an absorption peak at around 580 nm, while Cu^+^ is indicated by an absorption feature between 400 and 500 nm. Under H_2_ flow at 400 °C (spectrum b in Figure 4b), reduced 5CuCe-FSP predominantly exhibits the optical extinction feature of Cu^0^, with a shoulder feature suggesting the coexistence of Cu^+^.^40^ When both CO_2_ and H_2_ are present (spectrum c), the Cu oxidation state remains relatively stable. Upon switching off H_2_ (Figure S17), a transition from Cu^0^ to Cu^+^ is observed, characterized by an initial surge in the Cu^+^ signal accompanied by a decline of the Cu^0^ within the first 15 s, followed by a slight reversal over the next 45 s before stabilizing into a Cu^+^-dominated feature at equilibrium (spectrum d). While CO_2_ dissociation to form O* and CO* (* indicates adsorbed species) was initially hypothesized as driving force behind the Cu state transition, this mechanism could be ruled out as CO production ceased when H_2_ was switched off, as shown in the MS spectra in Figure S17b. Interestingly, residual H* likely assists in reducing Cu^+^ (via the removal of surface oxygen species), indicated by the formation of H_2_O upon switching off the H_2_ dosage (black arrow in Figure S17b). The CO_2_-induced trend in Cu oxidation state was also observed for 5CuCe-COP, but not for the Cu-SiO_2_ sample (Figure S18). Therefore, it is tentatively postulated that increasingly accumulated species on ceria migrate and bond to copper surface, impacting the Cu oxidation state by adsorption-induced charge transfer.^41^ The coexistence of CO_2_ adsorption-induced Cu oxidation and H*-assisted Cu surface cleaning explains the trends in Cu oxidation state and species evolution observed in both the in situ UV–vis and MS spectra.

### Intermediates over 5CuCe-FSP Catalyst Surface

To probe the reaction mechanism, we first used diffuse reflectance infrared Fourier transform spectroscopy (DRIFTS) and studied the spectral features of 5CuCe-FSP at different reaction conditions. As shown in Figure 5a, the band at 3000–2650 cm^–1^ is assigned to the C–H bond of formate. Broad and poorly resolved bands in the region 1700–1200 cm^–1^ are mostly due to the O–C–O stretching of carboxylic (CO_2_^δ‑^/COOH*), formate (HCOO*) and carbonate (CO_3_^2–^) species, as described in detail in the literature.^42–44^ The 1215 cm^–1^ band is assigned to the bending mode of C–OH in bicarbonate^43^ and carboxyl (δ­(C–OH) of COOH*). Bands at around 990–1100 cm^–1^, and 850 cm^–1^ are related to the *v*_s_(C–O) and out-of-plane bending mode of δ­(O–C–O) of carbonate. At 50 °C, two signals are visible at 1605, 1285 cm^–1^ together with broad bands at 1650, 1215, and 1024 cm^–1^, which agrees well with the study of Vayssilov et al.,^43^ who have assigned the latter broad band to carbonate and bicarbonate species. The former signals at 1605 and 1285 cm^–1^ are associated with carboxylate (CO_2_^δ‑^),^13^ a known active intermediate in ceria-supported WGS catalyst,^44–46^ which is, however, less discussed in the rWGS reaction. The *v*_s_(C–H) mode at 2850 cm^–1^ started to evolve at 150 °C. Its frequency was coverage-dependent with a red shift upon decreasing surface coverage at higher temperatures of ≥250 °C,^47^ as shown in Figure S19a. This was also accompanied by the appearance of the combination modes (δ­(CH) + ν_s_(COO) = 2711 cm^–1^), (δ­(CH) + ν_as_(COO) = 2950 cm^–1^).^45,47^ The evolution of different species becomes obvious at 200 °C in the subtracted spectra in Figure 5a with a clear increase of formate species (2951, 2850, 2710 cm^–1^, and broad features at 1560–1610 and 1330–1380 cm^–1^) and carboxylic species (1285 cm^–1^), the diminishment of bicarbonate (1215, 1024 cm^–1^), and the appearance of a small carbonyl signal at 2110 cm^–1^ assigned to Cu^+^-CO^24^ (see also the Supporting Discussion S2 on CO-FTIR results). Carbonate was always abundantly preserved on the catalyst surface at the temperatures studied. At higher temperatures up to 400 °C, the concentration of surface species decreased significantly due to desorption and decomposition, with formate becoming the dominant species (Figure S20).

**Figure 5:**
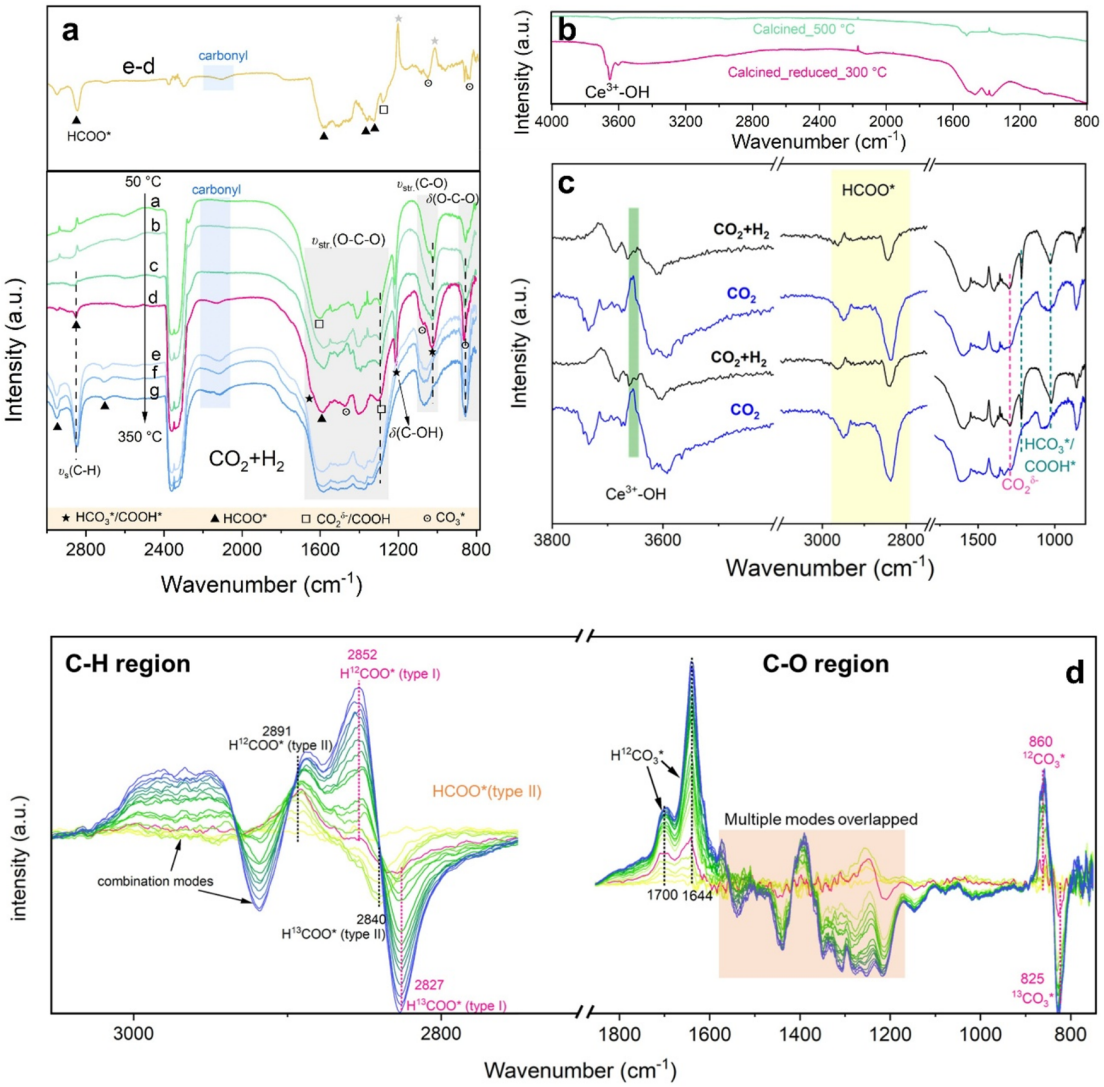
Identification and evolution of surface intermediates during rWGS reaction over 5CuCe-FSP. (a) in situ DRIFTS spectra of 5CuCe-FSP during the rWGS reaction at different temperatures. Spectra a-e (bottom) were recorded from 50 to 350 °C at 50 °C intervals. The spectra at the top were obtained by subtracting spectra d (200 °C) from e (250 °C), with spectra normalized to the intensity of the saturated CO_2_ peak. Reaction conditions: N_2_/CO_2_/H_2_ ratio = 2:1:3, total flow rate = 30 mL/min. (b) Spectra during pretreatment step (calcination and reduction) were recorded to monitor changes in hydroxyl group. (c) In situ DRIFTS spectra of 5CuCe-FSP during the rWGS reaction with H_2_ switched on or off at 200 °C to monitor changes in surface intermediates, including hydroxyl group, formate, (bi)­carbonate, and carboxylic species. (d) SSITKA-DRIFTS spectra (C–H and C–O vibrational region) were recorded for 5CuCe-FSP upon switching from ^12^CO_2_+H_2_ to ^13^CO_2_+H_2_ (1st to 20th min) at reaction equilibrium condition at 250 °C.

A distinct peak at 3652 cm^–1^ typical for Ce^3+^–OH is formed after catalyst reduction treatment (Figure 5b).^22,24^ Upon CO_2_ introduction at 200 °C (Figure 5c), a significant decrease in the Ce^3+^–OH signal is observed, indicating its consumption by CO_2_ and subsequent formate formation (2800–3000 cm^–1^). Under rWGS conditions (CO_2_ + H_2_), the Ce^3+^–OH signal recovered, while carboxylic species increased at the expense of formate. Upon switching back to CO_2_ and then CO_2_ + H_2_ again, the infrared spectrum reveals a reversible process between formate for CO_2_-only and carboxylic species for CO_2_ + H_2_. This conversion likely occurs via H* migration between C and O, a mechanism also proposed for the Pd-catalyzed rWGS by Nelson et al.^48^ Notably, the increased formate formation in CO_2_-only condition likely contributes to the Cu state change observed in the UV–vis spectra in Figure 4b. Additionally, significant formate accumulation was detected on the spent catalyst postreaction (after cooling to 30 °C in N_2_, Figure S19b). In addition, during the rWGS reaction, Ce^3+^–OH was consumed and further heating to 300 °C under N_2_ helps retain some of the −OH groups due to the migration of bulk hydride in ceria to the surface.^49^ In the presence of H_2_, bidentate formate can be converted via H*-assisted hydrogenation, rather than direct decomposition, which requires simultaneous cleavage of both the C–H and C–O bonds of HCOO*, a process with a high activation barrier of 2.21 eV reported in the literature.^50^ This conversion is accompanied by an increase in the Cu^+^-CO peak intensity.

### Cu-Promoted Carboxylate and Formate Formation

The DRIFTS spectra in Figure S21 show that at 50 °C, the ceria surface was dominated by bicarbonate species. At 250 °C, the formate peak on neat ceria is significantly weaker compared to 5CuCe-FSP due to its limited capability to activate H_2_. Unlike 5CuCe-FSP, no distinct carboxylic species were observed on neat ceria at this temperature. Interestingly, the nitric acid-leached catalyst (see also Supporting Discussion S3) demonstrates notable activity only at higher temperatures (Figure S22a), producing 3500 mmol CO/g_cat._/h compared to 5000 mmol CO/g_cat._/h for the unleached sample, whereas commercial ceria exhibits negligible activity. A higher activation energy barrier of 89.8 kJ/mol was calculated for the leached catalyst (Figure S22b). The TPR profile (Figure S24) further highlights the superior reducibility of FSP-derived ceria compared to commercial ceria, emphasizing its capability to create surface defects essential for CO_2_ and H_2_ activation. This nontrivial contribution of ceria is crucial for high-temperature rWGS (600 °C), while at lower temperatures, copper plays a key role in H* supply.

To investigate the nature of the surface-adsorbed species, SSITKA-DRIFTS was applied to track the change of species during ^12^C/^13^C isotopic exchange at reaction equilibrium.^51^ The depletion of the ^12^C species is observed as an upward peak, while the newly formed ^13^C species appears as a downward peak at a lower wavenumber, adjacent to the parent ^12^C species. As shown in Figure 5d, in the first 20 min of ^13^CO_2_ exposure at 250 °C, isotopic exchanges were detected for carbonate, formate, and bicarbonate, which confirmed the involvement of carbonate/bicarbonate and formates as active species that are effectively consumed and reproduced. Carbonate and bicarbonate are known to convert into formate under sufficient H* supply and elevated temperature,^52,53^ ultimately leading to CO production. However, overlapping features (e.g., bicarbonate, carbonate, formate, carboxylic species) made it challenging to distinguish the individual contributions and discern the isotopic exchange in the 1600–1200 cm^–1^ region. Specifically, the downward shift of the ^13^C bicarbonate feature compensates for the upward shift of the ^12^C carbonate feature, resulting in a relatively flat spectrum between 1600 and 1400 cm^–1^. The isotopic exchange of carboxylic species at 1300–1200 cm^–1^, if it occurred, would have been obscured by the broad carbonate feature.

### Different Types of Formate

In the C–H vibrational region, the isotopic exchange reveals the presence of two types of active formate. The isotopic exchange for the 2891/2840 cm^–1^ peak pair began within the first minute and completed within about 5 min. In contrast, the isotopic exchange associated with the 2852/2827 cm^–1^ peak pair and the broad feature for the combination mode started after 6 min and continued to grow over the next 14 min. These observations correlate well with the typical DRIFTS spectra for the 5CuCe-FSP sample (Figure S19a), where two distinct formate features at similar vibrational frequencies were observed. Monodentate formate on reduced ceria is thermally less stable and typically appears only at temperatures below 250 °C.^54^ The feature at 2850 cm^–1^ (along with the 2710, 1609, and 1388 cm^–1^ peaks) in the spectra of ceria is attributed to the C–H stretching mode of bidentate formate (type I) on ceria.^54^ The 2891 cm^–1^ band, barely observed on pure CeO_2_, is specifically assigned to formate located close to or on Cu (type II). This assignment is consistent with work by Mudiyanselage et al.,^54^ who associated the 2889 cm^–1^ and 1569 cm^–1^ features with formate in a tilted geometry on Cu^+^ on CeO_*x*_-Cu_*y*_O/Cu (111). Type II formate is less stable but more reactive than type I, as evidenced by its faster equilibration. This explains why isotopic exchange for type II formate occurred first, followed by type I formate.

### DFT Calculation for Carboxylate and Formate Pathways

Prior studies^50,55^ suggested that carboxyl (COOH*) species are less stable than formate (HCOO*) species at both the oxide-copper interface and on the copper surface, with a binding energy that is 0.5–1 eV lower. Our DFT calculations (Figure 6a,b, and Table S6) on ceria, Cu-substituted ceria, and ceria-supported Cu clusters confirm the higher stability of formate compared to carboxylate, which is reflected by a more negative free energy of formate. The reaction energy (Δ*E*_rxn_) for carboxylate (CO_2_^δ‑^) conversion to carboxyl (COOH*) is as low as 0.13 eV for Cu-substituted ceria and 0.22 eV for ceria-supported Cu. Moreover, its subsequent decomposition to CO* and OH* is exothermic (negative Δ*E*_rxn_).

**Figure 6:**
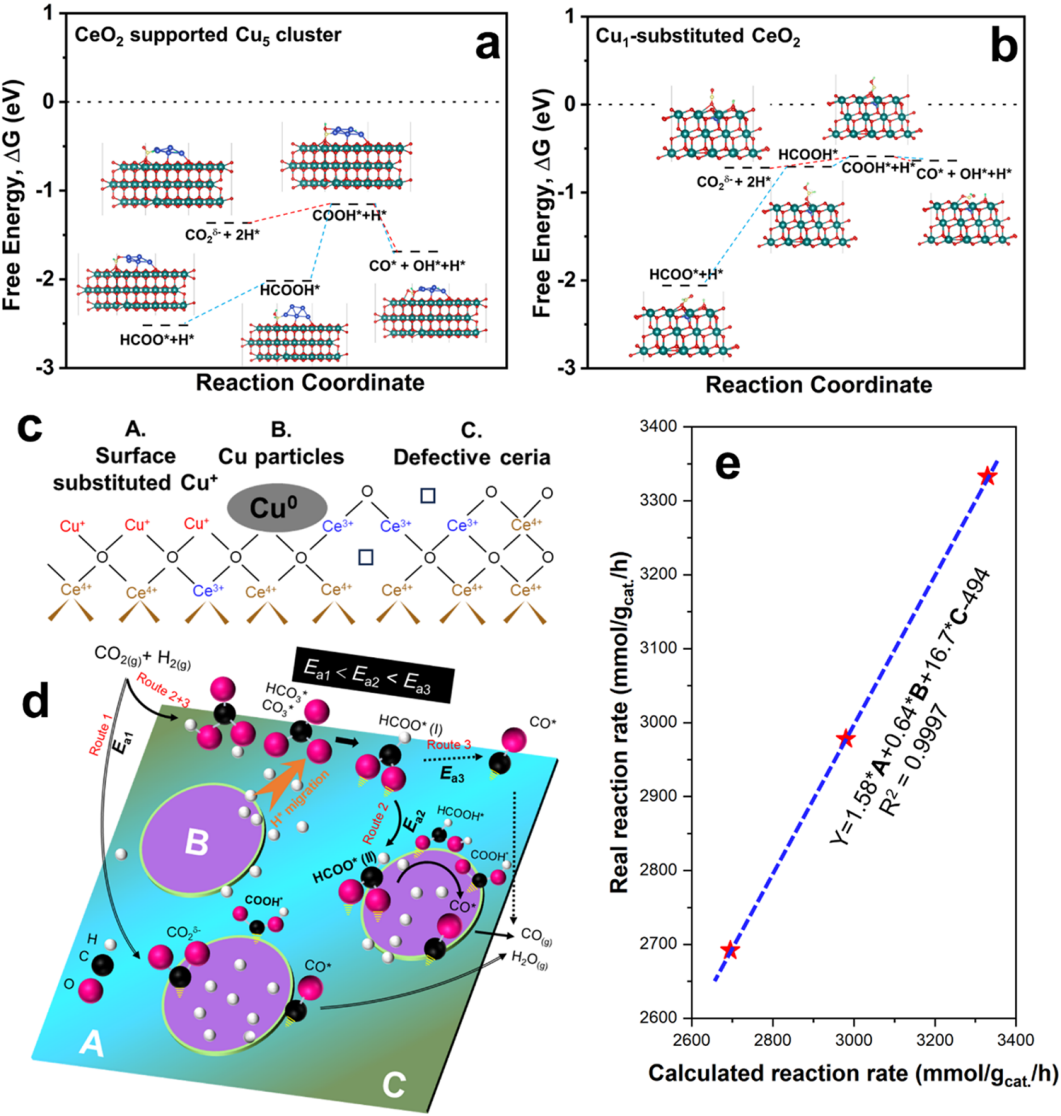
Mechanistic and structure–performance insights into CuCe-FSP catalysts for rWGS. DFT-calculated energy profiles for the carboxylate- (red dotted line) and formate-mediated (blue dotted lines) reaction pathways over (a) Cu_5_ cluster supported on CeO_2_ (with O_v_) and (b) Cu_1_-substituted CeO_2_ (with O_v_). (c) Schematic of three distinct active surface structures (A: Cu^+^ sites, B: Cu^0^ sites, C: ceria defect sites) on CuCe-FSP catalysts. (d) Proposed reaction network involving three parallel pathways occurring at these distinct active sites, including key surface intermediates. (e) Structure–performance correlation for the CuCe-FSP system. See details of calculated reaction rate in the Experimental method section. Experimentally observed rWGS rates (tested at 600 °C, GHSV = 693,600 mL/g/h) correlate linearly with the fitted reaction rates derived from the combined concentrations of active sites A, B, and C across different samples (reaction rate: 5CuCe-FSP > 10CuCe-FSP > 2.5CuCe-FSP).

On the other hand, despite being more stable than carboxyl species and widely observed, the role and evolution of formate in the rWGS reaction is not well understood. The direct decomposition of formate is kinetically unfavorable, which makes a hydrogenation step more likely.^50^ According to Zhao et al.^50^ and Grabow et al.,^56^ the conversion of HCOO* to HCOOH* is both kinetically more favorable and less endothermic than the HCOO* to H_2_COO* route. Furthermore, Yoo et al.^57^ proposed that the decomposition of HCOOH* to CO* and OH* (via COOH*) on Cu surfaces occurs with a low activation energy of 0.41–0.47 eV (42.5 ± 3 kJ/mol), making it a plausible reaction pathway. Our DFT results further reveal that the hydrogenation of HCOO* to HCOOH*—known as the rate-determining step of the formate pathway in methanol synthesis^13^—is associated with a Δ*E*_rxn_ ranging from 0.5 to 1.32 eV.

These results confirm that the carboxylate pathway is less energy-demanding than the formate pathway, aligning with the DFT studies on the rWGS pathway over copper–ceria structures by Graciani et al.^13^ and Liu et al.^6^ Notably, among three calculated structures, the Cu-substituted structures favor the carboxylate pathway, as indicated by the less stable carboxylate species and a lower Δ*E*_rxn_ for COOH* formation. Additionally, Cu facilitates H* supply and promotes the formation and hydrogenation of carboxylate and formate, as evidenced in spectra of Figures S21 and S22 (see also Supporting Discussion S3).

### Three Parallel Reaction Pathways

A summary of the proposed rWGS reaction network, derived from experimental observations and DFT calculations, is shown in Figure 6d. We propose that three parallel pathways—involving either carboxylate or formate—collectively contribute to the rWGS rate over three distinct active surface structures (Figure 6c) of CuCe-FSP catalysts. The formate-mediated pathway plays a limited role in the overall rWGS rate due to the high thermal stability of formate and high Δ*E*_rxn_ required for its hydrogenation. In contrast, the carboxylate pathway, once formed (as observed in the CuCe-FSP sample), is thermodynamically more favorable. Its hydrogenation to carboxyl (COOH*) and subsequent dissociation (route 1 in Figure 6d) occurs with a lower activation energy (*E*_a1_), making it dominant at lower temperatures. Additionally, due to the high surface coverage of formate species at temperatures <400 °C, formate can rearrange into COOH*, further supporting the carboxylate pathway. As the temperature increases above 400 °C, where H_2_ activation improves, formate/H* migration (indicated by the orange arrow in Figure 6d) and H-assisted formate hydrogenation at the copper–ceria interface (route 2, *E*_a2_) become significant contributors to CO production, which dominates in the 5CuCe-COP sample (Figure S5a). At even higher temperatures (>500 °C), the dissociative adsorption of H_2_ on defective ceria is significantly enhanced,^58^ leading to substantial CO production via the hydrogenation of HCOO* (route 3, *E*_a3_) at active sites such as Frustrated Lewis pairs (FLPs).^22^ The associated activation energies follow the trend: *E*_**a3**_ **>** *E*_**a2**_ **>** *E*_**a1**_. Notably, rWGS reaction over CuCe-FSP catalyst—characterized by abundant surface-substituted Cu^+^ structures—is primarily governed by route 1, as evidenced by its significantly lower apparent activation energy compared to other studied samples (89.8 kJ/mol for leached ceria (Figure S22) > 50.41 kJ/mol for 5CuCe-COP > 21 kJ/mol for 5CuCe-FSP) and reported literature values (Figure 1b).

### Structure–Performance Relations

Previous reports on CO_2_ hydrogenation to methanol have shown a strong correlation between the methanol formation rate and the abundance of interfacial structures, particularly the interfacial length^11^ of Cu^0^-dominated CuCe catalysts, and the amount of defects in oxides (e.g., terminal hydroxyl groups^59^ on Cu/Al_2_O_3_ for methanol synthesis). However, no similar correlation has been found so far for rWGS catalysts. As discussed before and illustrated in Figure 6c, the catalyst reduction results in three distinct surface domains: surface-substituted Cu^+^ on ceria (site A), Cu^0^-dominated copper clusters/particles (site B) and CeO_2_ surface defects for initial CO_2_ chemisorption (site C). These active sites were quantitatively analyzed using N_2_O, CO, and CO_2_ titration experiments (see experimental methods). Surface Cu^0^ is known to be responsible for H_2_ activation, while Cu^+^ species are associated with Cu-substituted ceria structure and the copper–ceria interfacial perimeter. Cu-substituted ceria plays a key role for CO_2_ activation and hydrogenation along the carboxylate pathway, particularly via the formation of carboxylic species. This is further supported by DRIFTS data in Figure S25, which show a higher abundance of carboxylic species on Cu^+^-rich 5CuCe-FSP compared to Cu^0^-dominated 5CuCe-COP, leading to a more reactive catalyst surface for CO_2_ dissociation to CO between 350 and 750 °C (as shown in the CO_2_-TPD results in Figure S26). Moreover, the 5CuCe-FSP catalyst is less dependent on the H_2_ partial pressure (i.e., lower H_2_ reaction orders, Table S5) compared to the 5CuCe-COP catalyst, indicating a higher coverage of H* on 5CuCe-FSP. Finally, the CO production rates at 600 °C for CuCe-FSP catalysts does not follow the trend of exposed Cu^+^ or Cu^0^ sites alone (Table S3), suggesting the involvement of multiple reaction sites in CO formation. A good correlation is observed only when combining the contribution of exposed Cu^0^, Cu^+^, and CO_2_ adsorption sites (Figure 6e), supporting the hypothesis that catalytic activity depends on all three active sites.

## Conclusions

In this study, we investigated the catalytic performance and reaction mechanisms of CuCe catalysts in the rWGS reaction, with particular emphasis on the role of surface oxygen vacancies, Cu oxidation states, and formate/carboxylate intermediates. The FSP-derived CuCe catalysts exhibit outstanding catalytic activity (8094 mmol/g_cat._/h) and stability with significantly lower apparent activation energy barrier compared to previously known rWGS catalysts. Our findings highlight the structural superiority of the FSP-derived CuCe structure, featured by a highly defective ceria support and Cu-substituted ceria that play a central role in the formation and transportation of reactive intermediates. The surface defects on the ceria support, particularly oxygen defects and hydroxyl groups, play a key role for the initial CO_2_ activation. In situ Raman and UV–vis spectroscopy revealed the dynamic evolution of surface states under varying reaction conditions, highlighting the sensitivity of the catalyst to the reaction gases (i.e., strong adsorbate–catalyst interactions) and the coexistence of complex surface processes (e.g., H* and HCOO* migration). DRIFTS experiments distinguished two types of formate species, with bidentate formate on ceria migrating to Cu sites for completing the reaction, demonstrating a cooperative mechanism between copper and ceria. Unlike the traditionally proposed formate-dominated pathway, our spectroscopic results and computational calculation confirm the significant role of carboxylic species on FSP-made copper–ceria catalyst for delivering excellent rWGS performance. The exceptionally low activation energy (*E*_a_) is attributed to the carboxylate-mediated pathway, while the record-high catalytic activity is linked to the synergy of three reaction pathways over distinct active sites. At lower temperatures, the carboxylate route dominates, whereas at higher temperatures H-assisted formate hydrogenation—occurring both on ceria surface and at the interface—becomes predominant. These findings are a step toward a more comprehensive understanding of the complex structure–activity relationships in CuCe catalysts and provide valuable insights for the rational design of efficient catalysts for CO_2_ hydrogenation reactions.

## Supplementary Material



## Notes

Experimental details for the synthesis of catalysts, catalyst characterizations (STEM, EPR, XRD, XPS, H_2_-TPR, and titration of surface sites), in situ DRIFTS/Raman/UV–vis studies, catalysis experiment, reactor schematic, DFT calculations and comparative (PDF)
