# Supplementary material for: Flame-Made Surface-Substituted Copper–Ceria as an Excellent Reverse Water–Gas Shift Reaction Catalyst via Three Reaction Pathways
Source: J Am Chem Soc. 2025 Sep 1;147(36):32650–62. doi: 10.1021/jacs.5c07701 (PMC12426932; doi:10.1021/jacs.5c07701)
Supplement: Supplementary file 1 [file ja5c07701_si_001.pdf]

Supporting information

Flame-Made Surface-Substituted Copper–Ceria as an Excellent Reverse Water–Gas Shift Reaction Catalyst via Three Reaction Pathways

Bingqiao Xie*[a,b], Yi Fen Zhu[b], Mahdi Shakeri[a], Seongmin Jin[a], George O'Connell[b], Sankhadip Saha[b], Mounir Mensi[a], Priyank V. Kumar[b], Jeremy S. Luterbacher[a], Emma C. Lovell[b], Rose Amal[b], Oliver Kröcher*[a,c]

[a]Institute of Chemical Sciences and Engineering (ISIC), École Polytechnique Fédérale de Lausanne (EPFL), Lausanne, 1015 Switzerland

[b]School of Chemical Engineering, UNSW, Kensington, NSW, 2052 Australia

[c]Paul Scherrer Institute (PSI), PSI Center for Energy and Environmental Sciences, CH-5232 Villigen, Switzerland

*Correspondence to Bingqiao Xie (bingqiao.xie@epfl.ch) and Oliver Kröcher (oliver.kroecher@psi.ch)

Experimental method

Catalyst synthesis

Three copper-ceria catalysts (denoted as (*x*)CuCe-FSP) with Cu loadings of *x* = 2.5, 5.0, and 10 wt% were synthesized via flame spray pyrolysis (FSP) (**Figure S1**). Copper(II) 2-ethylhexanoate (Sigma Aldrich) and cerium 2-ethylhexanoate (Fisher Scientific, 12% Ce in 49% 2-ethylhexanoic acid) served as precursors for the copper-ceria nanoparticles. The precursors were dissolved in xylene (Sigma Aldrich, reagent grade) at 60 °C with the aid of sonication, maintaining a constant Ce concentration of 0.5 M. The precursor solution was fed into the flame at 5 mL/min and dispersed using 5 L/min of O_2 (Coregas, > 99.95%). The flame was supported by a secondary flame fed with a mixture of O_2 (3.2 L/min) and CH_4 (0.6 L/min, Coregas, > 99.95%), while 5 L/min of sheath O_2 directed the nanoparticles toward a filter paper coupled with a vacuum pump for recovery. The as-prepared particles were collected by scraping them from the glass fibre filter paper positioned at a vertical distance of 73.5 cm from the flame outlet. This setup allows for effective particle collection while minimizing thermal damage to the filter. The resulting particles were calcined at 550 °C for 3 h to remove residual organic species.

To examine the role of Cu in the CO_2 hydrogenation reaction, Cu was selectively leached using nitric acid. For this, 400 mg of the 5 wt% CuCe-FSP sample was immersed in 20 mL of ~1.5 M nitric acid at room temperature for 3 h. The mixture was then diluted with deionized (DI) water and washed repeatedly until the filtrate reached a neutral pH. The residue was dried overnight at 110 °C. As shown in **Figure S23**, Cu was effectively removed with minimal alteration to the CeO_2 support (see also **Supplementary discussion S3**).

For the co-precipitated copper-ceria catalyst (5CuCe-COP), we followed a modified protocol based on our previous work[1]. Specifically, appropriate amounts of $Cu(NO_3)_2$ and $Ce(NO_3)_3$ were dissolved in 20 mL of deionized water (DI water) to achieve a nominal Cu loading of 6.2 wt.%, matching the actual Cu content in 5CuCe-FSP, as determined by ICP elemental analysis. Separately, 2.018 g of $NaHCO_3$ was dissolved in 40 mL of DI water to prepare the precipitating solution. The metal nitrate solution was heated to 45 °C, and the $NaHCO_3$ solution was slowly injected over 10 min using a syringe. The mixture was stirred at 600 rpm and maintained at 65 °C for 90 min. The resulting precipitate was vacuum-filtered and washed thoroughly with DI water until the filtrate reached a neutral pH. The residue was dried overnight at 110 °C, followed by calcination in air at 550 °C for 3 h, with a ramp rate of 10 °C/min.

Catalyst characterisations

(Electron paramagnetic resonance) EPR spectra were recorded at room temperature using a Bruker EMX spectrometer operating at a microwave frequency of 9.6 GHz (X-band). The magnetic field was modulated at 100 kHz, with a field modulation amplitude of 0.4 mT and an incident microwave power of 20 mW. The *g* values were calculated using precise measurements of the frequency and magnetic field. The composition of metals in the catalyst was quantified by inductively coupled plasma mass spectrometry (ICP-MS, NexION 350D, Perkin Elmer). XRD measurements were conducted using a Bruker D8 Advance diffractometer. The instrument was equipped with a copper anode (Cu $K\alpha$ radiation, λ = 0.154 nm) and a LYNXEYE XE detector. The XRD patterns were recorded in a 2θ range from 10° to 80° with a step size of 0.02 and a count time of 1 s per step. The tube voltage and current were set to 40 kV and 40 mA, respectively. The data obtained were also used to calculate the mean crystallite size of the catalysts using the Scherrer equation. X-ray photoelectron spectroscopy (XPS) analysis was performed using a PHOIBOS 150 NAP (SPECS GmbH) with monochromated Al Kα radiation as the

excitation source. Reduced samples were prepared by reducing under H_2/He mixture (H_2 = 5 mL/min, He = 20 mL/min) at 350 °C (10 °C/min ramping) for 1 h in a commercial Harrick reactor. After cooling, the reactor was enclosed and the sample was transferred to the XPS sample holder (sample powder pressed onto the indium foil) inside the glovebox, ensuring that it was not exposed to air during the transfer process. Aberration-corrected (AC) high-angle annular dark-field scanning transmission electron microscopy (HAADF-STEM) and associated EDX elemental mapping were obtained using a Thermo Fisher Scientific Titan Themis operated at 300 kV, equipped with a CEOS DCOR probe corrector and a Super-X 4-quadrant detector for EDX. The samples were dispersed into ethanol solution, sonicated, dropped onto a lacey carbon gold grid, and dried inside a heated vacuum oven (80 °C, overnight).

Temperature-programmed reduction with H_2 (H_2-TPR) was conducted with an Autochem II setup (Micromeritics). Approximately 50 mg of catalyst were loaded into a U-shaped quartz reactor and pre-treated under Ar flow at 350 °C to remove pre-adsorbed water. After cooling to room temperature, the catalyst was reduced by heating from room temperature to 900 °C at a heating rate of 10 °C min^{-1} under 10% H_2 with the balance being Argon, during which H_2-TPR was recorded.

Titration of different surface sites

Three types of surface sites – metallic copper, positively charged copper (Cu^+), and CO_2 adsorption sites – were characterized using the methods described below:

The exposed metallic copper sites were quantified using a methodology adapted from Zabilskiy et al.[2, 3]. To selectively titrate metallic copper surface sites while avoiding contributions from ceria oxygen defect sites and over-oxidation of copper, N_2O pulsing (0.48 mL of 2% N_2O/He) was performed at 35 °C. For each experiment, approximately 50 mg of the sample were loaded into a U-shaped quartz tube and pre-reduced in a 10% H_2/Ar mixture at 250 °C (ramp rate: 10 °C/min) for 1 h. The system was then flushed with Ar to remove adsorbed water before cooling to 35 °C. Once the temperature stabilized and the TCD signal returned to baseline, pulses of 2% N_2O in He were injected using a 0.48 mL sample loop until no further N_2O consumption was detected. The decomposition of N_2O and formation of N_2 were monitored using coupled MS spectrometer. The total amount of consumed N_2O was calculated by comparing the signals from saturated and reactive pulses in the MS spectrometer. No significant N_2 formation is detected, indicating negligible N_2O decomposition on bare ceria (**Figure S3**). After metallic copper was oxidized to Cu^+ by N_2O, the total number of exposed Cu^+ sites – including those present initially in the reduced catalyst and those formed during N_2O oxidation – was measured via CO titration, following the method of Koryabkina et al.[4]. After completing the N_2O titration experiment, the system was flushed with Ar, and the temperature was reduced to 30 °C. Pulses of 5% CO in Ar (0.48 mL) were injected into the sample tube using a 0.48 mL sample loop until no further CO consumption was observed. The CO uptake was calculated based on the MS-measured CO signal. CO adsorption was attributed exclusively to Cu^+ sites, assuming a 1:1 stoichiometry (CO:Cu^+). The number of surface Cu^+ sites (N) was calculated as: $N = \frac{a}{22.4 \times m}$, where a is the amount of CO adsorbed (in uL), and m is the mass of the catalyst (g). Since CO_2 interacts primarily with the ceria surface, CO_2 pulse chemisorption (10% CO_2 with 0.48 mL sample loop) was conducted to determine the CO_2 adsorption capacity of the catalyst. These experiments provided data to calculate the concentration of CO_2 adsorption sites, including mostly oxygen vacancies, and hydroxyl groups—both critical for initial CO_2 activation (e.g., formation of carbonate/bicarbonate species as seen in DRIFTS).

In-situ Raman experiments

In situ Raman spectroscopy (**Figure S2**) was performed using a Renishaw inVia Raman microscope equipped with a 405 nm laser and a high-temperature reaction cell to investigate changes in the defective structure of ceria under reaction conditions. The system employed a 3000 lines/mm grating and a Renishaw CCD detector. Spectra were acquired using 10% laser power, a 3-second exposure time per scan, and 40 accumulations to ensure high-quality data. The spectral range was set between 66.14 cm^{-1} and 1843.04 cm^{-1}. The catalysts were prepared in the form of pellets by applying a pressure of 3 bars. These pellets were placed inside a reaction cell (LINKAM THMS600) connected to a gas flow of CO_2 and H_2 (20% H_2 in N_2). Initially, the catalysts were held at room temperature, then heated to 400°C under a 50 mL/min flow of 20% H_2 and kept at this temperature for 30 min for catalyst reduction. Subsequently, 5 mL/min CO_2 gas was introduced (CO_2+H_2), followed by termination of the hydrogen flow (CO_2-only). Then, it was switched back to CO_2+H_2, followed by H_2-only. The gas composition was switched only after the Raman spectrum stabilized under the current condition. This approach allowed for a detailed examination of the surface interaction and the reversibility of structural changes under various reactant conditions.

In-situ UV-Vis experiments

The evolution of Cu oxidation states was investigated using diffuse reflectance UV-visible (DRUV-Vis) spectroscopy with a Harrick reactor cell sealed by a calcium fluoride window. Spectra were acquired using a fiber optic spectrometer (AvaSpec ULS2048CL, Avantes) coupled with a deuterium halogen light source (Avalight-DHS, Avantes) and a reaction probe (FCR-7UVIR600, Avantes) positioned perpendicular to the calcium fluoride window. Approximately 50 mg of the sample were loaded into the reactor cell, and the temperature was ramped to 400 °C under an Ar flow of 40 mL/min. At this temperature, background and reference spectra were recorded. The sample was subsequently reduced under an H_2 flow of 6.6 mL/min for 30 min. During typical reaction conditions (CO_2:H_2 = 1:2, total flow rate = 20 mL/min), UV-Vis absorption spectra were recorded over the wavelength range of 300–1000 nm. To study the evolution of surface states under various conditions, the gas composition was purposely altered. The effluent gas composition was analyzed using mass spectrometry (MS) to correlate surface changes with reaction products.

In-situ DRIFTS experiments

In-situ diffuse reflectance infrared Fourier transform spectroscopy (DRIFTS) was performed using a Brucker VERTEX 70v FTIR spectrometer with a N_2-cooled mid-infrared (MIR) source equipped with KBr optics. Details on the DRIFTS set-up are provided in a prior study[5]. The Cu-containing samples were diluted with KBr (sample-to-KBr ratio of 1:9) to provide a sufficient reflectance signal amplitude (neat CeO_2 was tested without dilution). Around 50 mg of (diluted) samples were charged into the Harrick cell and calcined at 500 °C under synthetic air for 1 h (20 mL/min), followed by reduction at 400 °C in an N_2:H_2 mix (ratio = 2:1, total flowrate = 30 mL/min) for 1 h. Spectra were recorded (KBr spectra as background) at this stage to monitor the change of hydroxy groups. After cooling to 50 °C under He (flowrate = 70 mL/min), background spectra were collected. Under typical reaction conditions (N_2:CO_2:H_2 = 2:1:3, total flowrate = 30 mL/min or 12 mL/min for reserving more surface species), transmission spectra were collected at each temperature (typically 50–400 °C with 50 °C intervals). Each spectrum was collected from 4000 cm^{-1} to 600 cm^{-1} (64 scans, resolution of 2 cm^{-1}). The gas composition (i.e., CO_2 and H_2) was altered purposely to study the evolution of surface species at

different conditions. After reaction, the system was cooled down to 30 °C, followed by a temperature increase to 300 °C under N_2 flow, and finally in a N_2:H_2 mix (ratio = 2:1, total flow rate: 30 mL/min). The spectra were recorded at each step to track changes of the surface species. CO-FTIR experiments were also conducted to help identify the surface copper species (see **Supplementary discussion S2** on the CO-FTIR).

For steady-state isotopic kinetic analysis (SSITKA), DRIFTS samples after pretreatment were pre-purged with a mixture of N_2:$^{12}CO_2$:H_2 = 2:1:3, total flow rate: 12 mL/min for 30 min at 50 °C, followed by a temperature increase to 250 °C. When the DRIFTS spectra had stabilized, DRIFTS spectra were collected and used as the background for subsequent scans. The $^{12}CO_2$ gas was then switched to $^{13}CO_2$ while maintaining the same H_2 gas flow. Differential DRIFTS spectra, with H_2:$^{12}CO_2$-saturated spectra as the background, were collected at 1-min intervals for 20 min.

Catalytic activity tests

The performance of the prepared copper-based catalysts in the rWGS reaction was evaluated using a custom-built quartz fixed-bed reactor (inner diameter: 0.6 cm, length: 28 cm) operated at atmospheric pressure and temperatures between 400–600 °C. A thermocouple (TC) inserted into the catalyst bed monitored the temperature, while an electrical heating jacket provided uniform heating. Gas flow rates of N_2, CO_2, and H_2 were controlled using electronic mass flow controllers (Bronkhorst EL-flow). Effluent gas concentrations were continuously analyzed with FT-IR gas analyzers (MATRIX II-MG01) equipped with OPUS-GA software and a 10 cm heated gas cell (120 °C).

Before testing, ~10 mg of catalyst were diluted with inert silicon dioxide granules (100 mg). Catalyst dilution help spread the heat generated from reaction (endothermic) and reduce the intensity of the reaction per unit volume, thus reduced hot spots and local temperature gradient. For activity evaluation, catalyst was reduced at 400 °C in a flow of 28 mL/min H_2 and 35 mL/min N_2 (heating rate: 10 °C/min) for 1 h. The desired CO_2:H_2 molar ratio diluted in N_2 was then dosed, and the rWGS activity was measured between 400 °C and 600 °C in 50 °C intervals once the FT-IR signals stabilized. Unless otherwise noted, a CO_2/H_2 ratio of 1/3 and a gas hourly space velocity (GHSV) of 278,400 mL/$g_{cat.}$/h (6.6 mL/min CO_2, 19.8 mL/min H_2, and 10 mL/min N_2) were used.

The stability of the catalysts was tested at 600 °C with a GHSV of 376,800 mL/$g_{cat.}$/h. The activity of 5CuCe-FSP at 600 °C was also tested at different GHSVs (487,500 to 3,060,000 mL/$g_{cat.}$/h) with a fixed H_2/CO_2 ratio of 3 in 4 mL/min N_2 using a reduced catalyst loading (4 mg catalyst diluted with 100 mg silica). The CO_2 conversion (X_{CO_2}), CO selectivity (S_{CO}), and production rate (r; in mmol/$g_{cat.}$/h) were calculated using the equations below:

$$X_{CO_2}(\%) = \frac{n_{CO_2}^{in} - n_{CO_2}^{out}}{n_{CO_2}^{in}} \times 100$$

$$S_{CO}(\%) = \frac{n_{CO}^{out}}{n_{CO}^{out} + n_{CH_4}^{out}} \times 100$$

$$r = \frac{F \times X_{CO_2}}{W} \times \frac{60 \times 1000}{22.4} = \frac{F \times X_{CO_2}}{W} \times 2678.57\ (mmol/g/h)$$

Where $n_{CO_2}^{in}$ is the molecular fraction of CO_2 in the feed gas and $n_{CO_2}^{out}, n_{CO}^{out}, n_{CH_4}^{out}$ are the molecular fractions of CO_2, CO, CH_4 at the reactor outlet, respectively. No methane was detected over all the studied catalysts. *F* is the CO_2 flow rate (mL/min), and *W* the catalyst weight (mg).

The calculated reaction rate shown in **Figure 6e** was derived from a linear combination model based on the concentrations of three experimentally identified active sites: A: Cu^+ surface-substituted sites ($CuyCe_{1-y}O_{2-x}$), B: Metallic Cu^0 sites, C: Oxygen-deficient ceria (CeO_{2-x}) sites. The fitting was performed using the following empirical equation:

$$\text{Calculated Rate (Y)} = a \cdot [A] + b \cdot [B] + c \cdot [C]$$

Where Y is the predicted reaction rate (mmol/$g_{cat.}$/h), [A], [B], and [C] represent the amount of each active site (value taken from **Table S3**). a, b, and c are fitting coefficients corresponding to the site-specific intrinsic activity. The coefficients a, b, and c were then determined by fitting the model to the experimentally measured reaction rates of the three CuCe-FSP catalysts (2.5CuCe-FSP, 5CuCe-FSP, and 10CuCe-FSP) using the "Multiple Linear Regression" tool of Origin software.

For the kinetic analysis, the CO_2 conversion was kept below 20% by adjusting the temperature and gas flow rate to eliminate gas diffusion limitation. Flame-made catalysts are composed of finely dispersed metal species on a nonporous support with high external surface area, offering excellent thermal stability and favorable mass transfer characteristics.[6-8] Their nonporous nature[6-8] minimizes internal diffusion limitations. The reaction orders of CO_2 and H_2 over 5CuCe-FSP and 5CuCe-COP were obtained by adjusting the CO_2 and H_2 partial pressures at 400 °C and 500 °C.

To examine whether significant external mass diffusion limitations exist, we followed the method reported by Perego et al. [9], which involves varying the catalyst amount while maintaining constant contact time. This was achieved by simultaneously adjusting the flow rate and catalyst mass to keep the Gas Hourly Space Velocity (GHSV) constant. As shown in **Figure S4**, when the catalyst loading was increased from 10 to 20 and 30 mg, the CO_2 conversion remained relatively constant—30.6–31.9% at a H_2/CO_2 ratio of 3, and 20.7–21.5% at a ratio of 1. These variations fall within the experimental error (±2% conversion), as verified through repeated measurements. This confirms that no significant external mass transfer limitations are present under the conditions used in this study.

Due to their low activity, commercial ceria and nitric acid-leached samples were tested separately. Approximately 200 mg of commercial ceria or 150 mg of leached samples were calcined at 500 °C for 2 h under N_2 (30 mL/min), cooled to 120 °C, and reduced at 600 °C (ramp rate: 10 °C/min) in 50% H_2 diluted with N_2 (total flow rate: 20 mL/min). The H_2O signal was monitored using FT-IR gas analysis during reduction. For leached samples, the activity was also tested at different GHSVs at 600 °C.

Equilibrium conversion calculation

The enthalpy (*H*) and entropy (*S*) of reactants and products at different temperatures were obtained from the NIST Chemistry WebBook (https://webbook.nist.gov/chemistry/) and employed in the calculation of ΔH and ΔS of the reaction. The Gibbs free energy ΔG was calculated from the ΔH and ΔS values with the formula $\Delta G = \Delta H - T\Delta S$ at different temperatures. Afterwards, the reaction equilibrium constant (*K*) was calculated according to following equation:

$$\Delta G = -RT \ln K$$

with the gas constant *R* (8.314 J /mol·K) and the absolute temperature *T* (K).

The equilibrium conversion x can be calculated with a H_2:CO_2 ratio of 3:1 according to:

$$K = \frac{a[\text{CO}] \times b[\text{H}_2\text{O}]}{c[\text{H}_2] \times d[\text{CO}_2]} = \frac{x^2}{(1-x)(3-x)}$$

Here, [CO], [H_2O], [H_2] and [CO_2] represent the concentrations of CO, H_2O, H_2 and CO_2 at this temperature. a, b, c, and d, are stoichiometric coefficients in the chemical equation (all equal to 1 in rWGS reaction). The calculated results match well with the study by Shekari et al.[10] and the result from the Gaseq software (http://www.gaseq.co.uk/).

DFT Calculations (Computational Methods)

First-principle calculations based on Density Functional Theory (DFT), as implemented in the plane-wave VASP code[1 2], were used to optimize the geometry and investigate the electronic properties of the systems. The Projector Augmented Wave (PAW)[3] method was employed to treat the core electrons, and the Perdew-Burke-Ernzerhof (PBE)[4] exchange-correlation functional was used to solve the Kohn-Sham[5] equations. A Γ-centred k-point grid was used for the interface configurations, and the wave functions were expanded using a kinetic energy cut-off of 500eV. Non-spin polarized criterion was used in our calculations. The initial coordinates for CeO_2 were taken from the Materials Project[6] database and relaxed using appropriate k-point sampling. The (111) surface was cleaved from the relaxed bulk structure using the Atomic Simulation Environment (ASE)[7]. To provide an accurate description of the CeO_2 bandgap, DFT+U method with U=5eV[8] was employed. To create the (111) surface of CeO_2, we first relaxed a CeO_2 unit cell. Coordinates of the unit cell were taken from Materials Project (structure id mp-20194, phase: cubic, space group Fm$\bar{3}$m), containing 4 Ce atoms and 8 oxygen atoms. The unit cell was relaxed using a Γ-centered 4x4x4 k-point grid, while a threshold of 0.03eV/Å for atomic residual forces was used. To model both the surface and bulk regions, atoms in the middle layer (referred to as "Bulk" in **Figure S29a**) were fixed, while the top and bottom layers (referred to as "Surface" in **Figure S29a**) were allowed to relax. In the pristine CeO_2 structure, two oxygen vacancies on the top as well as bottom surfaces, creating a structure with symmetric oxygen vacancies (see Figure **S29b**). After relaxation, the lattice parameters of the unit cell are as follows: a = b = c = 5.496 Å. (**Figure S30**)

For the copper-substituted structures (**Figure S31**), one of the Ce atoms adjacent to the oxygen vacancies was replaced with a Cu atom to create Cu_1-substituted CeO_2. Similarly, one of the Ce atoms adjacent to the oxygen vacancies was replaced with two Cu atom to create Cu_2-substituted CeO_2. As shown in **Figure S33-34**, the averaged copper oxidation state for copper lay between +1 to +2 for Cu_1-substituted CeO_2 and lay between 0 to +1 for Cu_2-substituted CeO_2. The surface is maintained charge neutral for these two structures. In addition, a hybrid Cu_3-substituted CeO_2 structure was constructed by introducing one and two Cu atoms into two separate Ce vacancies, respectively. For CeO_2-supported Cu structure, a Cu_5[9] cluster was geometrically optimized and then placed on the surface. For the (111) slab of CeO_2 and the subsequently modified systems of Cu-doped and Cu-cluster, we have relaxed the supercell using a Γ-centered 1x1x1 k-point grid, with a convergence criterion of 10^{-3} eV energy

difference between two subsequent ionic steps. The vacuum applied is a total of 20 Å along the z-axis. The pristine (111) slab model of CeO_2 contains 48 Ce atoms and 96 O atoms. 1 Ce and 2 O atoms were removed from top as well as bottom surfaces. Additionally, for the single Cu atom doped, two Cu atom doped and the Cu_5 cluster modified CeO_2 (111) surface contains 1, 2, and 5 Cu atoms respectively.

For the unit cell of Cu_2O and CuO (**Figure S30**), the initial coordinates were taken from the Materials Project database (id: mp-361, mp-704645, respectively). Cu_2O structure contains 4 Copper and 2 oxygen atoms, having cubic phase with space group of $Pn\bar{3}m$. CuO, on the other hand, contains 4 Copper atoms and 4 Oxygen atoms, having a monoclinic phase with space group of C2/c space group. For the convergence of the unit cells of these structures, the convergence criterion was the same as that employed for the CeO_2 unit cell.

For oxidation state calculation, Bader Analysis[11] was used. Charge density mapping was visualized using the XCrySDen software while the all the structures were visualized using VESTA. Further discussion on oxidation state of copper is included in the **Supplementary discussion S1.**

The adsorption energy of the clusters on the substrate was calculated using the below-mentioned equation

$$E_{ads}^{Molecule+Surface} = E_{Total}^{Molecule+Surface} - (E_{Total}^{Molecule} + E_{Total}^{Surface}) \quad (1)$$

Where $E_{ads}^{Molecule+Surface}$ is the adsorption energy of the cluster on the surface, $E_{Total}^{Molecule+Surface}$ is the total energy of the combined cluster/surface system, $E_{Total}^{Molecule}$ and $E_{Total}^{Surface}$ are the total energy of the individual molecules and surface slab relaxed to their optimized geometries, respectively.

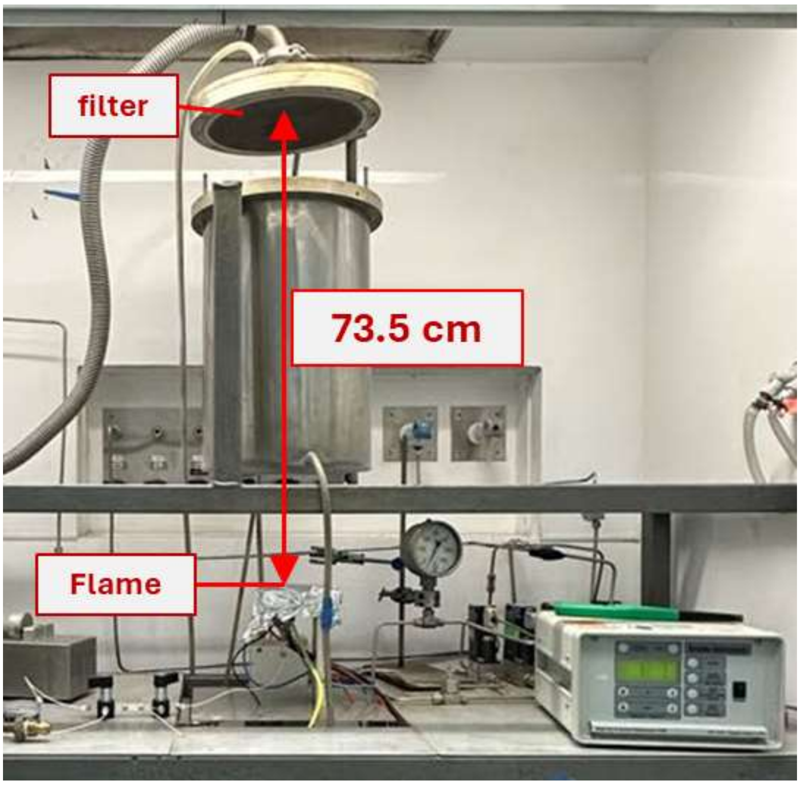

Figure S1. A photograph of the FSP experimental setup.

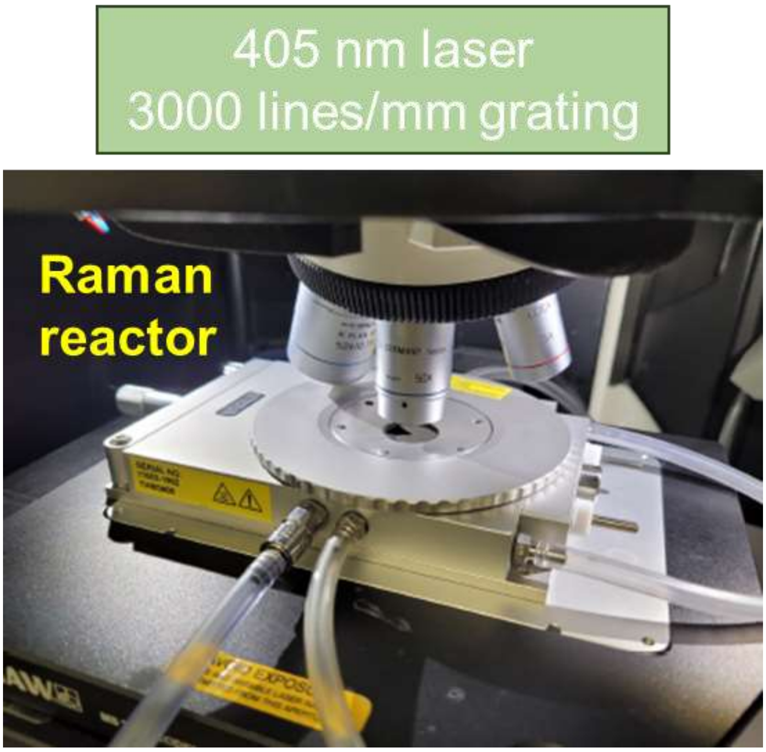

Figure S2. Photo of the in-situ Raman set-up.

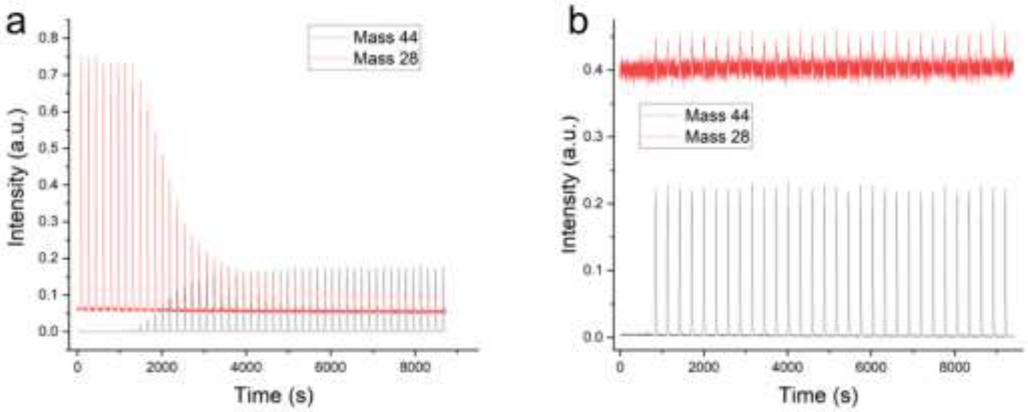

Figure S3. Evolution of N_2O (MS = 44) and N_2 (MS = 28) signals during N_2O titration experiments for reduced (**a**) 5CuCe-FSP and (**b**) ceria support. In (b), only a weak MS = 44 signal corresponding to unreacted N_2O is observed, and no significant N_2 formation is detected, indicating negligible N_2O decomposition on bare ceria. This confirms that under the applied conditions, the titration selectively probes metallic Cu^0 sites.

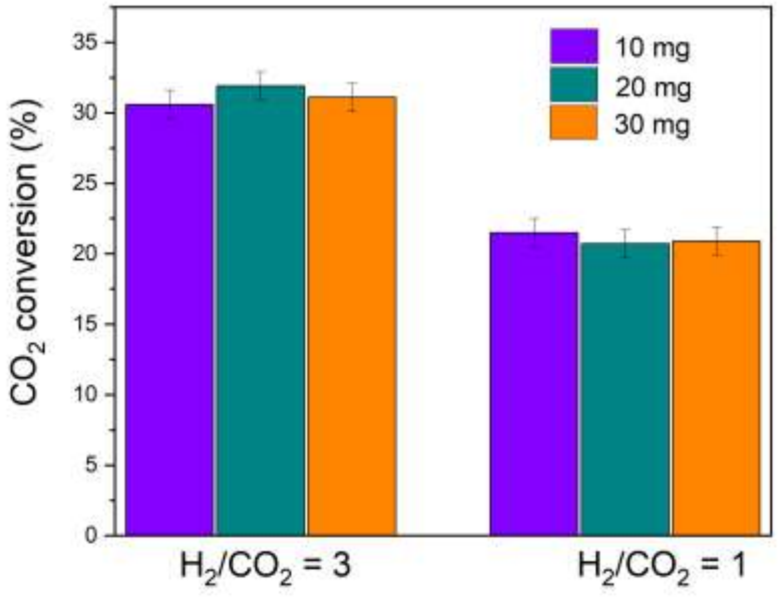

Figure S4. Evaluation of external mass transfer limitations by varying catalyst loading (10–30 mg) while maintaining a constant GHSV (i.e., contact time). Catalysts were diluted with silica at a 10:1 diluent-to-catalyst ratio. Two H_2/CO_2 ratios (3 and 1) were tested for each loading. Experimental conditions: 500 °C; GHSV = 272,400 mL/$g_{cat.}$/h (H_2/CO_2 = 3) and 201,600 mL/$g_{cat.}$/h (H_2/CO_2 = 1).

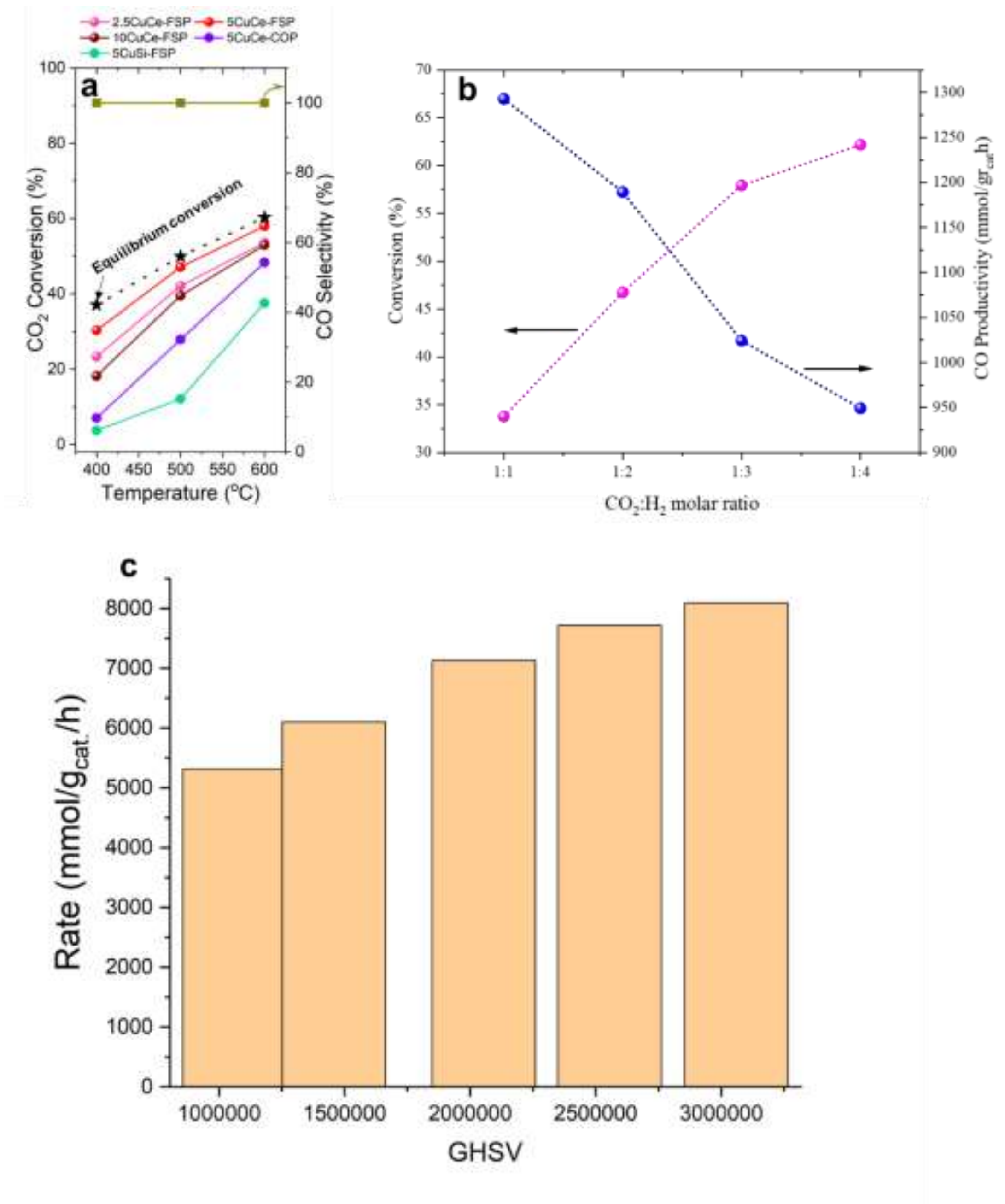

Figure S5. (**a**) Catalyst performance (CO_2 conversion and CO selectivity) of the catalysts prepared for this study. 10 mg catalyst diluted with silica were tested at GHSV = 218,400 mL/$g_{cat.}$/h. (**b**) The effect of H_2/CO_2 ratio on CO_2 conversion and CO productivity at fixed GHSV = 381,800 mL/g_{cat}/h and 600 °C. (**c**)The effect of GHSVs on the CO production rate for 5CuCe-FSP. Reaction condition: H_2/CO_2 = 3, N_2 = 4 mL/min, T = 600°C. 4 mg catalyst was diluted with 200 mg silica. Increasing the H_2/CO_2 ratio enhances the surface coverage of reactive hydrogen, thereby promoting the rate-determining hydrogenation steps and improving overall CO_2 conversion. However, CO productivity depends not only on conversion but also on the total amount of CO_2 fed. At higher H_2/CO_2 ratios and fixed GHSV, the CO_2 feed rate becomes lower, which reduces the absolute amount of CO formed, even if the conversion increases—leading to a decrease in apparent productivity.

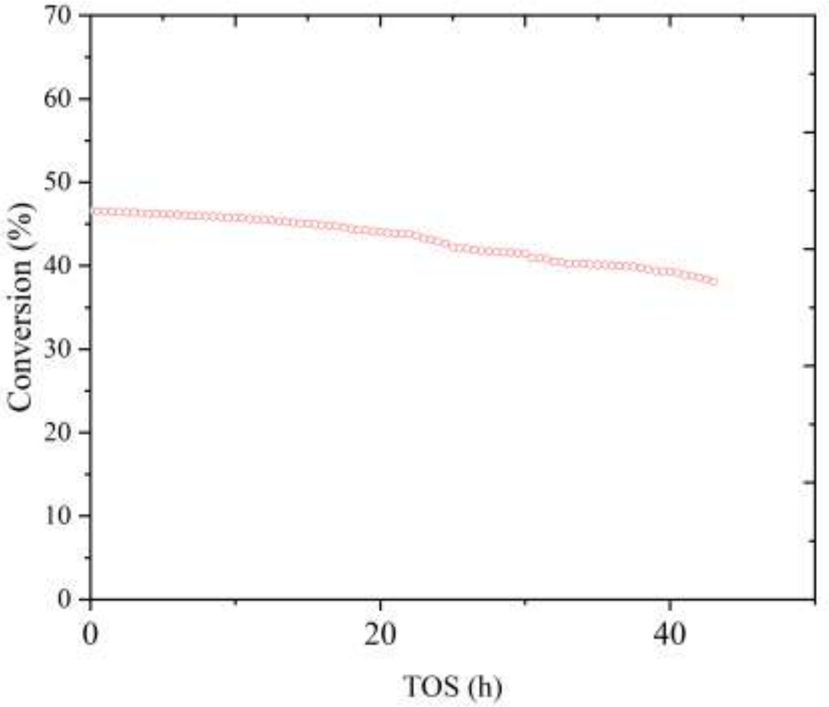

Figure S6. Performance stability of 5CuCe-COP at 600°C at GHSV = 376,800 mL/g/h.

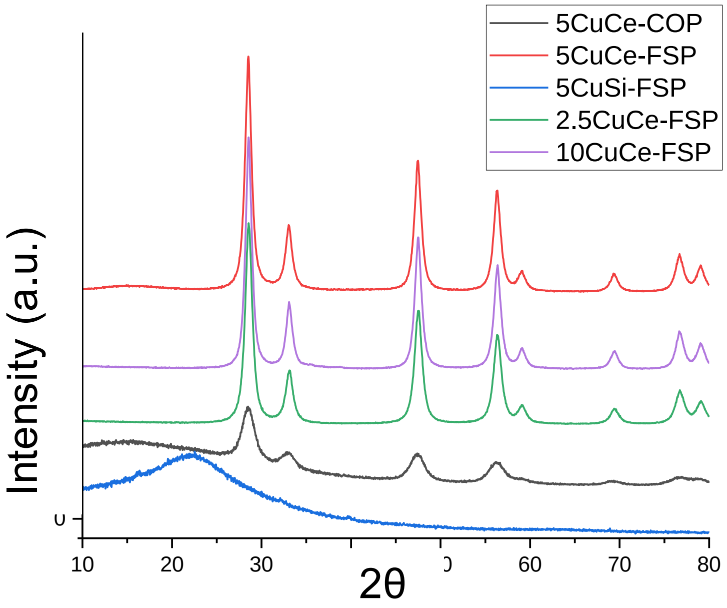

Figure S7. XRD patterns of the as-prepared copper-ceria samples. The estimated CeO_2 crystal size is 9.5 ± 1 nm, no CuO peaks observed, and no shift in the CeO_2 peak position.

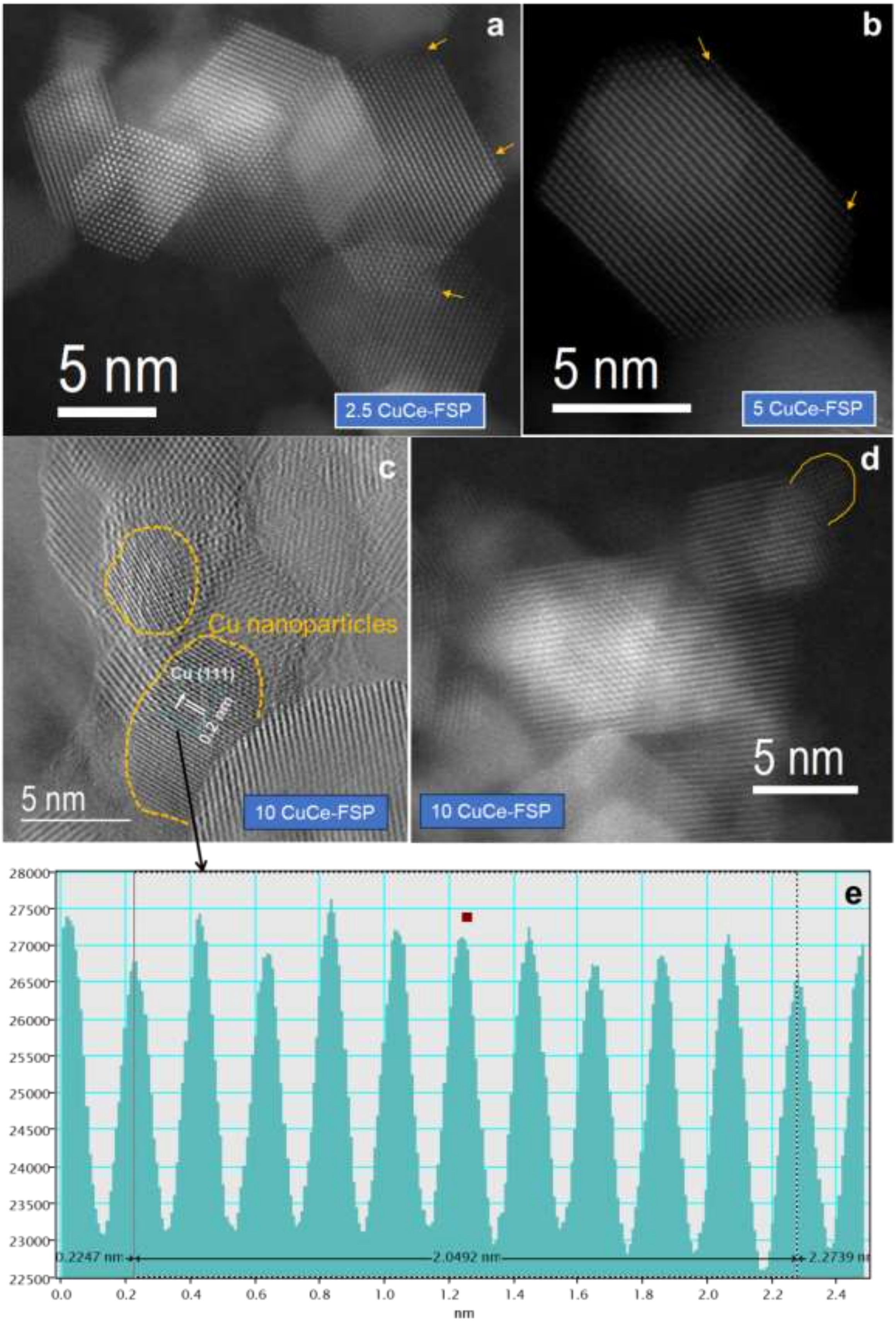

Figure S8. STEM images of (**a**) 2.5CuCe-FSP, (**b**)5CuCe-FSP, and (**c-d**)10CuCe-FSP. (**e**) Corresponding height profile of the Cu region marked in (c). The copper particles are marked by circles and the copper clusters, marked by arrows, are likely anchored at the surface/edge of the ceria particles.

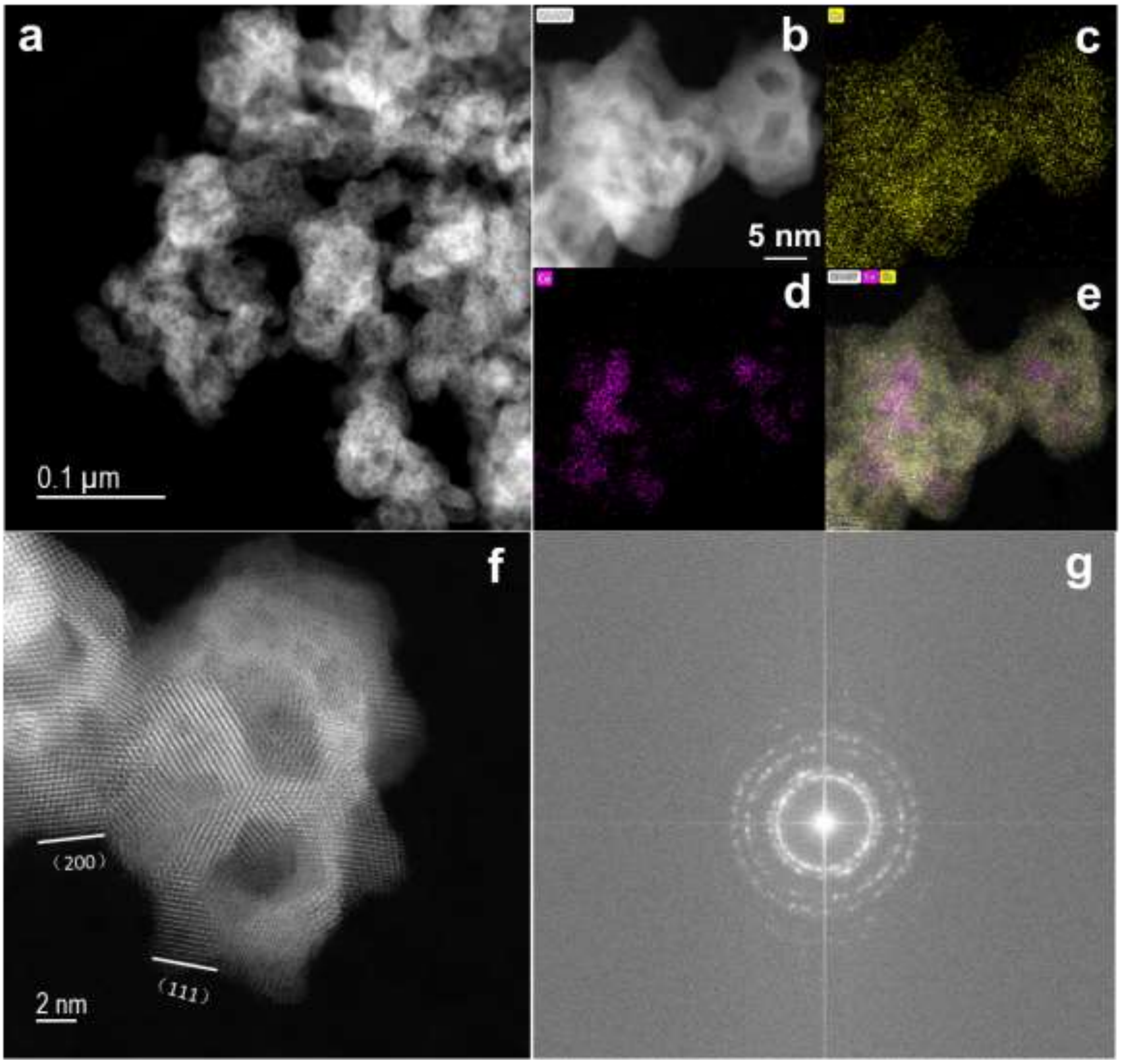

Figure S9. (**a, b, f**) STEM images of 5CuCe-COP with (**c-d**) corresponding EDX mapping. (**g**) Selected area diffraction (SAD) pattern of the region shown in (f). The probability is high for an encapsulation of the copper species by CeO_2 as shown in EDX maps where copper and ceria overlap. Different to FSP-made samples where the CeO_2 (111) lattice plane dominates, multiple crystal lattice planes of CeO_2 were observed (d_{111}(3.13 Å); d_{200}(2.71 Å); d_{220}(1.91 Å) for 5CuCe-COP. The presence of irregular and mismatched fringes also indicates the presence of ceria aggregates consisting of epitaxially interfaced nanocrystallites.

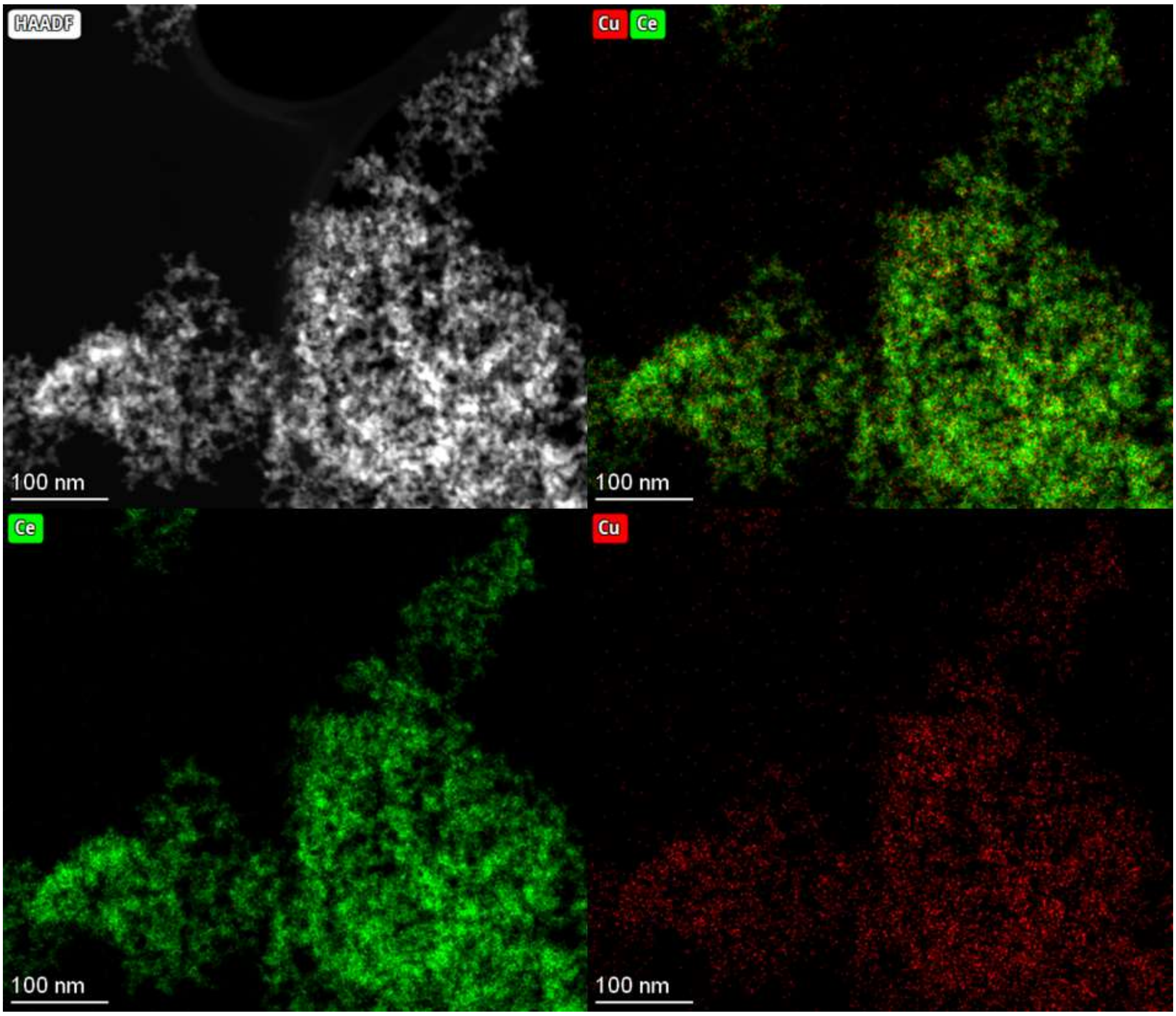

Figure S10. Supplementary STEM and EDX images of 2.5CuCe-FSP.

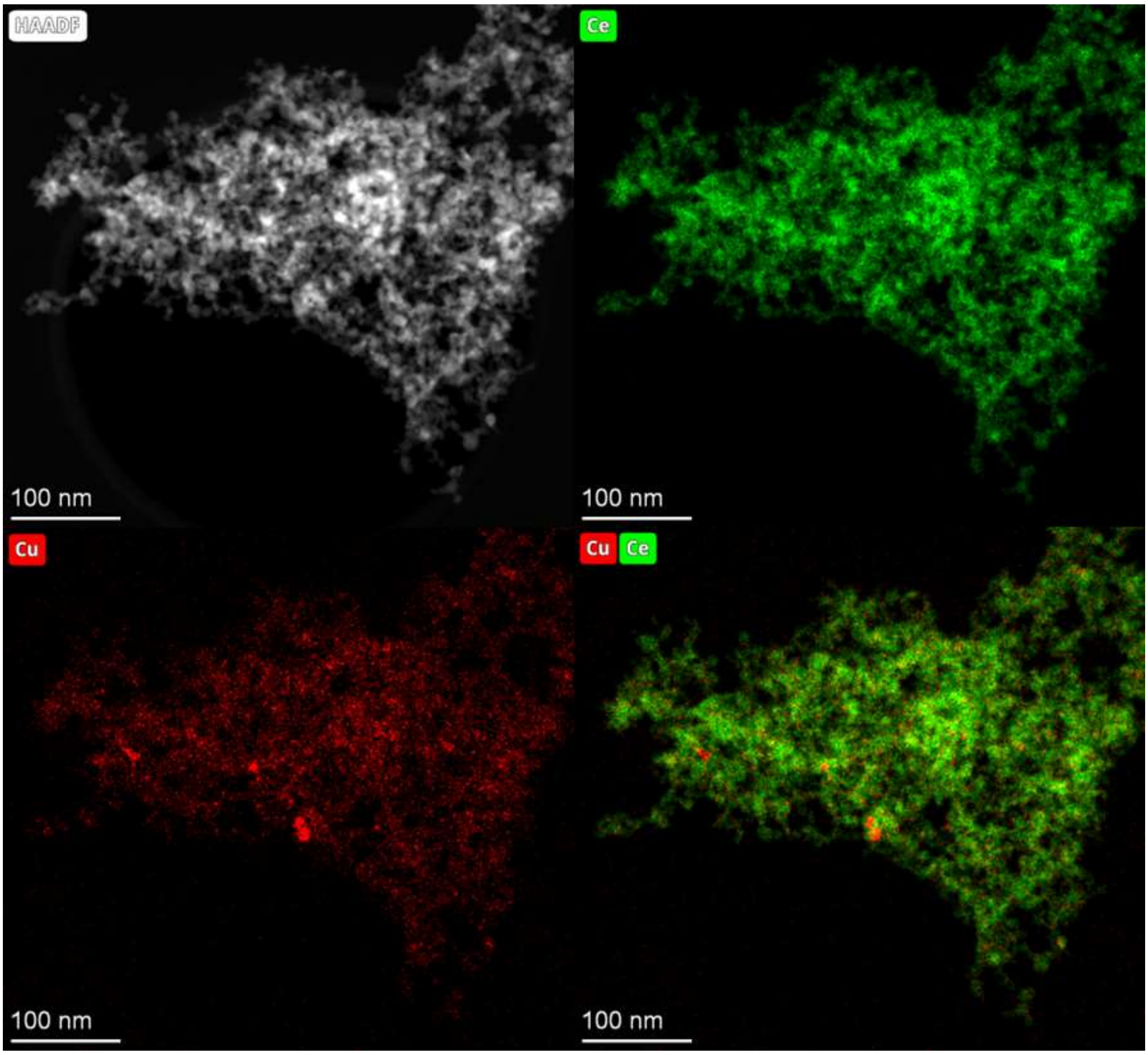

Figure S11. Supplementary STEM and EDX images of 5CuCe-FSP.

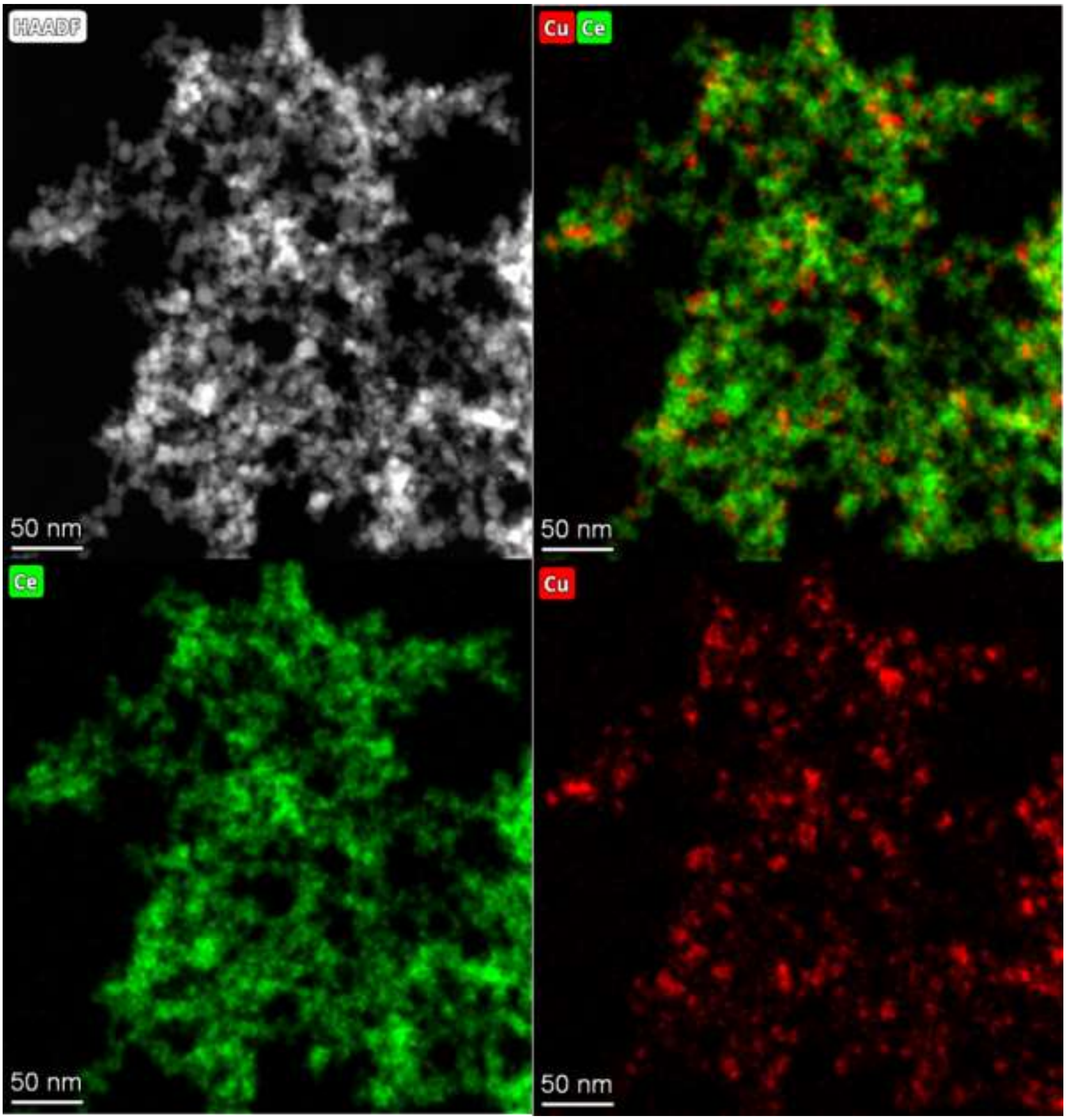

Figure S12. Supplementary STEM and EDX images of 10CuCe-FSP.

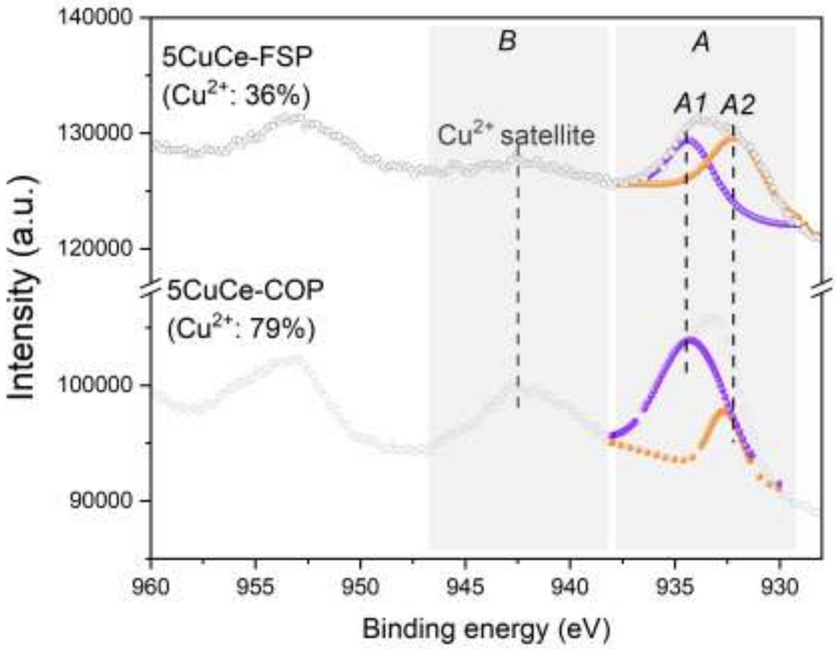

Figure S13. Cu 2p spectra of 5CuCe-FSP and 5CuCe-COP. The main Cu $2p_{3/2}$ peak (*A*) is deconvoluted into Cu^{2+} (*A*1) and Cu^0/Cu^+ (*A*2). The satellite feature (*B*) is entirely from Cu^{2+}. The deconvolution is mainly a visual guide to support qualitative assessment. As reported in the study of Biesinger et al.[12], the relative concentration of Cu^{2+} was derived from the Cu $2p_{3/2}$ spectra by the equation: $\%Cu^{2+} = \frac{B+A1}{A+B} \times 100 = \frac{B(1+(\frac{A1s}{Bs}))}{A+B} \times 100$, where $\frac{A1s}{Bs}$ is taken from the reported CuO standards[12] (1.89).

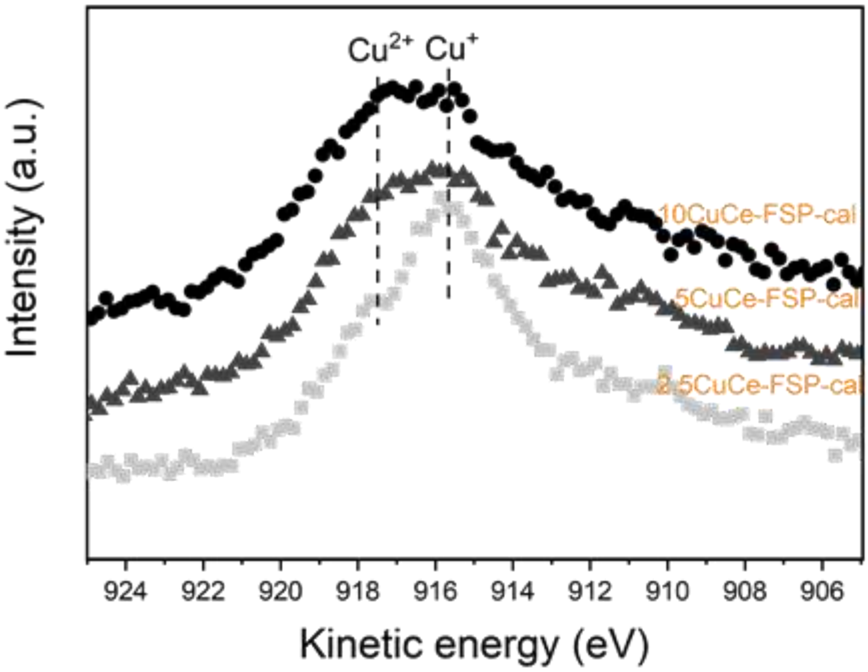

Figure S14. XPS Cu LMM Auger spectra of the calcined (unreduced) CuCe-FSP samples.

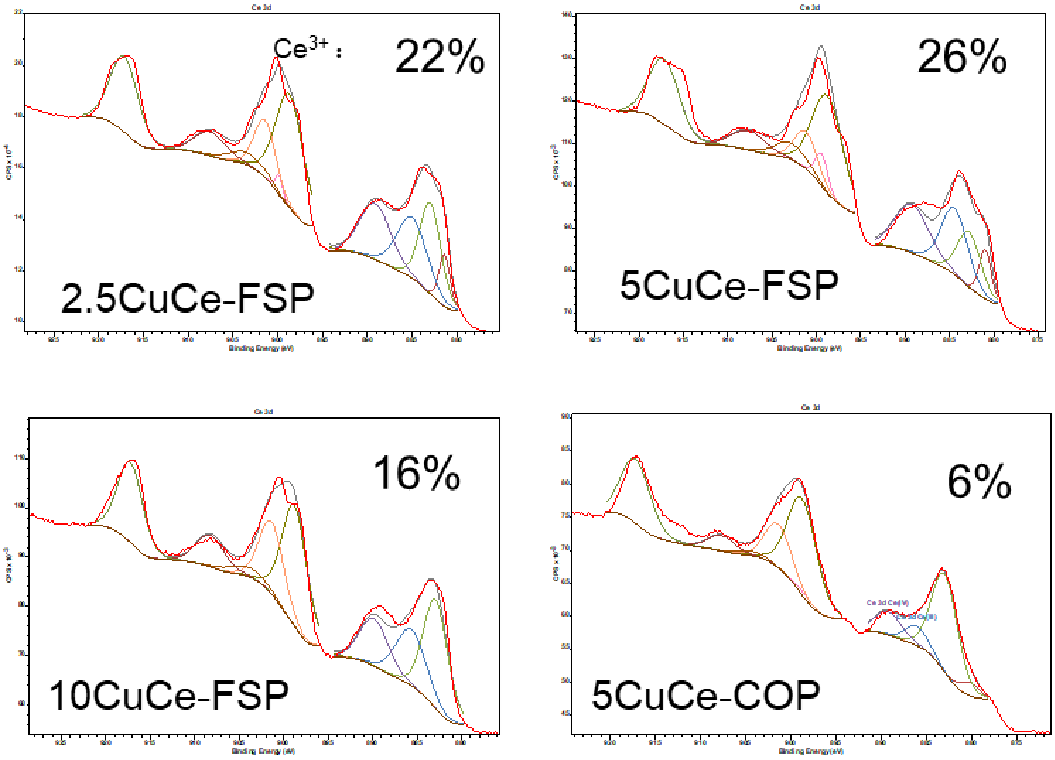

Figure S15. XPS Ce 3d spectra of different calcined (unreduced)samples ($Ce^{3+}/(Ce^{3+}+Ce^{4+})$ fraction included in the figure).

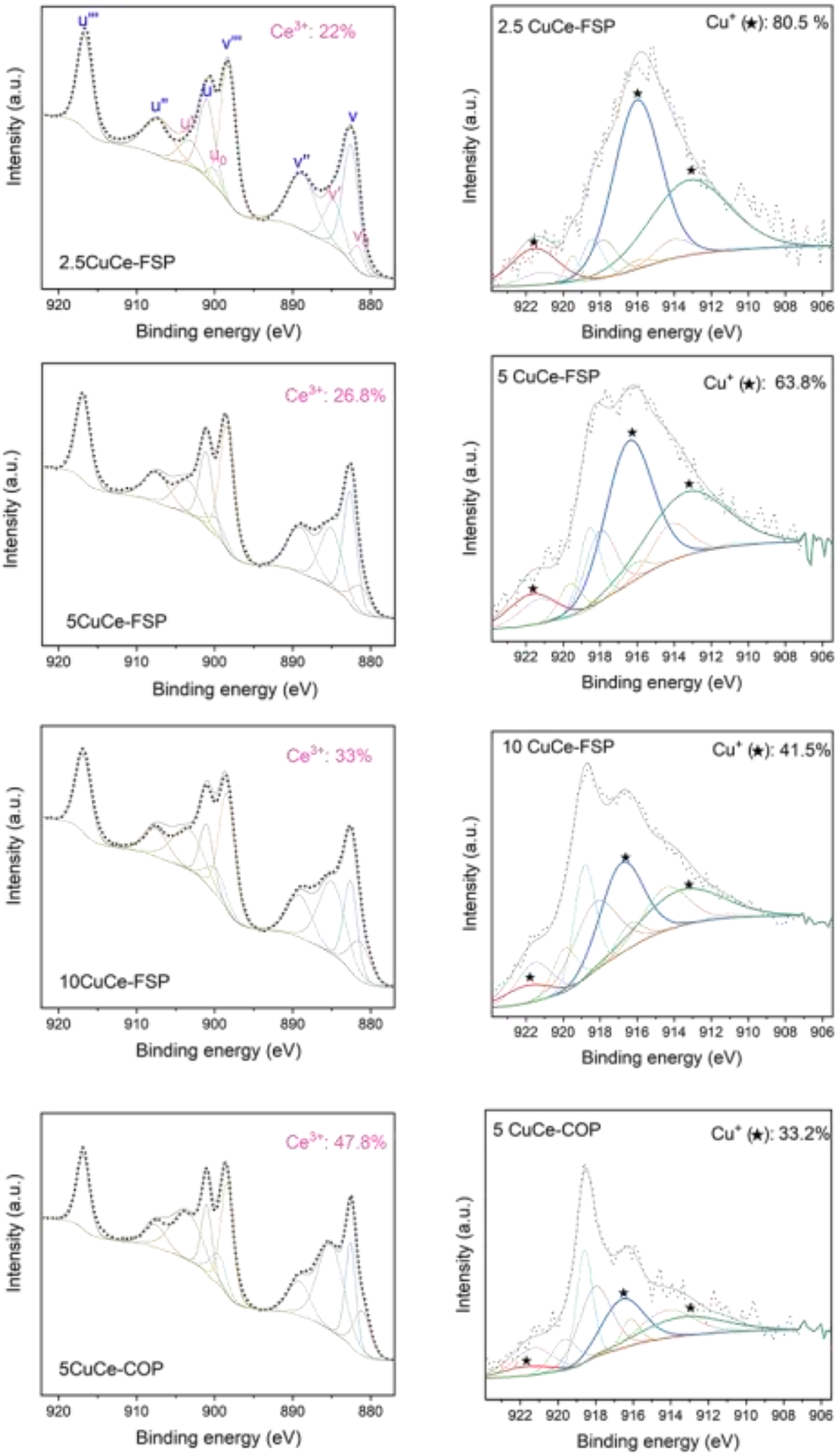

Figure S16. Quantitative analysis of the XPS spectra to determine the (left) Ce^{3+} and (right) Cu^{+} concentrations in the reduced samples. Deconvolution of the Cu LMM spectra was achieved using a linear fit combination based on the reference Auger spectra of Cu, Cu_2O, and CuO. Detailed method can refer to our previous study.[5]

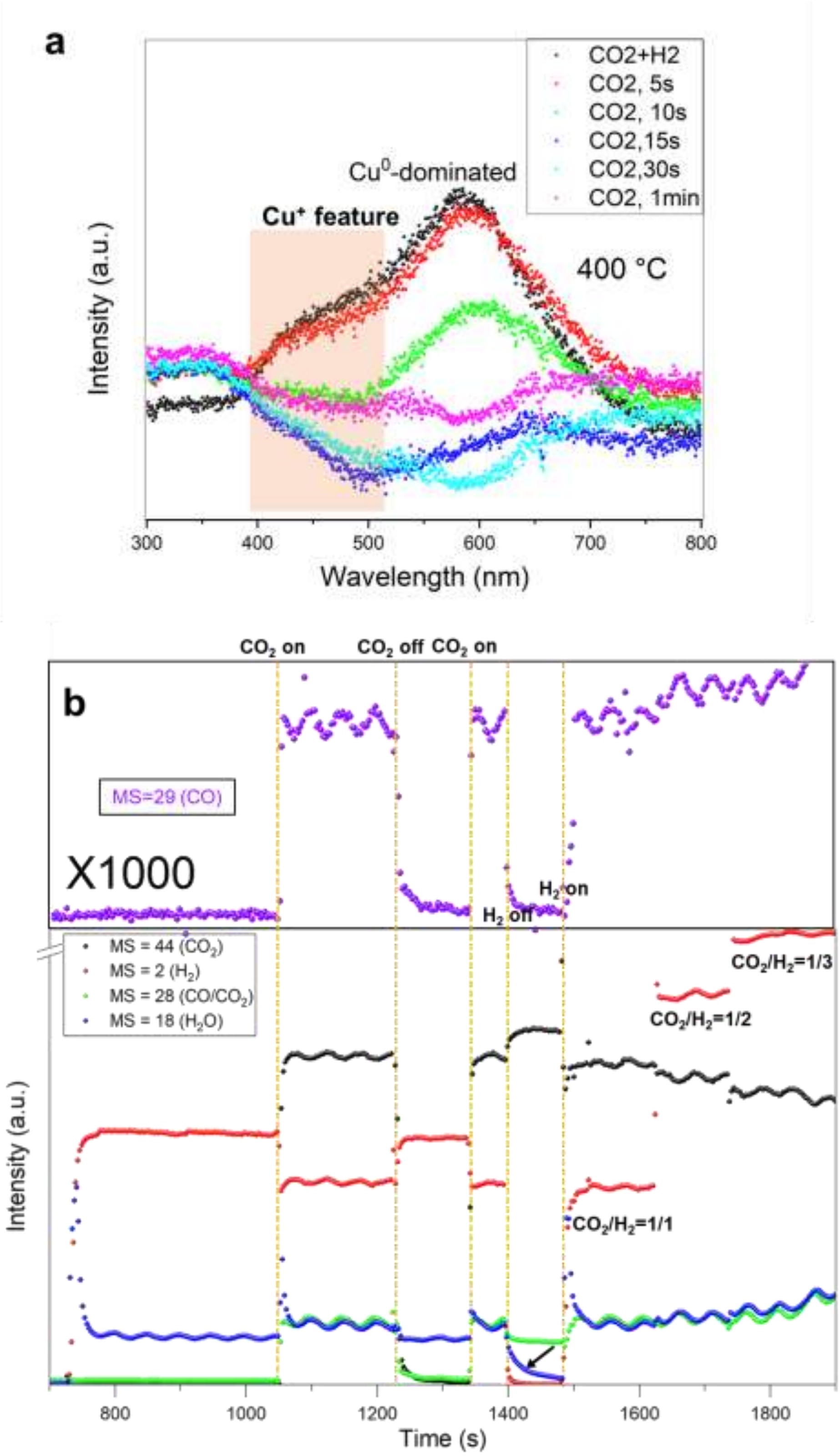

Figure S17. (**a**) The in-situ UV-vis spectra revealed a dynamic transition in the Cu oxidation state upon switching the reaction feed to CO_2 only. (**b**) Gas species during in-situ UV-Vis experiments monitored by on-line MS spectrometry.

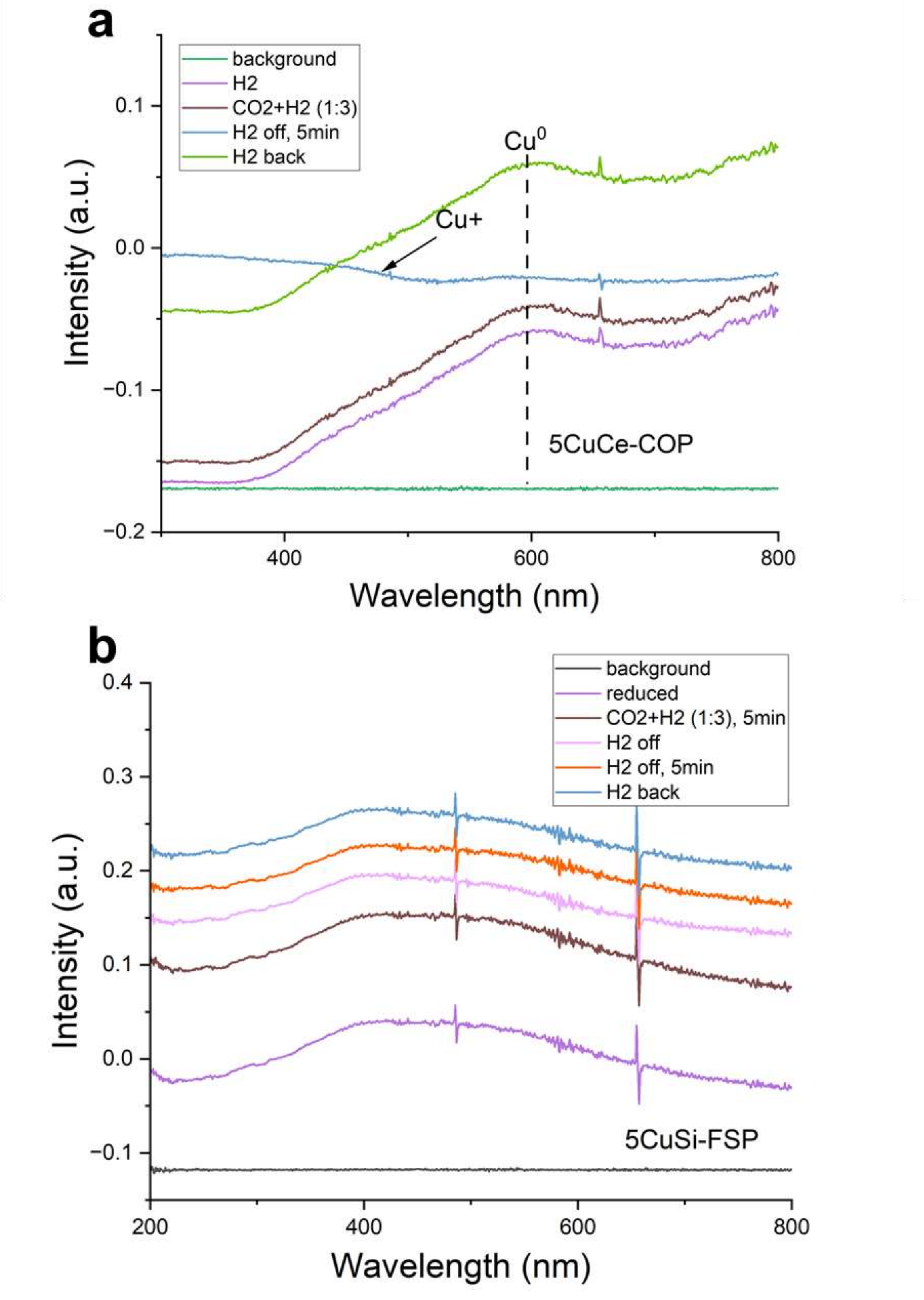

Figure S18. In-situ UV-Vis spectra for (**a**) 5CuCe-COP and (**b**) 5CuSi-FSP at different reaction conditions. Unlike CuCe samples, there is no clear change in Cu state for 5CuSi-FSP when switching from CO_2+H_2 to CO_2-only condition.

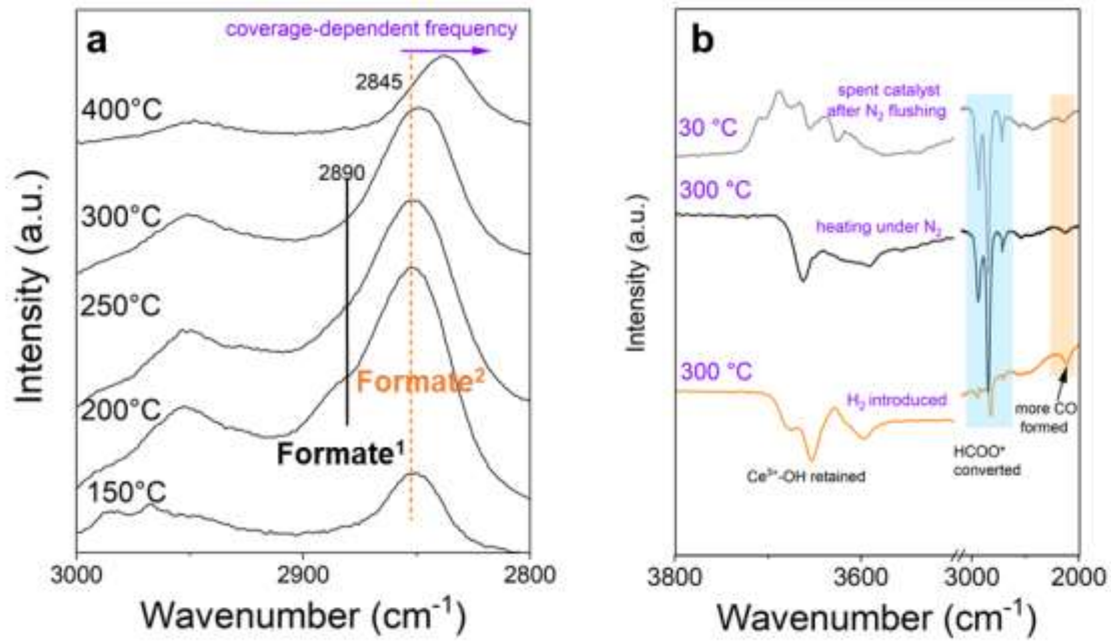

Figure S19. (**a**) Temperature-dependent DRIFTS spectra in the C–H vibrational region during the rWGS reaction. The C–H stretching mode of formate at ~2845 cm^{-1} exhibits a red-shift as its surface coverage decreases with increasing temperature. Spectra were acquired under a reduced total flow rate of 12 mL/min (N_2:CO_2:H_2 = 2:1:3) compared to standard conditions, in order to retain more surface formate species. Additionally, a second C–H band at ~2890 cm^{-1}, tentatively assigned to formate species located near copper sites, was also observed. (**b**) DRIFTS spectra collected at three stages: after the reaction and cooling to 30 °C under N_2, during heating to 300 °C under N_2, and after H_2 reintroduction at 300 °C.

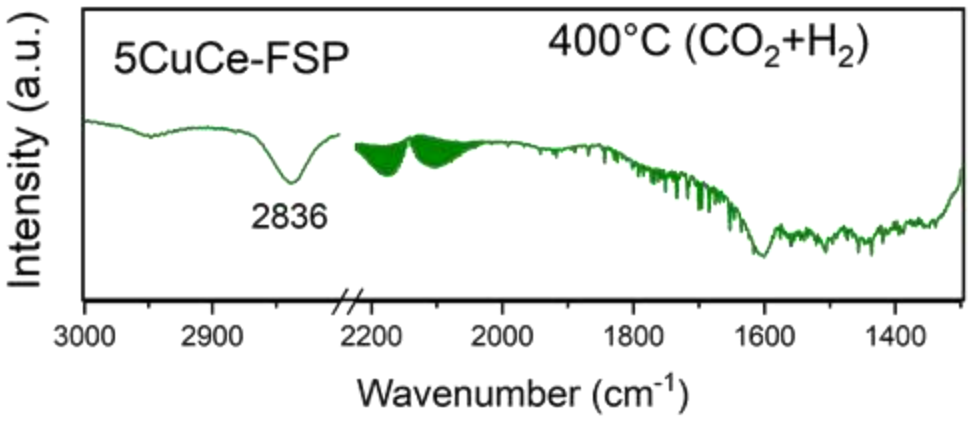

Figure S20. In-situ DRIFTS spectra of 5CuCe-FSP at 400 °C. Strong signal of gaseous CO and water appeared at 2000-2200 cm^{-1} and 1400-1900 cm^{-1}, respectively.

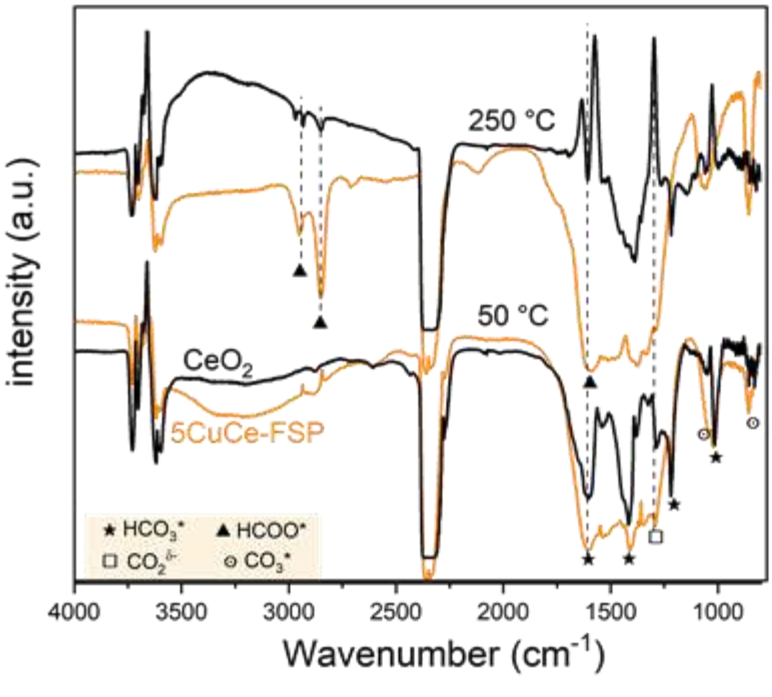

Figure S21. Comparison of DRIFT spectra of neat CeO_2 and 5CuCe-FSP during reaction. Reaction condition: N_2:CO_2:H_2 ratio = 2:1:3, total flowrate = 30 mL/min.

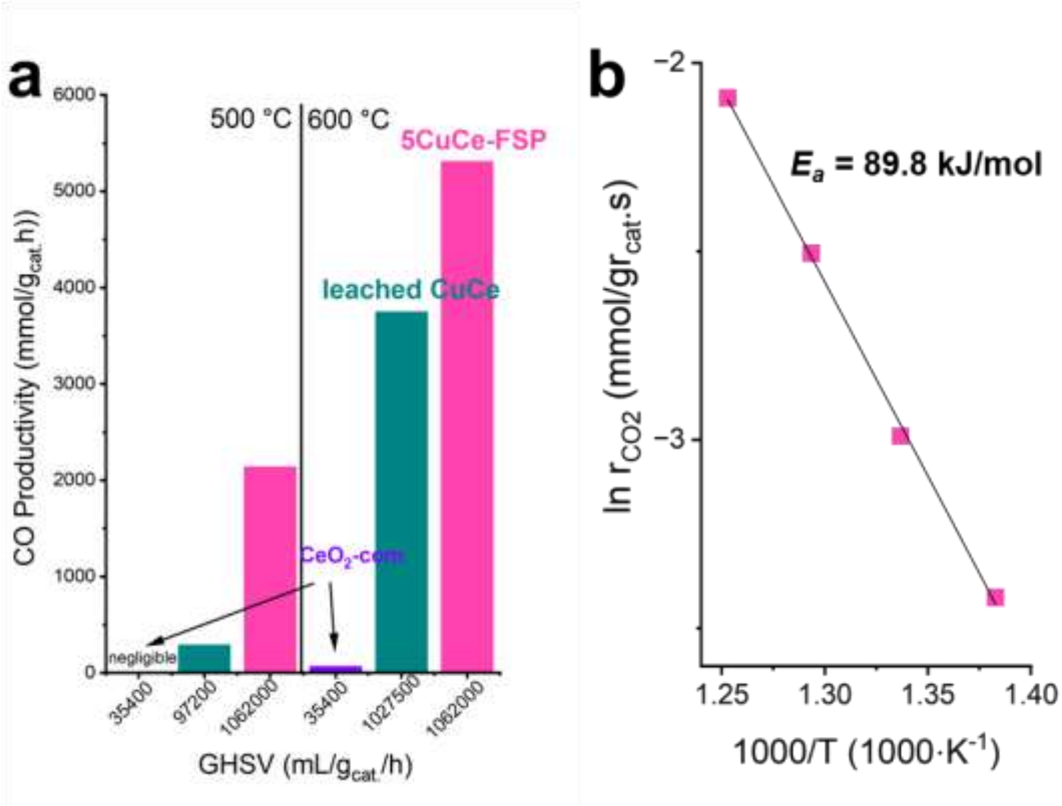

Figure S22. (**a**) Catalytic activity and (**b**) Arrhenius plot of leached 5CuCe-FSP. The commercial ceria (CeO_2-com) was also tested for comparison.

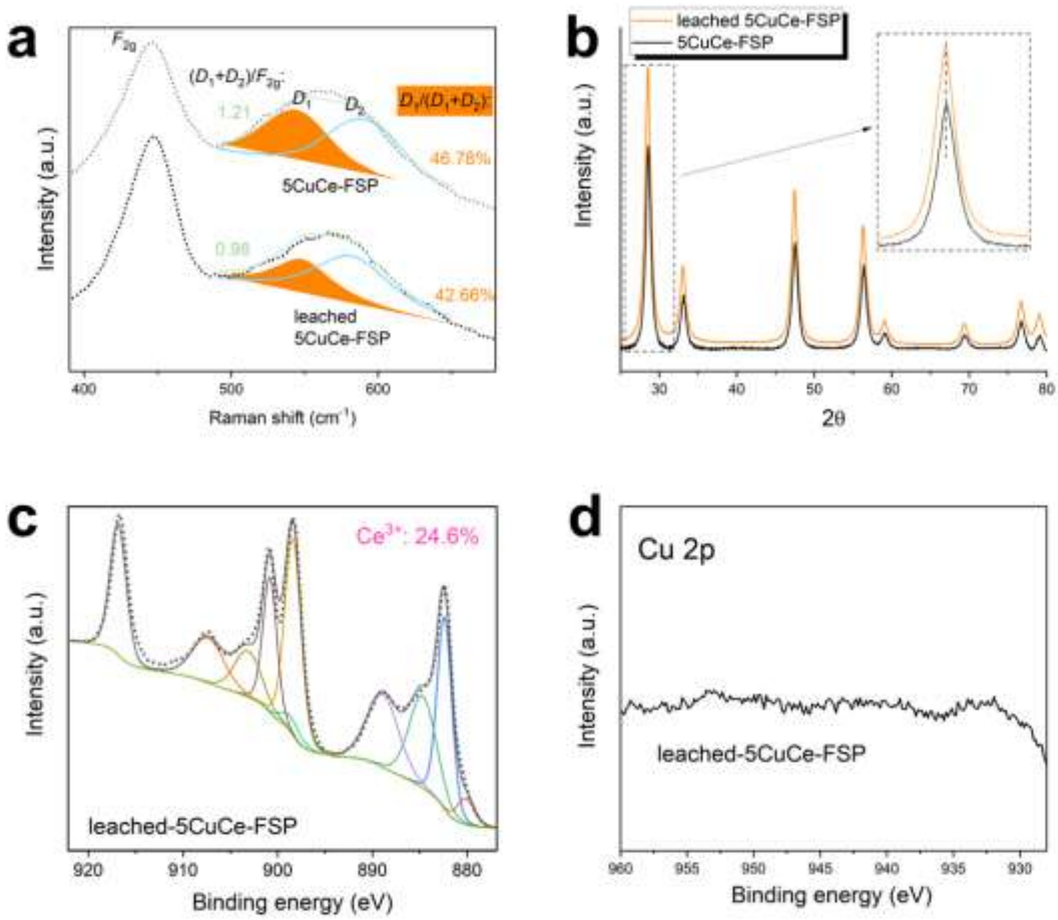

Figure S23. Characterization of 5CuCe-FSP before and after nitric acid leaching: (**a**) Raman spectra for ceria defect structure, (**b**) XRD patterns for crystallinity, (**c**) XPS Ce 3d, and (**d**) XPS Cu 2p of the leached sample. Prior to Raman measurement, the sample was reduced at 400 °C under 50 mL/min of 20% H_2.

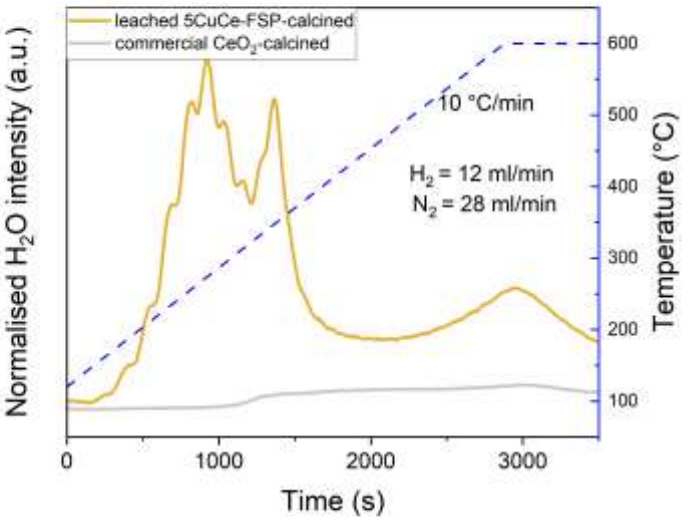

Figure S24. Temperature-programmed reduction of leached 5CuCe-FSP and commercial CeO_2 in reactor system.

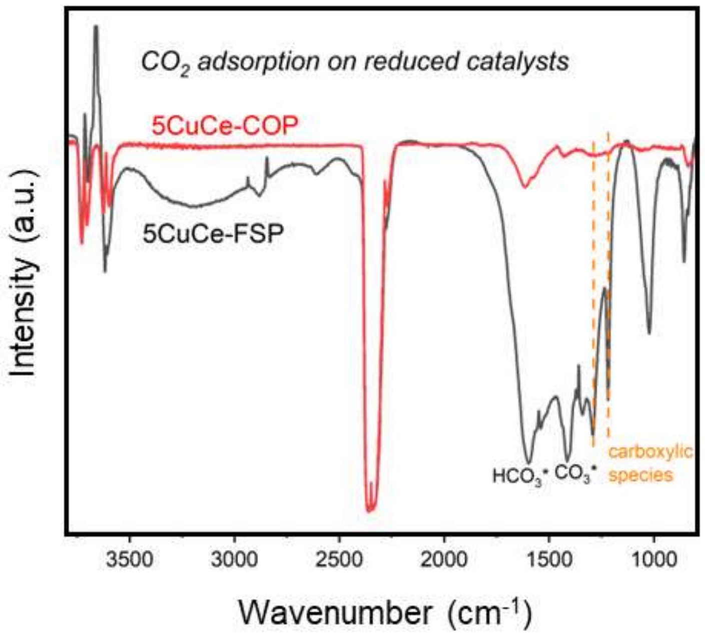

Figure S25. DRIFTS spectra of 5CuCe-COP and 5CuCe-FSP. It shows that more intermediates were formed on 5CuCe-FSP compared to 5CuCe-COP when the reduced catalysts were exposed to CO_2. Reaction condition: N_2:CO_2:H_2 ratio = 2:1:3, total flowrate = 30 mL/min, T = 50 °C.

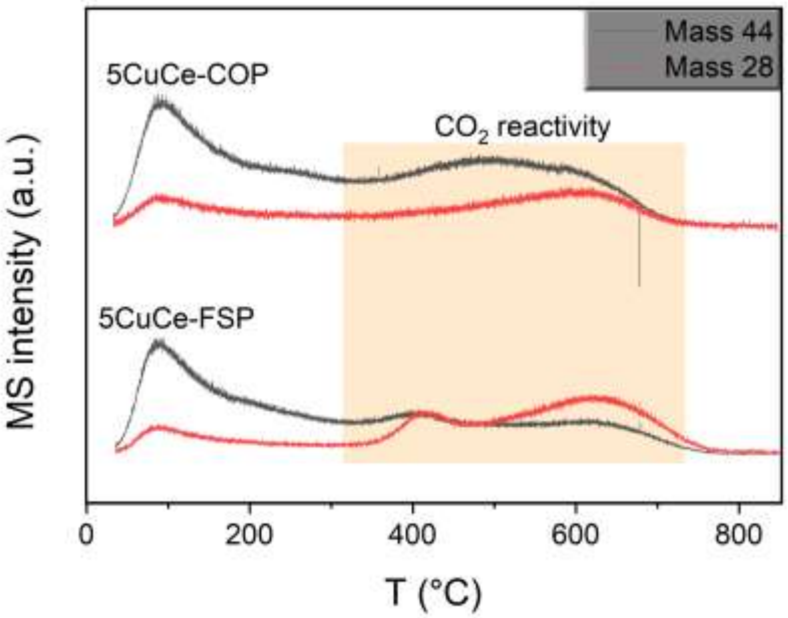

Figure S26. Temperature programmed CO_2 desorption (CO_2-TPD) for 5CuCe-COP and 5CuCe-FSP. Following the CO_2 pulse chemisorption to determine the quantity of ceria defective sites (see experimental method for details), the system was flushed with Ar until no CO_2 signal was detected. The system was then heated to 850 °C under Ar with the CO (mass=28) and CO_2 (mass=44) signals being recorded by MS spectrometry.

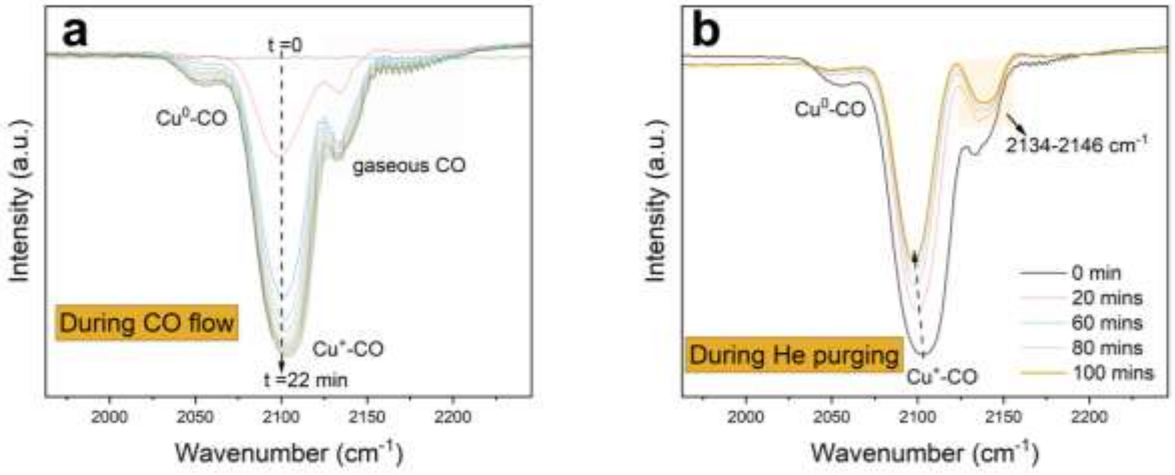

Figure S27. CO-FTIR spectra of the 5CuCe-FSP catalyst at 300 K. (a) Spectra recorded during the first 22 minutes following CO introduction on a pre-reduced sample, showing the evolution of CO adsorption features. (b) Subsequent spectra during He purging, illustrating the gradual desorption of gaseous and weakly bound CO species, while strongly adsorbed Cu^{+}–CO species remain.

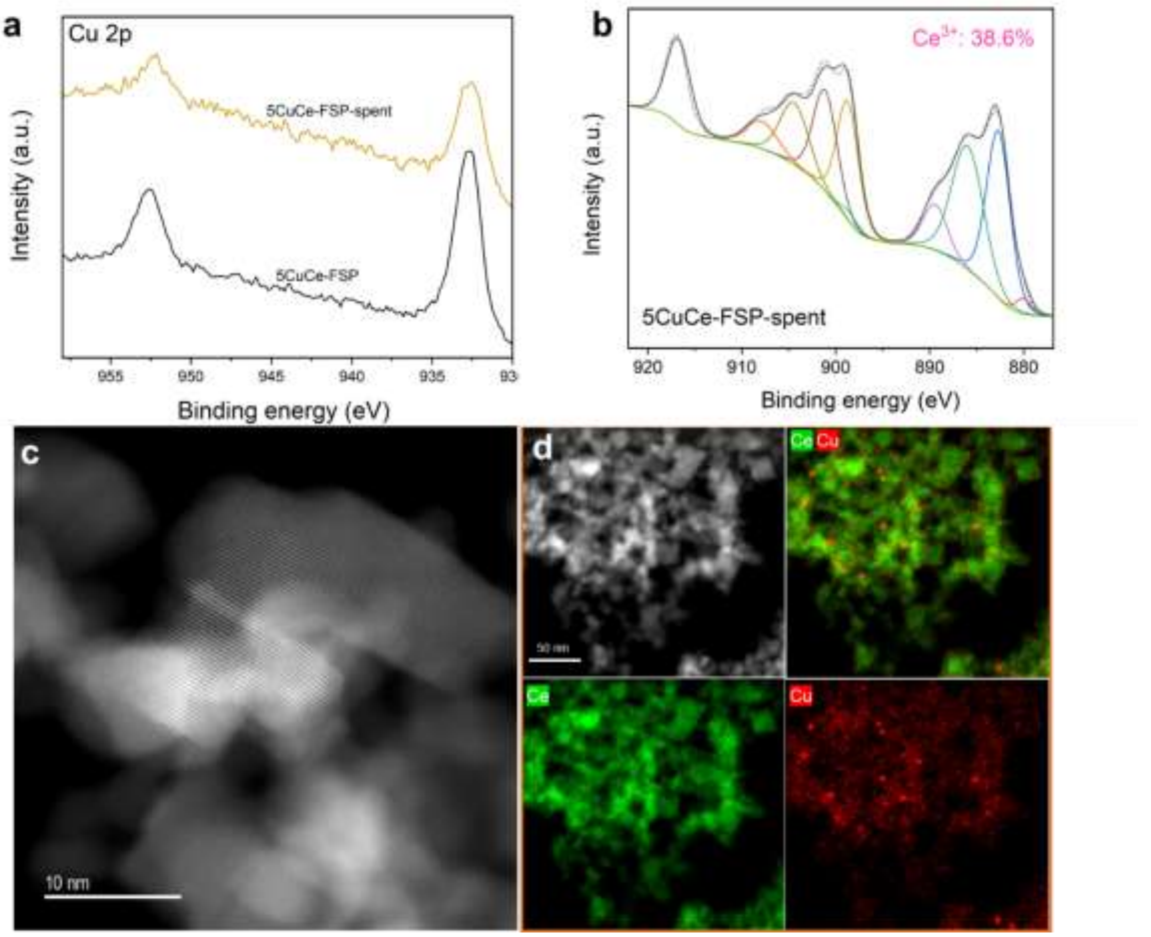

Figure S28. Post-reaction characterization of 5CuCe-FSP after 40 h at 600 °C. (a) Cu 2p XPS spectra; (b) Ce 3d XPS spectra; (c, d) STEM images and corresponding elemental mapping. Results show stable Cu dispersion and partial Ce^{3+} reoxidation, consistent with operando data.

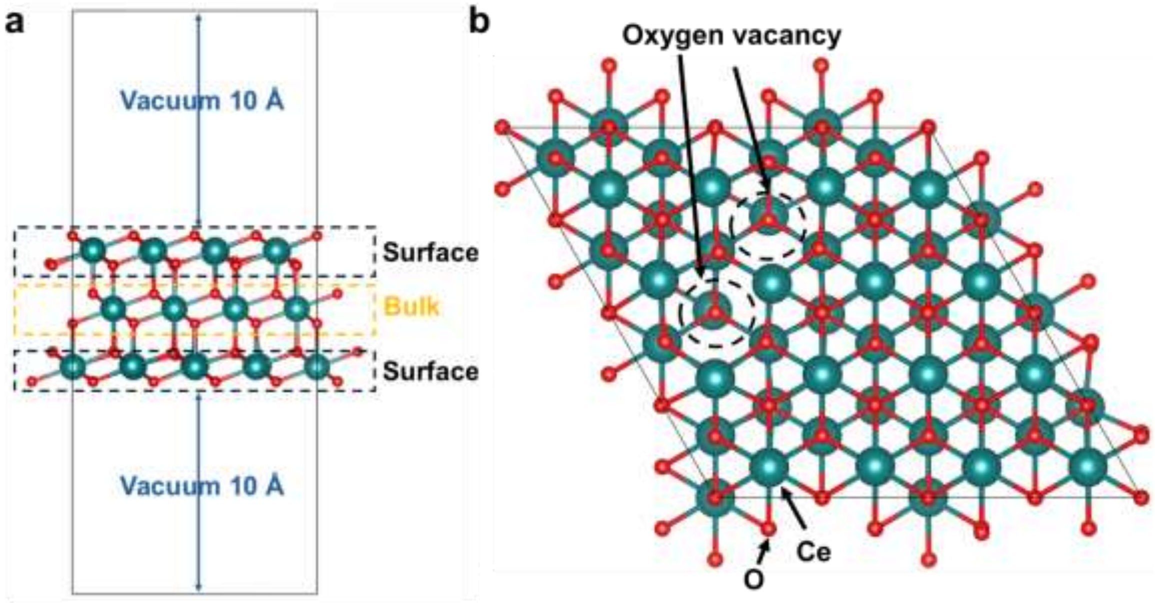

Figure S29. Model structure used in calculations for (**a**) pristine cerium oxide, (**b**) cerium oxide with oxygen vacancies.

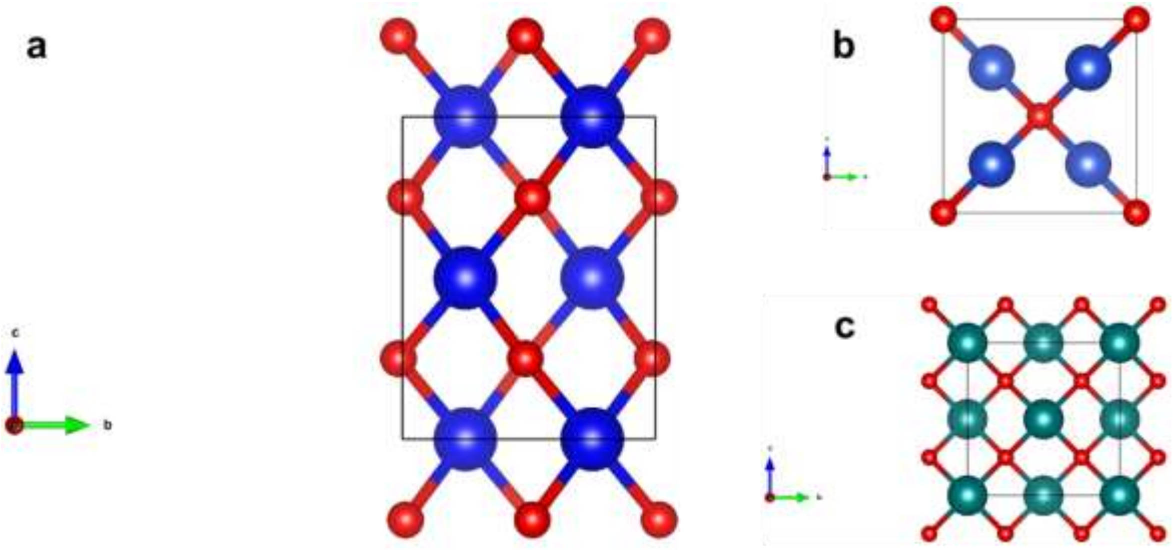

Figure S30. Relaxed unit cell of (**a**) CuO, (**b**) Cu_2O, and (**c**) CeO_2.

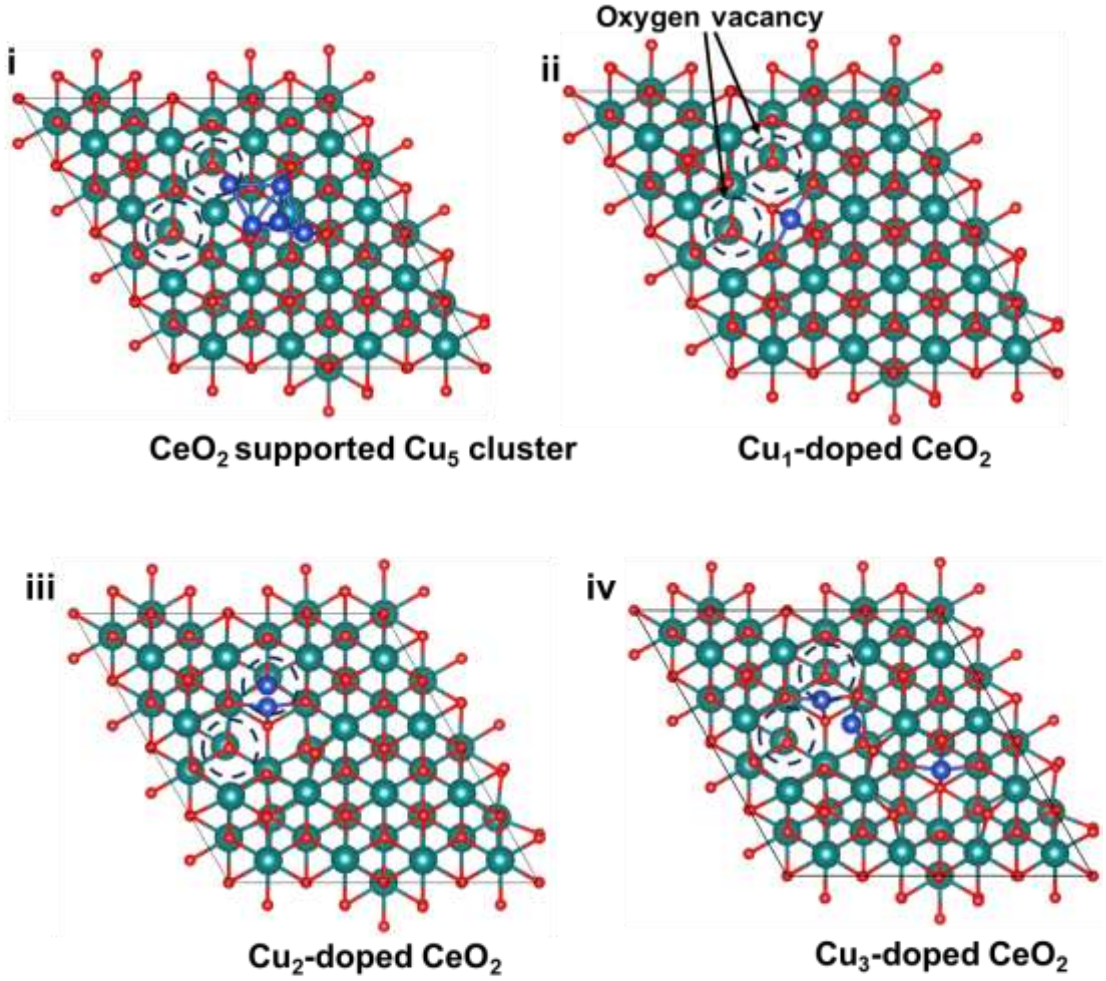

Figure S31. Model structures used for Cu-substituted ceria structure. Blue (Copper), red (Oxygen), cyan (Cerium).

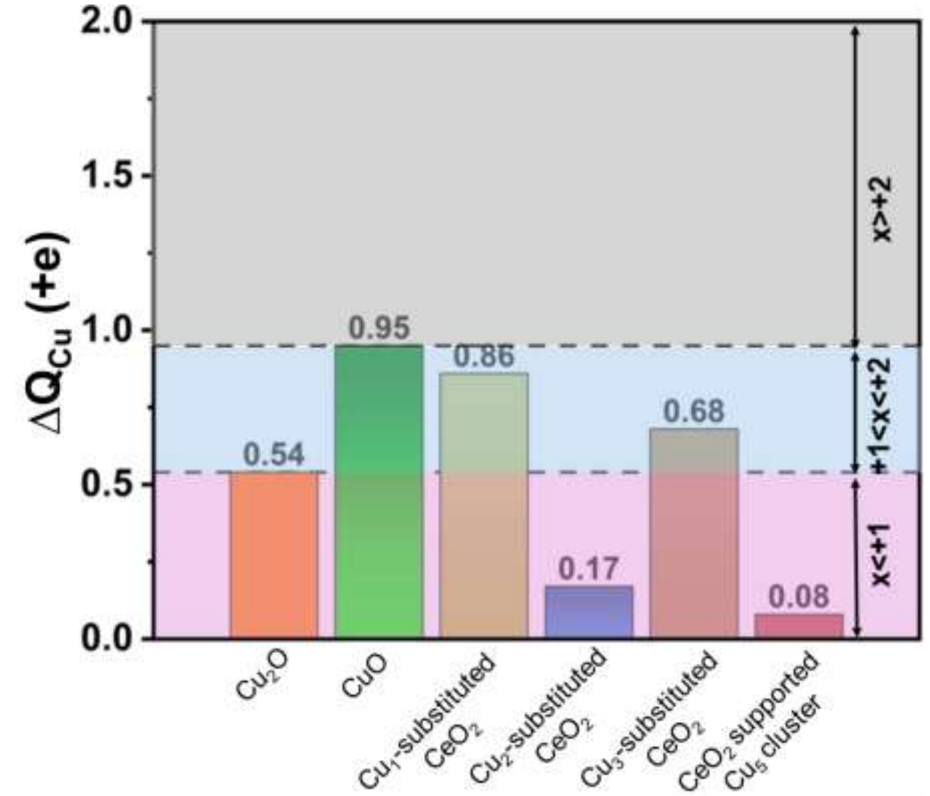

Figure S32. Calculated Bader charge of copper ions in different pristine structures. x indicates the average oxidation state of copper.

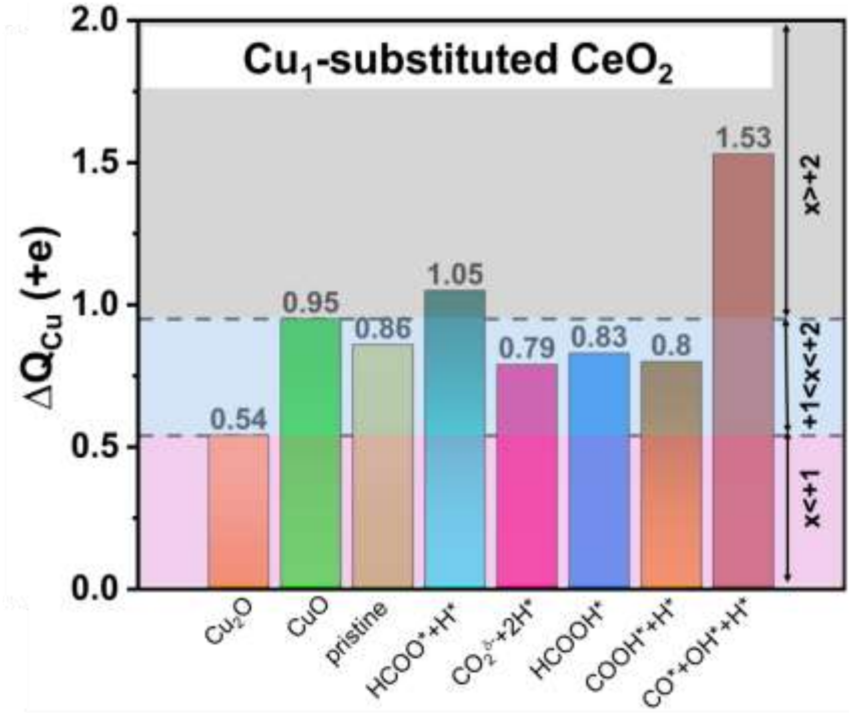

Figure S33. Calculated Bader charge of copper ions in different intermediate adsorbed structures over Cu_1-substituted CeO_2. x indicates the average oxidation state of copper.

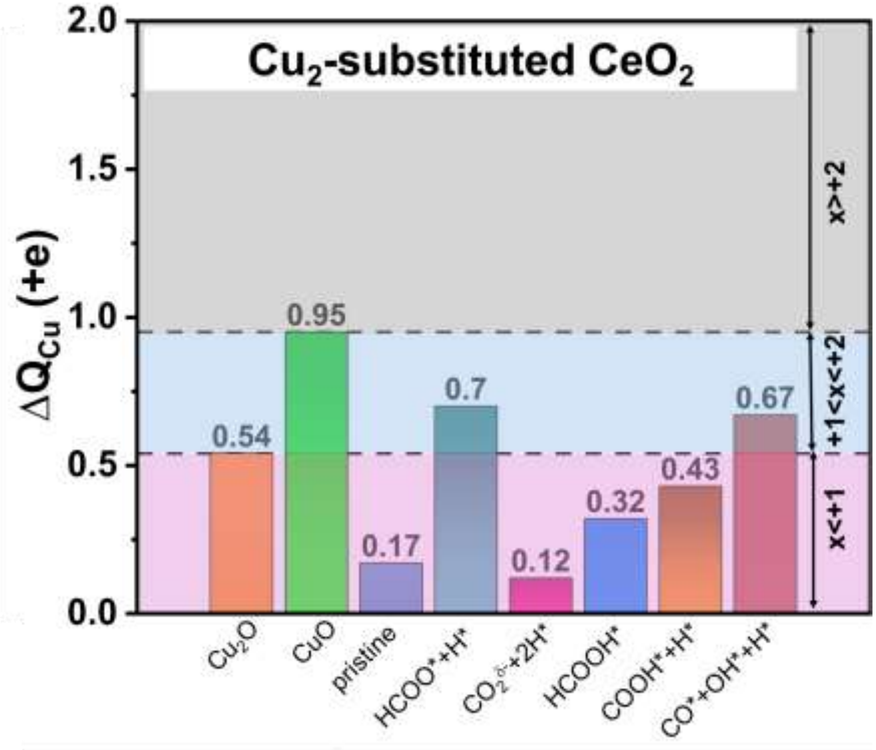

Figure S34. Calculated Bader charge of copper ions in different intermediate adsorbed structures over Cu_2-substituted CeO_2. *x* indicates the average oxidation state of copper.

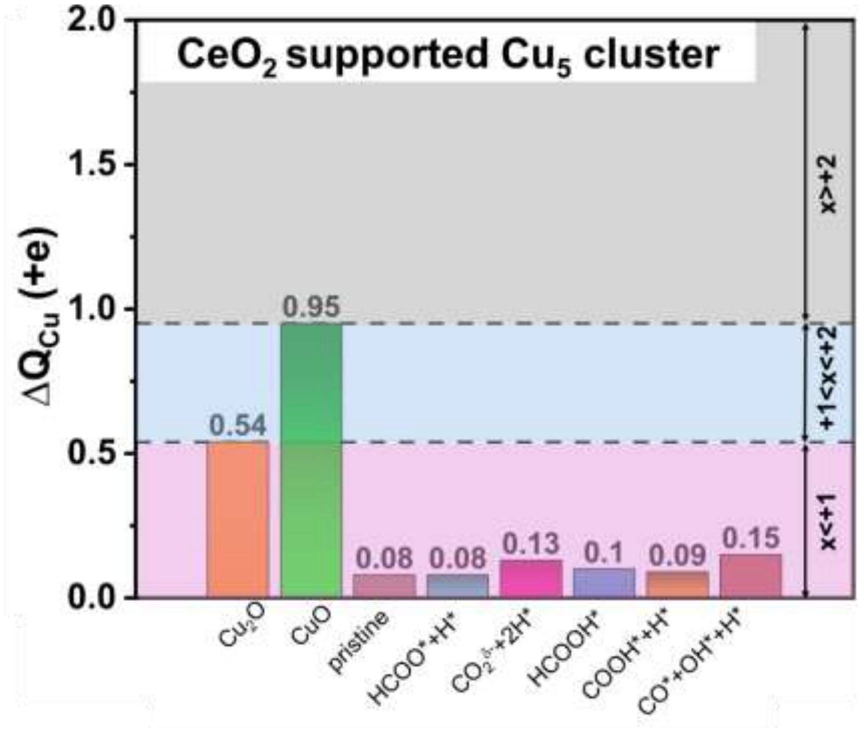

Figure S35. Calculated Bader charge of copper ions in different intermediate adsorbed structures over CeO_2 supported Cu_5 cluster. *x* indicates the average oxidation state of copper.

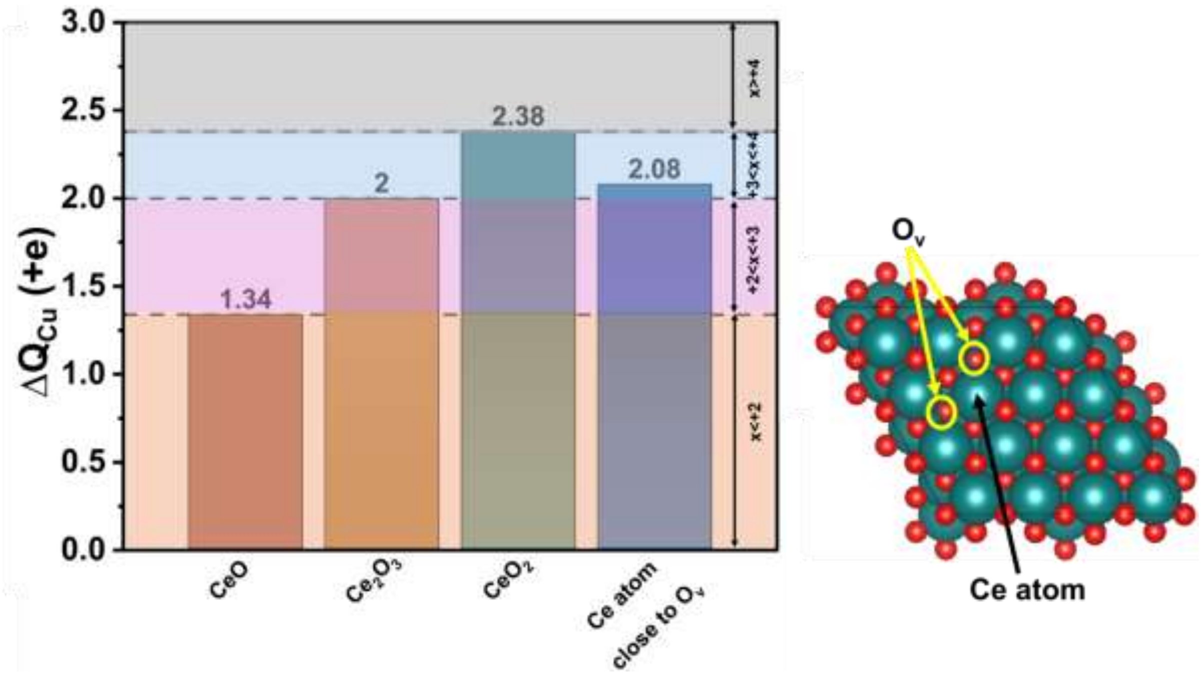

Figure S36. Calculated Bader charge of cerium ions in different structures and the charge of Ce atom located close to the O_V. Our calculations indicate that Ce atoms adjacent to surface oxygen vacancies exhibit Bader charges consistent with Ce^{3+}. This confirms that the Ce^{3+} species are predominantly localized at the surface, where they are accessible to reactants and participate in catalytic reaction. This finding aligns with previous STM study by Esch et al.[13] and DFT calculations by Skorodumova et al.[14], where oxygen vacancies and associated Ce^{3+} were found energetically favored at the surface over the bulk.

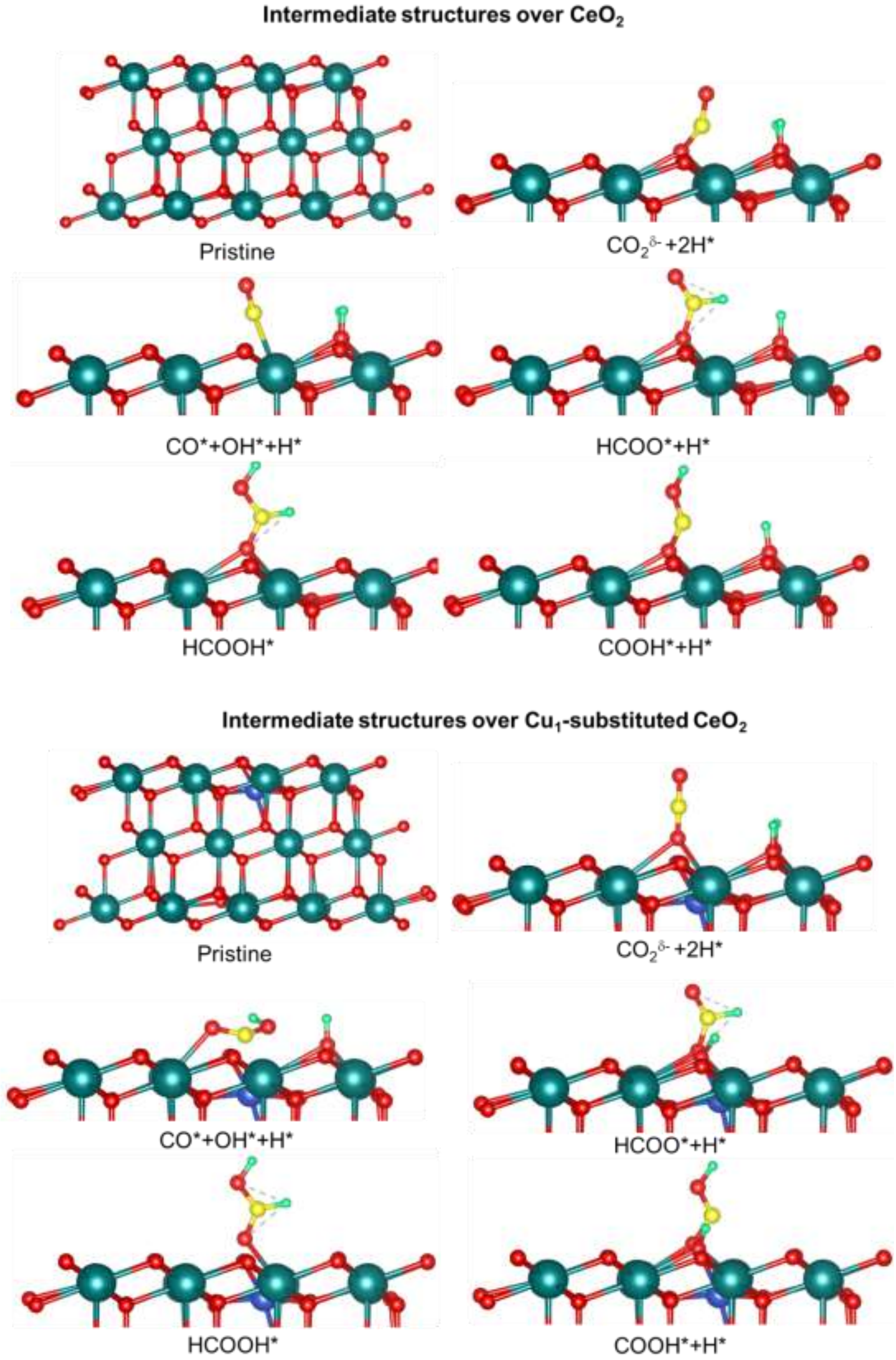

Figure S37. Chemisorbed structure of intermediates on Cu-substituted CeO_2. Blue (Copper), red (Oxygen), cyan (Cerium), yellow (Carbon), bright green (Hydrogen).

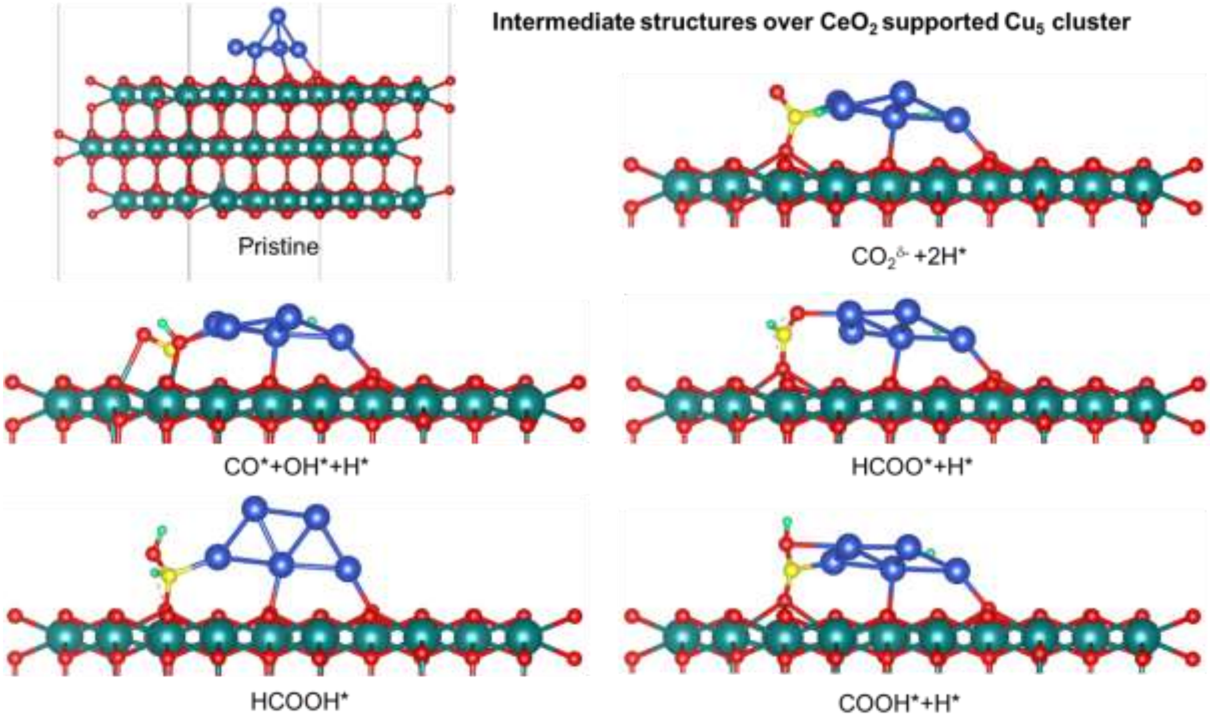

Figure S38. Chemisorbed structure of intermediates on CeO_2-supported Cu_5 cluster. Blue (Copper), red (Oxygen), cyan (Cerium), yellow (Carbon), bright green (Hydrogen).

Table S1. Table summarise the catalytic performance of FSP-prepared catalyst for methanol synthesis from CO_2 hydrogenation.

Sample	Method[a]	Metal loading (wt.%)	Temperature /pressure	H_2/CO_2 ratio	Total GHSV ($L/g_{cat.}/h$)	Conversion	Methanol selectivity	Productivity ($g/h/g_{cat.}$)	REF
**Cu(5)/ZnO-CeO_2(0.05)**	FSP	5	250 °C /30 bar	3	60	~3.4%	~70%	~0.2	15
**Cu(45)/ZnO-CeO_2(0.2)**	FSP	45	250 °C /30 bar	3	120	~3.8%	~50%	~0.58	
**Cu(43)/ZnO/ZrO_2**	FSP	43	235 °C /40 bar	3	75	9.2%	49%	0.5	16
**0.75Pd-5In_2O_3-ZrO_2**	FSP	0.75	280 °C /50 bar	4	48	~12%	87%	1.3	17
**0.75Pd-5In_2O_3-ZrO_2**	WI	0.75	280 °C /50 bar	4	48	7.5%	75%	0.75	
**5In_2O_3-ZrO_2**	FSP	n.a.	280 °C /50 bar	4	48	~3%	~88%	0.35	
**5In_2O_3-ZrO_2**	WI	n.a.	280 °C /50 bar	4	48	~3%	~80%	0.3	
**5In_2O_3-ZrO_2**	CP	n.a.	280 °C /50 bar	4	48	~1.5%	~85%	0.1	
**5ZnO-ZrO_x**	FSP	n.a.	280 °C /50 bar	4	24	7.5%	78%	0.45	18
**5ZnO-ZrO_x**	CP	n.a.	280 °C /50 bar	4	24	4.5%	60%	0.14	
**Ni(6)-In_2O_3(31)-[illegible]**	FSP	6	260 °C /30 bar	3	30	7.5%	51.8%	0.33	19

[a]synthesis method for the catalyst: Flame spray pyrolysis (FSP), wet impregnation (WI), co-precipitation (CP)

Table S2. Catalytic activity of reported rWGS catalyst at 600 ºC and ambient pressure (0.1 MPa).

Sample	Metal loading (wt.%)	CO_2:H_2	CO_2/ total GHSV ($L/g_{cat.}/h$)	Conversion (%)	Selectivity (%)	CO Productivity ($mmol/g_{cat.}/h$)	Ref.
**5CuCe/FSP**	5	1:3	250.5/1062	47.5	100	5312	Our work
			349.5/1458	39.1		6100	
			495/2055	32.3		7131	
			624/2556	27.7		7719	
			750/3060	24.2		8094	
**$Cu_{23}Ce$**	23	1:1	12/48	40	100	214	20
**$Cu_{23}Ce$**	23	1:3	36.4/145.5	38	100	612.5	20
**Cu_3Ce**	3	1:1	12/48	37	100	198	20
**15CuCe**	15	1:3	477.7/2077	24.74	100	5278	21
**$Cu/Ce_{0.05}Mg_{0.95}$**	10	1:3	72/300	58	100	1858	22
**Cu_4/Al_2O_3**	4	1:2	100/300	47	100	2097	23
**Cu_1/Mo_2C**	1	1:2	100/300	40	100	1786	24
**Cr_2O_3/Cu**	17	1:4	30/150	45	100	602	25
**DFNS-TiO_2-Cu10**	10	1:1	1421/2842	10	99.8	5350	26
**Cu_4-ZnO/Al_2O_3**	4	1:2	100/300	45	100	2008	23
**Cu_{34}/SiO_2**	34	1:2	125/625	9.3	100	519	27
**Cu_5-K_1/Mo_2C**	5	1:2.5	24/84	50	100	536	28
**Cu-K/SiO_2**	9	1:1	139.3/278.6	12.8	-	796	29
**50Co/SBA-15**	2.6	1:1	8.1/18	37	99.3	130	30
**Ni_{15}/SiO_2**	15	1:4	80/400	42	-	1500	31
**Mo_2C/SiO_2**	n.a.	1:1	30/60	32	100	429	32
**Mo_2C/SBA-15**	n.a.	1:1	30/60	24	100	321	32
**Mo_2N**	n.a.	1:3	180/720	28	100	2250	33
**H_xMoO_y-600H**	n.a.	1:3	681.6/2840	24.8	100	7545	34
**Mo_2C**	n.a.	1:3	25/100	~58	100	647	35
**Pd-In/SiO_2**	3-10	1:1	12/60	10	100	134	36
**Pt/TiO_2 (HN)**	1	1:3	75/300	44	-	1473	37
**Ir-MoO_3**	0.02	1:3	4800/20000	7.5	100	17430	38
**Ir-MoO_3**	0.02	1:3	240/1000	~28[a]	100	~4000[a]	
**Ir-MoO_3**	0.02	1:3	168/700	~26.7[a]	100	~2000[a]	
**Ir-MoO_3**	0.02	1:3	120/500	~31.7[a]	100	~1700[a]	
**Ir-MoO_3**	0.02	1:3	72/300	~42.9[a]	100	~1379[a]	

[a]Values estimated based on data provided in Figure 1c and d of ref 38

Table S3. Cu loading, Cu/Ce ratio, and quantified surface sites from titration experiments.

Sample	Cu loading (wt.%)	Cu/Ce atomic ratio (from ICP)	Cu/Ce atomic ratio (from XPS)	Exposed Cu^0 (μmol/g)[a]	CO desorption (μmol/g)[b]	CO_2 adsorption sites(μmol/g)[c]	Exposed Cu^+ (μmol/g)[d]	Productivity (mmol/g/h)
2.5CuCe-FSP	2.98	0.08	0.2	296	1155	130	859	2692
5CuCe-FSP	6.21	0.18	0.26	413	1062	165	649	3333
10CuCe-FSP	12.21	0.38	0.32	600	1049	134	449	2978
5CuCe-COP	7.21	0.21	0.14	468	740	136.2	272	2400
5CuSi-FSP	5.36	N.A.	N.A.	N.A.	N.A.	N.A.	N.A.	732

[a]Quantity of Cu^0 sites on the reduced catalyst surface calculated from N_2O titration experiments; [b]CO consumed (via bonding to surface Cu^+ sites) during CO titration experiments over catalyst directly after N_2O titration. This value corresponds to the total number of exposed Cu^+ sites – including those present initially in the reduced catalyst and those formed during N_2O titration ; [c]CO_2 consumed during CO_2 titration experiments; [d]Quantity of exposed Cu^+ sites on the reduced catalyst surface (d=b-a).

Table S4. Quantification of defects (D/F_{2g}) for different catalysts upon switching between different conditions in consecutive order.

D/F_{2g}	2.5CuCe-FSP	5CuCe-FSP	10CuCe-FSP	5CuCe-COP
H_2, 25 °C	0.41	0.86	0.47	1.06
H_2, 400 °C	0.76	1.69	0.68	1.36
CO_2+H_2, 400 °C	0.54	0.84	0.58	1.15
CO_2, 400 °C	0.48	0.51	0.50	1.12
H_2, 400 °C	0.73	1.68	0.71	1.30

Table S5. Reaction order for 5CuCe-COP and 5CuCe-FSP.

	Temperature	Reaction order (CO_2)	Reaction order (H_2)
5CuCe-FSP	500	0.49	0.23
	400	1.1	0.15
5CuCe-COP	500	0.50	0.46
	400	0.39	0.37

Table S6. Calculated free energy values (eV) for different intermediates structures.

CeO_2		Cu_1-substituted CeO_2		CeO_2 supported Cu_5	
**HCOO***	-2.03	**HCOO***	-2.06	**HCOO***	-2.52
**$CO_2^{\delta-}$+2H***	-1.33	**$CO_2^{\delta-}$+2H***	-0.72	**$CO_2^{\delta-}$+2H***	-1.37
**HCOOH***	-0.71	**HCOOH***	-0.71	**HCOOH***	-2.02
**COOH***	-0.73	**COOH***	-0.59	**COOH***	-1.15
**CO*+OH***	-1.84	**CO*+OH***	-0.64	**CO*+OH***	-1.68

S1. Supplementary discussion on Cu oxidation state from DFT:

To gain further insight into the oxidation states of Cu in four models (i to iv) shown in **Figure S31**, we performed Bader charge analysis[11]. While Bader analysis does not directly yield formal oxidation states, it provides a reliable basis for comparison when benchmarked against reference materials (see **Figure S32**). We therefore calculated the Bader charges of Cu in bulk Cu_2O and CuO, corresponding to Cu^+ and Cu^{2+}, respectively, to serve as reference points. In model (ii), the average oxidation state of Cu was found to be between +1 and +2, suggesting more oxidized Cu. In models (i) and (iii), where Cu is present as clusters or is doped in pairs, the average Cu oxidation state lay between 0 and +1, indicating a more reduced environment. In model (iv), combining mono- and di-substituted Cu, the oxidation state was closer to that of Cu_2O (Cu^+), consistent with XPS results for 5CuCe-FSP (**Figure 3b**), where Cu^+ dominates. In contrast, model (i) with Cu clusters correspond well with the more reduced Cu^0-rich surface of 5CuCe-COP.

We also calculated the average oxidation state of Cu atoms in the Cu_5 cluster both in its pristine form and after adsorption of key intermediates (**Figure S35**). While the pristine Cu_5 cluster exhibits an oxidation state close to 0 (metallic), interaction with adsorbates leads to partial oxidation, with average oxidation states shifting slighted toward more oxidized state. This evolution reflects charge transfer between the cluster and the adsorbates.

S2. Supplementary discussion on the CO-FTIR

It is well established that CO binds relatively weakly to Cu species, and well-resolved FTIR-CO features—especially for Cu^0—are typically observed only at cryogenic temperatures (80–220 K) [39,40]. As shown by Chen et al.[39], Cu^+ forms the strongest bonding with CO (~2110 cm^{-1}), which remain detectable even above 300 K, whereas Cu^0–CO interactions are weaker and appear as a shoulder feature below 2100 cm^{-1}. In contrast, CO adsorbed on Ce^{3+}/Ce^{4+} (>2140 cm^{-1}) and Cu^{2+} (>2180 cm^{-1}) generally only appear clearly at much lower temperatures (<120 K).

Given the operational limitation of our FTIR system (room-temperature capability), we conducted CO adsorption experiments at ~300 K. As shown in **Figure S27**, the background spectrum was flat before CO introduction. Upon CO exposure, three distinct bands emerged:

- A sharp peak at ~2125 cm^{-1} (and above) corresponding to gaseous CO,
- A band at 2103 cm^{-1}, which we assign to CO adsorbed on Cu^+,
- A weaker shoulder at ~200 cm^{-1}, which we attentively assign to CO on Cu^0.

Upon switching to He flow, all bands gradually decreased in intensity. Notably, the Cu^+–CO band at 2103 cm^{-1} persisted even after 100 minutes, demonstrating stronger adsorption. Additionally, a feature at 2134–2146 cm^{-1} appeared after 20 minutes, attributed to polycarbonyl $Cu^+(CO)_x$ species, as reported previously[41].

These observations confirm the coexistence of Cu^+ and Cu^0 species on the catalyst surface and reinforce the selective CO binding to Cu^+ at room temperature. This also supports our use of CO chemisorption as a valid approach to titrate Cu^+ species.

S3. Supplementary discussion on the rational for nitric acid leaching to examine the catalytic role of Cu.

Varying the initial Cu loading (e.g., from 2.5 to 10 wt%) affects not only the number of Cu active sites but also alters the Cu geometry (as evidenced by H_2-TPR in **Figure 3d**), oxidation state distribution (as shown by XPS in **Figure S16**), and—importantly—the morphology and defect structure of the CeO_2 support. Specifically, **Figure 2** demonstrates changes in CeO_2 morphology, while the evolution of oxygen vacancies is reflected in the Raman data (**Table S4**), and quantity of CO_2 adsorption sites are summarized in **Table S3**. These intertwined effects make it difficult to isolate the intrinsic role of Cu in rWGS activity without simultaneously modifying the CeO_2 properties.

To address this, we used selective Cu leaching via post-synthetic treatment with 1.5 M HNO_3. This choice is supported by the fact that CeO_2 is notably refractory to dissolution, often requiring stronger acids and elevated temperatures to dissolve[42]. This approach primarily removes surface Cu species while largely preserving the CeO_2 structure in 5CuCe-FSP. After leaching, a slight decrease in surface oxygen vacancies was observed from Raman (**Figure S23a**), while XRD (**Figure S23b**) showed minimal change in crystallinity, with the CeO_2 crystallite size decreasing from 10.84 to 9.63 nm. These confirm that the CeO_2 crystallinity and defect structure remain largely unchanged post-leaching. XPS analysis revealed a small decrease in surface Ce^{3+} content (from 26% to 24.6%, **Figure S23c**), and no detectable Cu 2p signal (**Figure S23d**), indicating that Cu was effectively removed with minimal alteration to the CeO_2 support.

As shown in **Figure S22a**, the nitric acid-leached 5CuCe-FSP catalyst, in which the majority of copper species were removed, retains significant activity at elevated temperatures. This suggests that the ceria matrix itself—particularly the highly defective, reducible nature of the FSP-synthesized support (**Figure S24**)—plays a non-negligible role in CO_2 activation and rWGS activity. The leached sample exhibits a CO production rate of ~3500 mmol $CO/g_{cat.}/h$ at 600 °C, compared to the 5000 mmol $CO/g_{cat.}/h$ achieved by the unleached counterpart. In contrast, the commercial CeO_2 shows negligible activity under identical conditions, demonstrating the crucial role of defect engineering via FSP.

The higher activation energy (89.8 kJ/mol) observed for the leached sample compared to the copper-containing sample indicates that, while FSP ceria contributes to rWGS activity—especially at higher temperatures—it requires more energy to activate reactants in the absence of Cu sites. These findings, together with TPR results (**Figure S24**), support the conclusion that the FSP process yields a superior CeO_2 support with enhanced redox properties and defect density, critical for efficient CO_2 reduction. These control experiments help disentangle the catalytic roles of Cu and CeO_2 and clarify their synergy over 5CuCe-FSP.

S4. Supplementary discussion on post-reaction characterization.

As shown in **Figure S28a**, The Cu 2p XPS spectra show no significant shift in binding energy or peak shape, indicating the preservation of Cu species after the 40-hour reaction. Detailed changes in Cu speciation are discussed in the main manuscript and supported by in-situ UV-Vis results (**Figure 4b**). Additionally, Ce 3d spectra (**Figure S28b**) reveal a decrease in Ce^{3+} content (38.6%) compared to the reduced sample (47.8%), suggesting partial reoxidation due to CO_2 activation—consistent with the in-situ Raman results (**Figure 4a**). STEM images (**Figure S28c**) confirm that Cu remains well dispersed, although ceria particles tend to sinter slightly, forming larger crystallites.

Reference

1. Xie, B.; Tan, T. H.; Kalantar-Zadeh, K.; Zheng, J.; Kumar, P.; Jiang, J.; Zhou, S.; Scott, J.; Amal, R., Promoting low-temperature methanol production over mixed oxide supported Cu catalysts: Coupling ceria-promotion and photo-activation. *Appl. Catal., B* **2022,** *315*, 121599.
2. Zabilskiy, M.; Arčon, I.; Djinović, P.; Tchernychova, E.; Pintar, A., In-situ XAS Study of Catalytic N_2O Decomposition Over CuO/CeO_2 Catalysts. *ChemCatChem* **2021,** *13* (7), 1814-1823.
3. Zabilskiy, M.; Djinović, P.; Tchernychova, E.; Tkachenko, O. P.; Kustov, L. M.; Pintar, A., Nanoshaped CuO/CeO_2 Materials: Effect of the Exposed Ceria Surfaces on Catalytic Activity in N_2O Decomposition Reaction. *ACS Catal.* **2015,** *5* (9), 5357-5365.
4. Koryabkina, N. A.; Phatak, A. A.; Ruettinger, W. F.; Farrauto, R. J.; Ribeiro, F. H., Determination of kinetic parameters for the water–gas shift reaction on copper catalysts under realistic conditions for fuel cell applications. *J. Catal.* **2003,** *217* (1), 233-239.
5. Xie, B.; Wong, R. J.; Tan, T. H.; Higham, M.; Gibson, E. K.; Decarolis, D.; Callison, J.; Aguey-Zinsou, K.-F.; Bowker, M.; Catlow, C. R. A.; Scott, J.; Amal, R., Synergistic ultraviolet and visible light photo-activation enables intensified low-temperature methanol synthesis over copper/zinc oxide/alumina. *Nat. Commun.* **2020,** *11* (1), 1615.
6. Strobel, B. R.; Pratsinis, S. E., Flame Synthesis of Supported Platinum Group Metals for Catalysis and Sensors. *Platinum Metals Review* **2009,** *53* (1), 11-20.
7. Strobel, R.; Pratsinis, S. E.; Baiker, A., Flame-made $Pd/La_2O_3/Al_2O_3$ nanoparticles: thermal stability and catalytic behavior in methane combustion. *J. Mater. Chem.* **2005,** *15* (5), 605-610.
8. Stark, W. J.; Grunwaldt, J.-D.; Maciejewski, M.; Pratsinis, S. E.; Baiker, A., Flame-made Pt/ceria/zirconia for low-temperature oxygen exchange. *Chem. Mater.* **2005,** *17* (13), 3352-3358.
9. Perego, C.; Peratello, S., Experimental methods in catalytic kinetics. *Catal. Today* **1999,** *52* (2-3), 133-145.
10. Shekari, A.; Labrecque, R.; Larocque, G.; Vienneau, M.; Simoneau, M.; Schulz, R., Conversion of CO_2 by reverse water gas shift (RWGS) reaction using a hydrogen oxyflame. *Fuel* **2023,** *344*, 127947.
11. Tang, W.; Sanville, E.; Henkelman, G., A grid-based Bader analysis algorithm without lattice bias. *J. Phys.: Condens. Matter* **2009,** *21* (8), 084204.
12. Biesinger, M. C.; Lau, L. W. M.; Gerson, A. R.; Smart, R. S. C., Resolving surface chemical states in XPS analysis of first row transition metals, oxides and hydroxides: Sc, Ti, V, Cu and Zn. *Appl. Surf. Sci.* **2010,** *257* (3), 887-898.
13. Esch, F.; Fabris, S.; Zhou, L.; Montini, T.; Africh, C.; Fornasiero, P.; Comelli, G.; Rosei, R., Electron Localization Determines Defect Formation on Ceria Substrates. *Science* **2005,** *309* (5735), 752-755.
14. Skorodumova, N. V.; Simak, S. I.; Lundqvist, B. I.; Abrikosov, I. A.; Johansson, B., Quantum Origin of the Oxygen Storage Capability of Ceria. *Phys. Rev. Lett.* **2002,** *89* (16), 166601.
15. Zhu, J.; Ciolca, D.; Liu, L.; Parastaev, A.; Kosinov, N.; Hensen, E. J. M., Flame Synthesis of $Cu/ZnO–CeO_2$ Catalysts: Synergistic Metal–Support Interactions Promote CH_3OH Selectivity in CO2 Hydrogenation. *ACS Catal.* **2021,** *11* (8), 4880-4892.
16. Schulte, M. L.; Catharina Sender, V.; Baumgarten, L.; Beck, A.; Nilayam, A. R. L.; Saraçi, E.; Grunwaldt, J.-D., Tuning Flame Spray Pyrolysis for Variation of the Crystallite Size in $Cu/ZnO/ZrO_2$ and its Influence on the Performance in CO_2-to-Methanol Synthesis. *European Journal of Inorganic Chemistry* **2025,** *28* (4), e202400684.
17. Pinheiro Araújo, T.; Mondelli, C.; Agrachev, M.; Zou, T.; Willi, P. O.; Engel, K. M.; Grass, R. N.; Stark, W. J.; Safonova, O. V.; Jeschke, G.; Mitchell, S.; Pérez-Ramírez, J., Flame-made ternary $Pd-In_2O_3-ZrO_2$ catalyst with enhanced oxygen vacancy generation for CO_2 hydrogenation to methanol. *Nat. Commun.* **2022,** *13* (1), 5610.
18. Pinheiro Araújo, T.; Morales-Vidal, J.; Zou, T.; Agrachev, M.; Verstraeten, S.; Willi, P. O.; Grass, R. N.; Jeschke, G.; Mitchell, S.; López, N.; Pérez-Ramírez, J., Design of Flame-Made $ZnZrO_x$ Catalysts for Sustainable Methanol Synthesis from CO_2. *Adv. Energy Mater.* **2023,** *13* (14), 2204122.

19. Liu, L.; Gao, Y.; Zhang, H.; Kosinov, N.; Hensen, E. J. M., Ni and ZrO_2 promotion of In_2O_3 for CO_2 hydrogenation to methanol. *Applied Catalysis B: Environment and Energy* **2024,** *356*, 124210.
20. Kim, G.; Ryu, S. H.; Jeong, H.; Choi, Y.; Lee, S.; Choi, J. H.; Lee, H., Easily Scalable Shell-Structured Copper Catalyst with High Activity and Durability for Carbon Dioxide Hydrogenation. *Angew. Chem.* **2023,** *135* (30), e202306017.
21. Liu, H.-X.; Li, S.-Q.; Wang, W.-W.; Yu, W.-Z.; Zhang, W.-J.; Ma, C.; Jia, C.-J., Partially sintered copper–ceria as excellent catalyst for the high-temperature reverse water gas shift reaction. *Nat. Commun.* **2022,** *13* (1), 867.
22. Li, S.; Liu, X.; Ma, J.; Xu, F.; Lyu, Y.; Perathoner, S.; Centi, G.; Liu, Y., Develop High-Performance Cu-Based RWGS Catalysts by Controlling Oxide–Oxide Interface. *ACS Catal.* **2025,** *15* (4), 3475-3486.
23. Bahmanpour, A. M.; Héroguel, F.; Kılıç, M.; Baranowski, C. J.; Artiglia, L.; Röthlisberger, U.; Luterbacher, J. S.; Kröcher, O., Cu–Al spinel as a highly active and stable catalyst for the reverse water gas shift reaction. *ACS Catal.* **2019,** *9* (7), 6243-6251.
24. Zhang, X.; Zhu, X.; Lin, L.; Yao, S.; Zhang, M.; Liu, X.; Wang, X.; Li, Y.-W.; Shi, C.; Ma, D., Highly dispersed copper over β-Mo_2C as an efficient and stable catalyst for the reverse water gas shift (RWGS) reaction. *ACS Catal.* **2017,** *7* (1), 912-918.
25. Shen, Y.; Xiao, Z.; Liu, J.; Wang, Z., Facile preparation of inverse nanoporous Cr_2O_3/Cu catalysts for reverse water-gas shift reaction. *ChemCatChem* **2019,** *11* (22), 5439-5443.
26. Belgamwar, R.; Verma, R.; Das, T.; Chakraborty, S.; Sarawade, P.; Polshettiwar, V., Defects Tune the Strong Metal–Support Interactions in Copper Supported on Defected Titanium Dioxide Catalysts for CO_2 Reduction. *J. Am. Chem. Soc.* **2023**.
27. Jin, R.; Easa, J.; O'Brien, C. P., Highly active CuO_x/SiO_2 dot core/rod shell catalysts with enhanced stability for the reverse water gas shift reaction. *ACS Appl. Mater. Interfaces* **2021,** *13* (32), 38213-38220.
28. Xu, J.; Gong, X.; Hu, R.; Liu, Z.-w.; Liu, Z.-t., Highly active K-promoted Cu/β-Mo_2C catalysts for reverse water gas shift reaction: Effect of potassium. *Mol. Catal.* **2021,** *516*, 111954.
29. Chen, C.-S.; Cheng, W.-H.; Lin, S.-S., Study of reverse water gas shift reaction by TPD, TPR and CO2 hydrogenation over potassium-promoted Cu/SiO_2 catalyst. *Appl. Catal., A* **2003,** *238* (1), 55-67.
30. Liang, H.; Zhang, B.; Gao, P.; Yu, X.; Liu, X.; Yang, X.; Wu, H.; Zhai, L.; Zhao, S.; Wang, G.; van Bavel, A. P.; Qin, Y., Strong Co-O-Si bonded ultra-stable single-atom Co/SBA-15 catalyst for selective hydrogenation of CO_2 to CO. *Chem Catalysis* **2022,** *2* (3), 610-621.
31. Galhardo, T. S.; Braga, A. H.; Arpini, B. H.; Szanyi, J.; Gonçalves, R. V.; Zornio, B. F.; Miranda, C. R.; Rossi, L. M., Optimizing active sites for high CO selectivity during CO_2 hydrogenation over supported nickel catalysts. *J. Am. Chem. Soc.* **2021,** *143* (11), 4268-4280.
32. Marquart, W.; Raseale, S.; Prieto, G.; Zimina, A.; Sarma, B. B.; Grunwaldt, J.-D.; Claeys, M.; Fischer, N., CO_2 Reduction over Mo_2C-Based Catalysts. *ACS Catal.* **2021,** *11* (3), 1624-1639.
33. Wu, Y.; Xie, Z.; Gao, X.; Zhou, X.; Xu, Y.; Fan, S.; Yao, S.; Li, X.; Lin, L., The highly selective catalytic hydrogenation of CO_2 to CO over transition metal nitrides. *Chinese Journal of Chemical Engineering* **2022,** *43*, 248-254.
34. Du, X.; Li, R.; xin, H.; Fan, Y.; Liu, C.; Feng, X.; wang, j.; Dong, C.; Wang, C.; Li, D.; Fu, Q.; Bao, X., In-Situ Dynamic Carburization of Mo Oxide with Unprecedented High CO Formation Rate in Reverse Water-Gas Shift Reaction. *Angew. Chem. Int. Ed.* **2024,** *63* (51), e202411761.
35. Ahmadi Khoshooei, M.; Wang, X.; Vitale, G.; Formalik, F.; Kirlikovali, K. O.; Snurr, R. Q.; Pereira-Almao, P.; Farha, O. K., An active, stable cubic molybdenum carbide catalyst for the high-temperature reverse water-gas shift reaction. *Science* **2024,** *384* (6695), 540-546.
36. Ye, J.; Ge, Q.; Liu, C.-j., Effect of PdIn bimetallic particle formation on CO_2 reduction over the Pd–In/SiO_2 catalyst. *Chem. Eng. Sci.* **2015,** *135*, 193-201.
37. Chen, Z.; Liang, L.; Yuan, H.; Liu, H.; Wu, P.; Fu, M.; Wu, J.; Chen, P.; Qiu, Y.; Ye, D., Reciprocal regulation between support defects and strong metal-support interactions for highly efficient reverse water gas shift reaction over Pt/TiO_2 nanosheets catalysts. *Appl. Catal., B* **2021,** *298*, 120507.

38. Sun, X.; Yu, J.; Zada, H.; Han, Y.; Zhang, L.; Chen, H.; Yin, W.; Sun, J., Reaction-induced unsaturated Mo oxycarbides afford highly active CO_2 conversion catalysts. *Nat. Chem.* **2024**.
39. Chen, A.; Yu, X.; Zhou, Y.; Miao, S.; Li, Y.; Kuld, S.; Sehested, J.; Liu, J.; Aoki, T.; Hong, S., Structure of the catalytically active copper–ceria interfacial perimeter. *Nat. Catal.* **2019,** *2*, 334-341.
40. Drenchev, N.; Ivanova, E.; Mihaylov, M.; Hadjiivanov, K., CO as an IR probe molecule for characterization of copper ions in a basolite C300 MOF sample. *Phys. Chem. Chem. Phys.* **2010,** *12* (24), 6423-6427.
41. Hadjiivanov, K. I.; Vayssilov, G. N., Characterization of oxide surfaces and zeolites by carbon monoxide as an IR probe molecule. **2002**.
42. Virot, M.; Chave, T.; Horlait, D.; Clavier, N.; Dacheux, N.; Ravaux, J.; Nikitenko, S. I., Catalytic dissolution of ceria under mild conditions. *J. Mater. Chem.* **2012,** *22* (29), 14734-14740.
